# Transition-Metal-Free Continuous-Flow Synthesis of 2,5-Diaryl Furans: Access to Medicinal Building Blocks and Optoelectronic Materials

**DOI:** 10.1021/acs.joc.3c02237

**Published:** 2023-12-25

**Authors:** Helena F. Grantham, Robert J. Lee, Grzegorz M. Wardas, Jai-Ram Mistry, Mark R. J. Elsegood, Iain A. Wright, Gareth J. Pritchard, Marc C. Kimber

**Affiliations:** † Department of Chemistry, School of Science, Loughborough University, Loughborough LE11 3TU, U.K.; ‡ The School of Chemistry, University of Edinburgh, Joseph Black Building, Edinburgh EH9 3FJ, U.K.

## Abstract

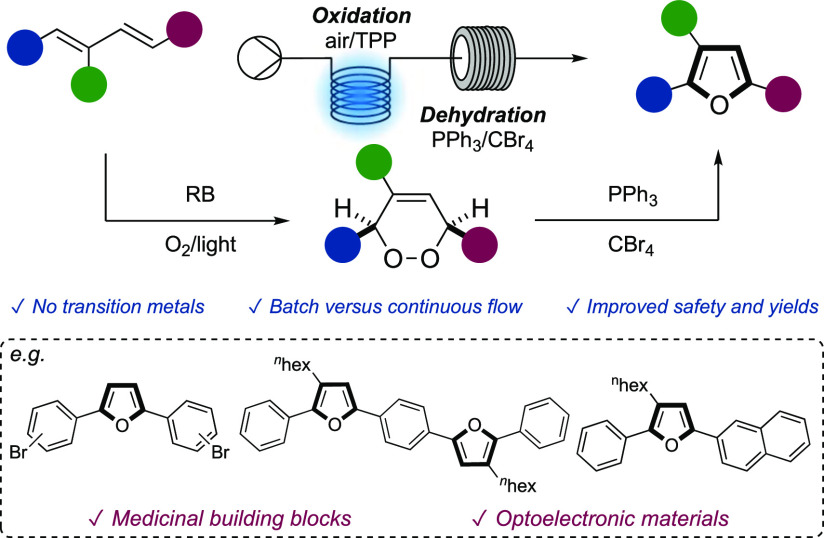

The direct transformation of 1,3-dienes into valuable 2,5-diarylfurans using transition-metal-free conditions is presented. By employing a simple oxidation—dehydration sequence on readily accessible 1,3-dienes, important 2,5-diarylfuran building blocks frequently used in medicinal and material chemistry are prepared. The oxidation step is realized using singlet oxygen, and the intermediate endoperoxide is dehydrated under metal-free conditions and at ambient temperature using the Appel reagent. Notably, this sequence can be streamlined into continuous flow, thereby eliminating the isolation of the intermediate, often unstable endoperoxide. This leads to a significant improvement in isolated yields (ca. 27% average increase) of the 2,5-diarylfurans while also increasing safety and reducing waste. Our transition-metal-free synthetic approach to 2,5-diarylfurans delivers several important furan building blocks used commonly in medicinal chemistry and as optoelectronic materials, including short-chain linearly conjugated furan oligomers. Consequently, we also complete a short study of the optical and electrochemical properties of a selection of these novel materials.

## Introduction

**Figure 1:**
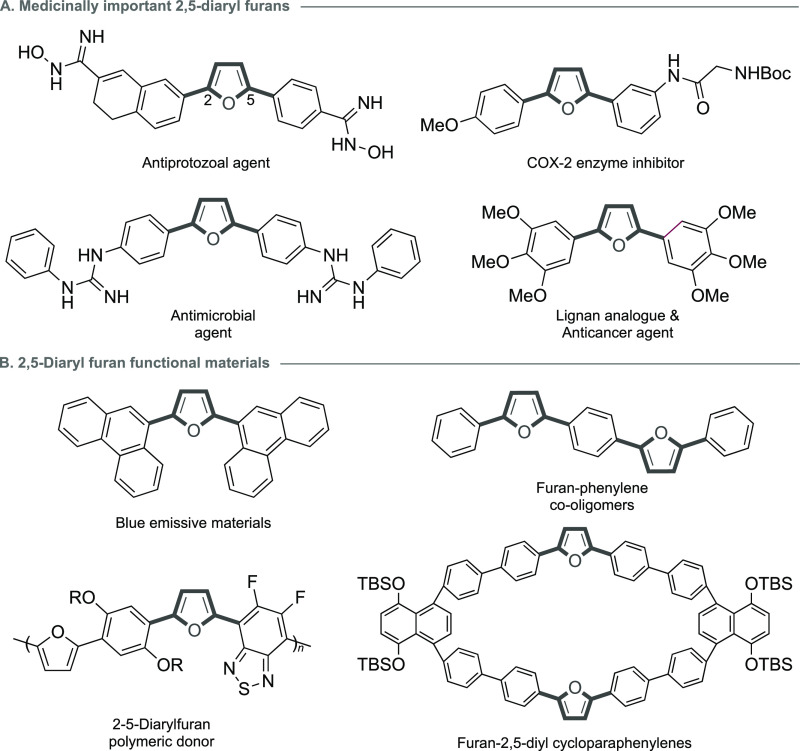
2,5-Diaryl furans: (A) medicinally relevant substrates and (B) functional materials.

The furan ring is a highly important class of heterocycle, frequently found in natural products, several on-market pharmaceuticals, as well as being used in material chemistry.^1a−1e^ Within this class, 2,5-diaryl-substituted furans (Figure 1A) are becoming a privileged scaffold in medicinal chemistry, acting as DNA minor groove binders,^2a−2d^ RNA binders,^2e^ displaying antimicrobial and anticancer activity,^2f,2g^ as well as analogues possessing COX-2 enzyme inhibitory activity.^2h^ More recently, several 2,5-diaryl furans have been developed as blue emissive materials,^3a^ polymeric oligo-donors and co-oligomers,^3b−3g^ and as potential photonic chromophores (Figure 1B).^3h^

The orthodox preparation of 2,5-diarylfurans is by the Paal–Knorr synthesis and requires access to the requisite 1,4-dicarbonyl precursor, preparation of which can be lengthy, particularly if unsymmetrical 2,5-diaryl furans are the target.^4a−4d^ Accordingly, several approaches to the preparation 2,5-diarylfurans using transition metals such as Cu, Ag, Pd, Au, and Zn have been developed.^3a,5a−5j^ Recently, C6 biomass-derived furfural building blocks such as 5-hydroxymethyl furfural and 2,5-furandicarboxylic acid have proven to be excellent substrates to access symmetrical and unsymmetrical 2,5-diaryl furans using metal-catalyzed decarboxylative cross-coupling, although these approaches utilized high loadings of a Pd catalyst.^6a−6d^ Yet, despite the success of these approaches to the preparation of 2,5-diaryl furans, there has been a growing necessity to reduce our reliance on transition-metal-catalyzed reactions.^7^ Several of the commonly used transition metals (e.g., Pd) are experiencing reduced availability leading to higher costs; this is in part due to natural and strategic resource limitations. Their toxicity is also a limitation, often requiring regulation of their permitted levels and strict controlling to parts per million amounts in APIs.

**Scheme 1:**
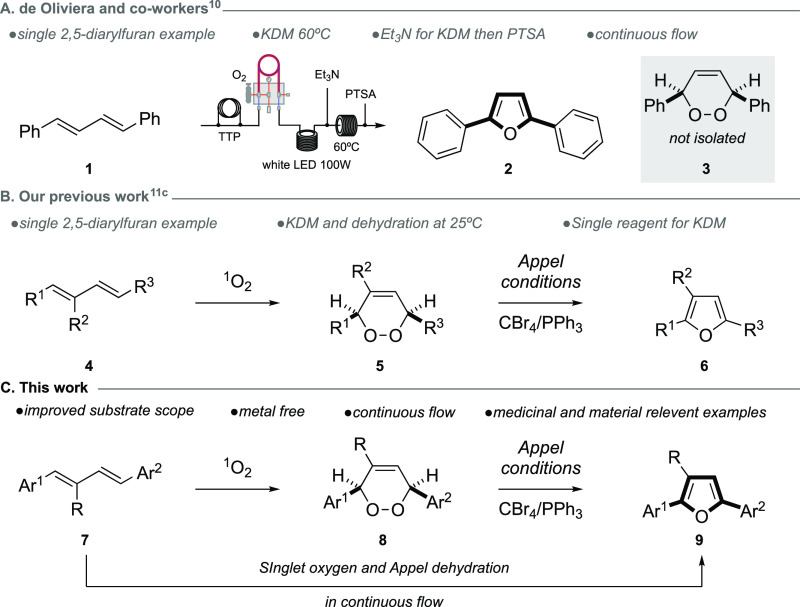
(A) Continuous-Flow Synthesis of 2,5-Diphenylfuran; (B) Appel-Mediated Dehydration of Endoperoxides; and (C) New Route to 2,5-Diarylfurans in Batch and Continuous Flow

An efficient, yet underused, approach to the preparation of furans is the oxidation of a 1,3-diene precursor to an endoperoxide with subsequent dehydration.^8a−8f^ There are intermittent reports of the direct conversion of endoperoxides to furans using metal-free conditions,^8f,9a−9h^ but generally these sporadic examples suffer from limited substrate scope, particularly in accessing 2,5-diaryl furans. A notable example by de Oliviera and coworkers provided an innovative conversion of commercially available diene **1** to 2,5-diphenyl furan (**2**) (Scheme 1A). Importantly, this was achieved using continuous-flow technology, without isolation of the intermediate endoperoxide **3**; however, only a single 2,5-diphenylfuran example was reported (Scheme 1A).^10^ Recently, our group has reported the synthesis of several furan fatty acid natural products and metabolites,^11a,11b^ including a biomimetic approach to the furan fatty acid 11M5 (F5) and moracin M.^11c,11d^ Key to this approach was the oxidation of a 1,3-diene precursor (**4**) using ^1^O_2_, followed by mild dehydration of the subsequent endoperoxide (**5**) to furan (**6**) using Appel conditions via a Kornblum DeLaMare rearrangement (Scheme 1B).^12a,12b^ Crucially, we recognized that the oxidation of a 1,3-diene using ^1^O_2_ circumvented the preparation of the somewhat problematic 1,4-carbonyl precursors.

Therefore, in this disclosure, we report a “transition-metal-free” synthesis of symmetrical and unsymmetrical 2,5-diarylfurans (**9**) from 1,3-dienes (**7**) (Scheme 1C). First, we provide a simple 3-step protocol providing symmetrical and unsymmetrical precursor 1,3-dienes (**7**) with suitable functionality on the arene rings. The oxidation and Appel dehydration are then performed providing 2,5-diaryl furans (**9**) in good to excellent yields over these two steps affording several strategic furanyl building blocks which could find use in either medicinal or material chemistry. Given that the solubility of 2,5-diaryl furans in material chemistry is often challenging, an alkyl chain at the 3-position is incorporated to improve solubility, and we demonstrate its applicability by presenting a metal-free synthesis of a novel furan phenylene co-oligomer. By using continuous-flow technology, we convert these 1,3-dienes into 2,5-diaryl furans, without having to isolate the intermediate endoperoxide (**8**). Finally, the optoelectronic properties of several synthesized 2,5-diaryl furans are examined and discussion provided to the merits of transition-metal-free syntheses.

## Results and Discussion

### Batch Synthesis

**Scheme 2:**
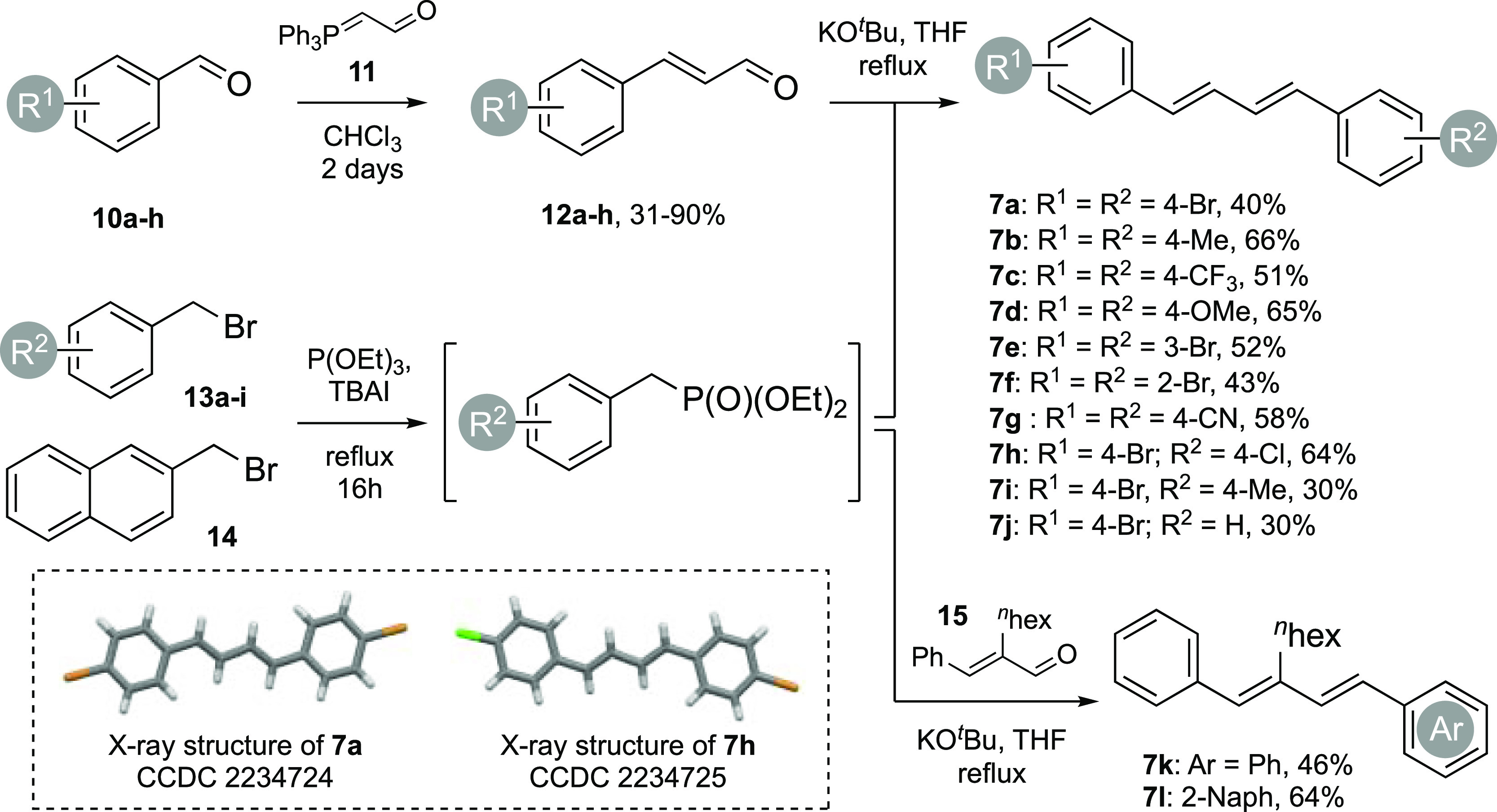
Synthetic Sequence Used to Access the 1,3-Diene Precursors. For X-ray Structures, the Displacement Ellipsoids Are Shown at the 50% Probability Level

Our study began with the synthesis of the precursor 1,3-dienes (Scheme 2). The synthetic route starts from commercially available benzaldehydes (**10a**–**h**) and involved an initial Wittig homologation with commercial ylide **11**, providing the cinnamaldehyde derivatives (**12a**–**h**) in good, isolated yields of 31–90%. An Arbuzov phosphonate formation with several commercially available benzyl bromides (**13a**–**i**) then provided the coupling partner for a subsequent HWE reaction with **12a**–**h**. This reaction sequence provided symmetrical (**7a**–**d**, **7g**) and unsymmetrical (**7h**–**j**) 4-aryl substituted 1,3-dienes with varying functionality (Br, Cl, CN, Me, OCH_3_, and CF_3_), as well as two symmetrical 3-bromophenyl and 2-bromophenyl-1,3-dienes (**7e, f**). The symmetrical 4-bromoaryl-1,3-diene **7a** and the unsymmetrical 1,3-diene **7h** provided crystals suitable for X-ray analysis. Using the same sequence, an additional two bis-aryl 1,3-dienes, substituted at the 2-position with hexyl side chains (**16a, b**), were synthesized from commercially available cinnamaldehyde **15** and bromides **13a** and **14** to improve solubility. This selection of 1,3-dienes provided a good cross section of viable precursors for medicinal and material purposes with added functionality that could plausibly undergo further synthetic transformations, and full experimental details can be found in the Supporting Information.

**Scheme 3:**
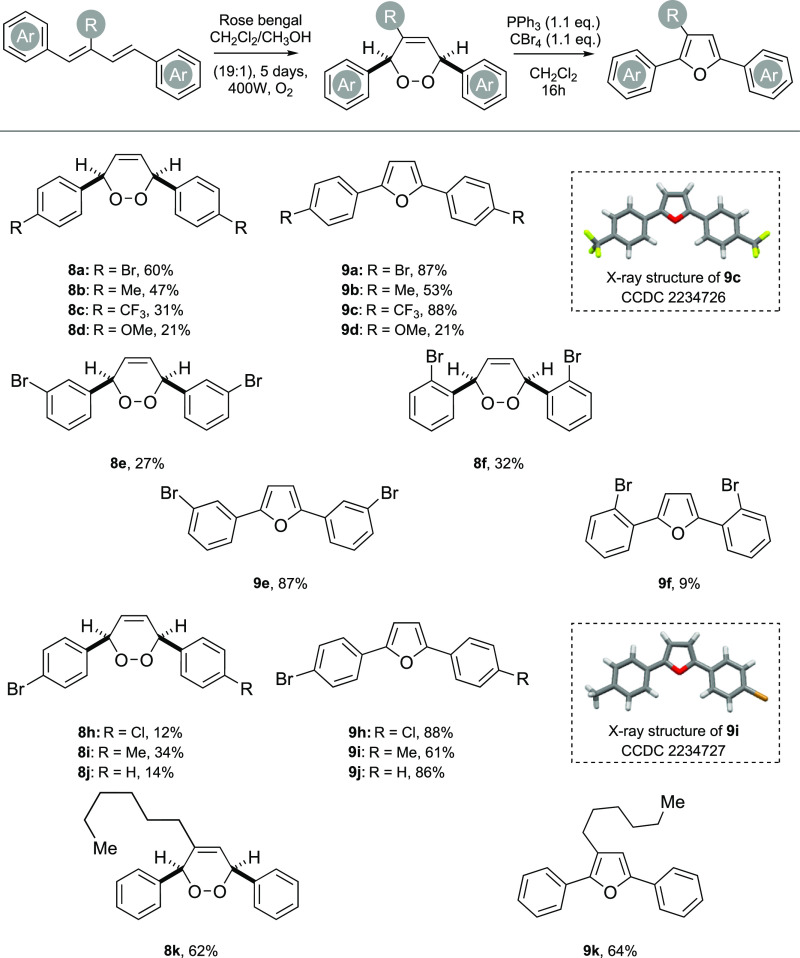
Substrate Scope and Synthesis of 2,5-Diaryl Furans. For X-ray Structures, the Displacement Ellipsoids Are Shown at the 50% Probability Level

With the precursor dienes in-hand, we first examined the substrate scope in batch by performing, stepwise, the oxidation of each diene to their endoperoxide, followed by subsequent dehydration using Appel conditions. The conditions selected were based on our previous disclosure using rose Bengal as the sensitizer for the oxidation step, for a 5 day period, which is broadly in line with our own previous work,^11c^ and 1.1 equiv of the Appel reagent (CBr_4_/PPh_3_) for the dehydration to furan (Scheme 3).

The symmetrical 1,3-dienes **7a**–**d** were oxidized in moderate isolated yields to their, respective, endoperoxides (**8a**–**d**), with the 4-bromo substrate performing the best. The dehydration of each of the symmetrical endoperoxides was then achieved using the Appel conditions with the 4-bromo (**8a**) and 4-trifluoromethyl (**8c**) substrates giving the furans **9a** and **9c** in yields of 87% and 88%, respectively. Additionally, the 4-trifluoromethyl furan **9c** provided crystals suitable for X-ray analysis, supporting the spectral data. In contrast, when endoperoxides **8b** and **8d** were dehydrated, it provided furans **9b** and **9d** in reduced isolated yields of 53 and 21%, respectively. We can account for this by the reduced acidity of the benzylic C–H adjacent to the peroxide, a consequence of the electron donating 4-CH_3_ and 4-OCH_3_ groups on the arene; this bond is key in facilitating the peroxide bond-breaking step in the KDM rearrangement.^11c^ The 3-bromoaryl (**7e**) and 2-bromoaryl dienes (**7f**) provided the endoperoxides **8e** and **8f** in modest yields, with dehydration of **8e** giving furan **9e** in 87% yield, but **8f** gave furan **9f** in a poor yield of 9%. Again, this is a consequence of steric impingement by the ortho bromides in the key peroxide fragmentation step in the proposed mechanism.^11c^ Unfortunately, all attempts at oxidizing the 4-nitrile 1,3-diene **7g** resulted in degradation, with none of the endoperoxide being detected. Oxidation of unsymmetrical dienes **7h**–**j** gave the endoperoxides **8h**–**j** in modest yield, with dehydration of **8h** providing furan **9h** in 88%, **8i** giving furan **9i** in 61%, and **8j** giving **9j** in 86% isolated yield. Furan **9h** also provides a furan building block with potential chemoselective synthetic handles. Finally, 1,3-diene **7k**, which contains a hexyl chain at the 3-position, could be oxidized and dehydrated successfully to provide furan **9k** in 64% yield.

**Scheme 4:**
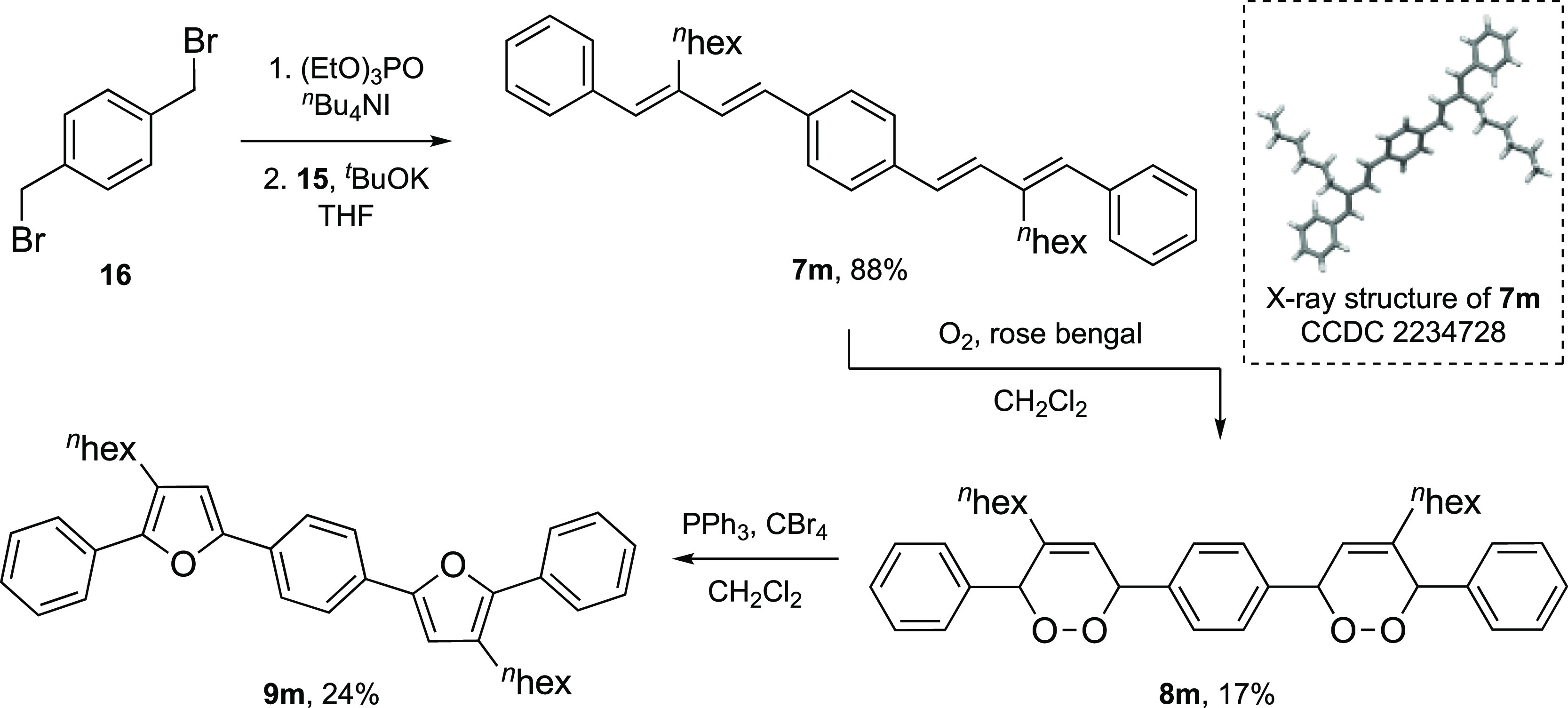
Synthesis of a Furan-Phenylene Co-Oligomer. For X-ray Structure, the Displacement Ellipsoids Are Shown at the 50% Probability Level

This sequential oxidation/dehydration sequence when performed on 1,3-dienes does provide several suitably substituted furans that are convenient synthetic building blocks for accessing furan-based medicinal and material targets. To further highlight the potential of the sequence, we targeted a metal-free synthesis of a soluble furan-phenylene material (Scheme 4). Short-chain linearly conjugated oligomers represent an important class of organic semiconductor; however, their synthesis typically relies upon traditional transition-metal cross-coupling approaches. Oligofurans present several important and useful differences to thiophene-based materials such as efficient luminescent properties and biodegradability. However, they remain much less widely studied in comparison. This is due in no small part to challenges associated with oxidative instability of many furan-based intermediates required to produce these compounds, which can complicate standard iterative approaches.^13a−13c^ Therefore, having a wider variety of reliable routes, such as that presented here, to incorporate furans into larger conjugated systems is important.

The synthesis began with preparation of the bis-diene **7m** using a HWE reaction of bis-phosphonate prepared from bromide **16** and commercially available cinnamaldehyde **15**, which provided **7m** in 88% yield over the two steps; this material was crystalline, and its proposed structure was further supported by X-ray crystallography. Photooxidation of **7m** with ^1^O_2_ then provided bis-endoperoxide **8m** as a mixture of stereoisomers in 17% yield. This unusual bis-endoperoxide **8m** was then dehydrated to provide furan-phenylene **9m** in 24% yield.

### Continuous-Flow Synthesis

**Scheme 5:**
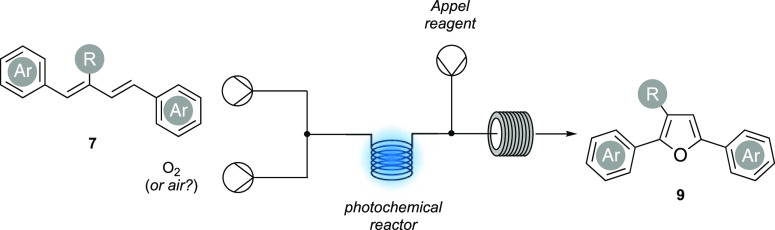
An Idealized Continuous-Flow Synthesis of 2,5-Diarylfurans

The results in Schemes 3 and 4 show that the oxidation of each 1,3-diene substrate was challenging, with only **8a** and **8k** being isolated in yields of more than 50%. The stability of each of the synthesized endoperoxides also proved capricious, with difficult purification and poor stability and, as a result, the endoperoxide products were used immediately in the subsequent dehydration step. Therefore, to overcome these issues, we sought to place our synthetic sequence (oxidation/dehydration) into continuous flow. We envisaged performing the initial photooxidation of **7** in-flow using a commercial off-the-shelf flow photochemical platform^14a,14b^ and then telescoping this into a subsequent reactor to perform the Appel dehydration step, providing furan **9** (Scheme 5).

This would circumvent isolation of the endoperoxide, improve safety, reduce waste, and finally, streamline the synthesis of the 2,5-diaryl furan targets. However, in our approach, there were several issues that needed addressing, such as the compatibility of the oxidation step with the Appel reaction and the P(V) species, as well as ensuring the flow rate for the oxidation and the dehydration step was matched. When approaching the transfer of the ^1^O_2_/Appel procedure to a continuous-flow setup, each reaction was treated separately before being telescoping into a multistep process. This would allow each step to be examined in a continuous-flow setup in isolation to ensure optimal conditions were obtained for each. For the batch process (Scheme 3), the photosensitizer selected was rose Bengal as we used a 19:1 (CH_2_Cl_2_/CH_3_OH) solvent system. Given that the presence of CH_3_OH would affect the Appel reaction, we elected to use a single-solvent system (CH_2_Cl_2_) which necessitated a switch to tetraphenylporphyrin (TPP) as the photosensitizer. Also, since our commercially purchased flow platform used peristaltic pumps, we considered using atmospheric air in place of oxygen.

Therefore, using an initial concentration of 0.025 M of diene **1** in CH_2_Cl_2_ containing TPP at 10^–4^ M and an initial **1**/TPP flow rate of 0.30 mL min^–1^, the optimum conditions found for conversion to endoperoxide **3** were with an air flow rate of 1.85 mL min^–1^ and using a back pressure regulator (BPR) set at 9 bar with irradiation at 450 nm in the photochemical coil reactor (Table 1). These conditions ensured segmented flow within the reactor and sufficient oxygen, given the stoichiometry of the reaction and the concentration of oxygen in air, as well as a significant reduction in reaction time compared to the batch conditions. This provided **3** in a yield of 68% which was comparable with the batch yield of 65% for **3** using rose Bengal (Table 1, entry 1).^15^ Effective air flow was vital for the oxidation; reducing the rate to 1.50 mL min^–1^ resulted in **3** being isolated in 66% yield (entry 2), and a further drop to 60% yield was observed at a flow rate of 1.10 mL min^–1^ (entry 3). The back pressure was also important, with a reduction in pressure to 5 bar and an air flow rate of 1.10 mL min^–1^, seeing the yield of **3** dropping to 54% (entry 4) while performing the flow reaction with no BPR giving just a 25% yield of **3** (entry 5). All attempts at reducing the flow rate of the **1**/TTP solution below the 0.30 mL min^–1^ threshold resulted in significant amounts of “back pumping” at both 9 and 5 bar, respectively. Finally, the reaction did not proceed in the absence of TPP or in the absence of irradiation at 450 nm (entries 6 and 7).

**Table 1:**
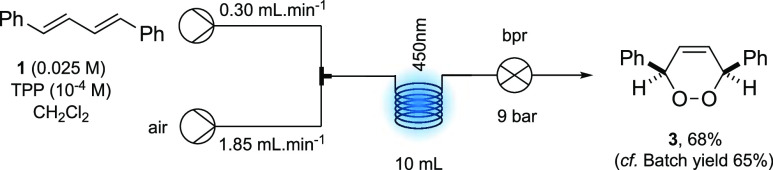
Continuous-Flow Optimization of **3**^a^

entry	variation from optimal conditions	yield **3** (%)^b^
1	none	68
2	air flow rate 1.50 mL min^–^^1^, BPR 9 bar	66
3	air flow rate 1.10 mL min^–^^1^, BPR 9 bar	60
4	air flow rate 1.10 mL min^–^^1^, BPR 5 bar	54
5	air flow rate 1.10 mL min^–^^1^, no BPR	25
6	no TPP	
7	no irradiation	

a Performed on 0.25 mmol **1**.

b Isolated yields.

**Scheme 6:**
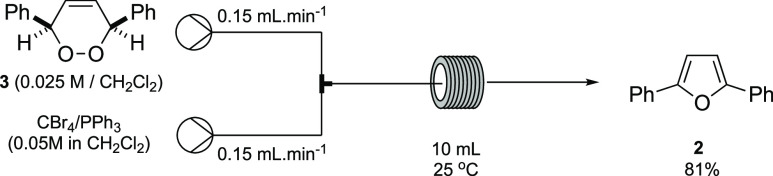
Optimal Continuous-Flow Synthesis of 2,5-Diphenylfuran **2**

Attention was then turned to transferring the batch Appel-type dehydration to a continuous-flow setup. The optimal conditions for the dehydration in continuous flow used a solution of **3** in CH_2_Cl_2_ (0.025 M) pumped at 0.15 mL/min combined with a stream of freshly generated Appel reagent in CH_2_Cl_2_ (0.05 M) also pumped at 0.15 mL/min (Scheme 6). This was then fed into a 10 mL reactor coil maintained at 25 °C giving furan **2** in 81%. A variation in the stoichiometry, by lowing the concentration of the Appel reagent to 0.03 M, resulted in a slight decrease in yield to 79%, while an increase in flow rate from 0.15 to 0.30 mL min^–1^ saw the yield of **2** being maintained at 76%. This latter result is important given that flow rates would need to be matched at 0.30 mL min^–1^ for the telescoped process.

**Scheme 7:**
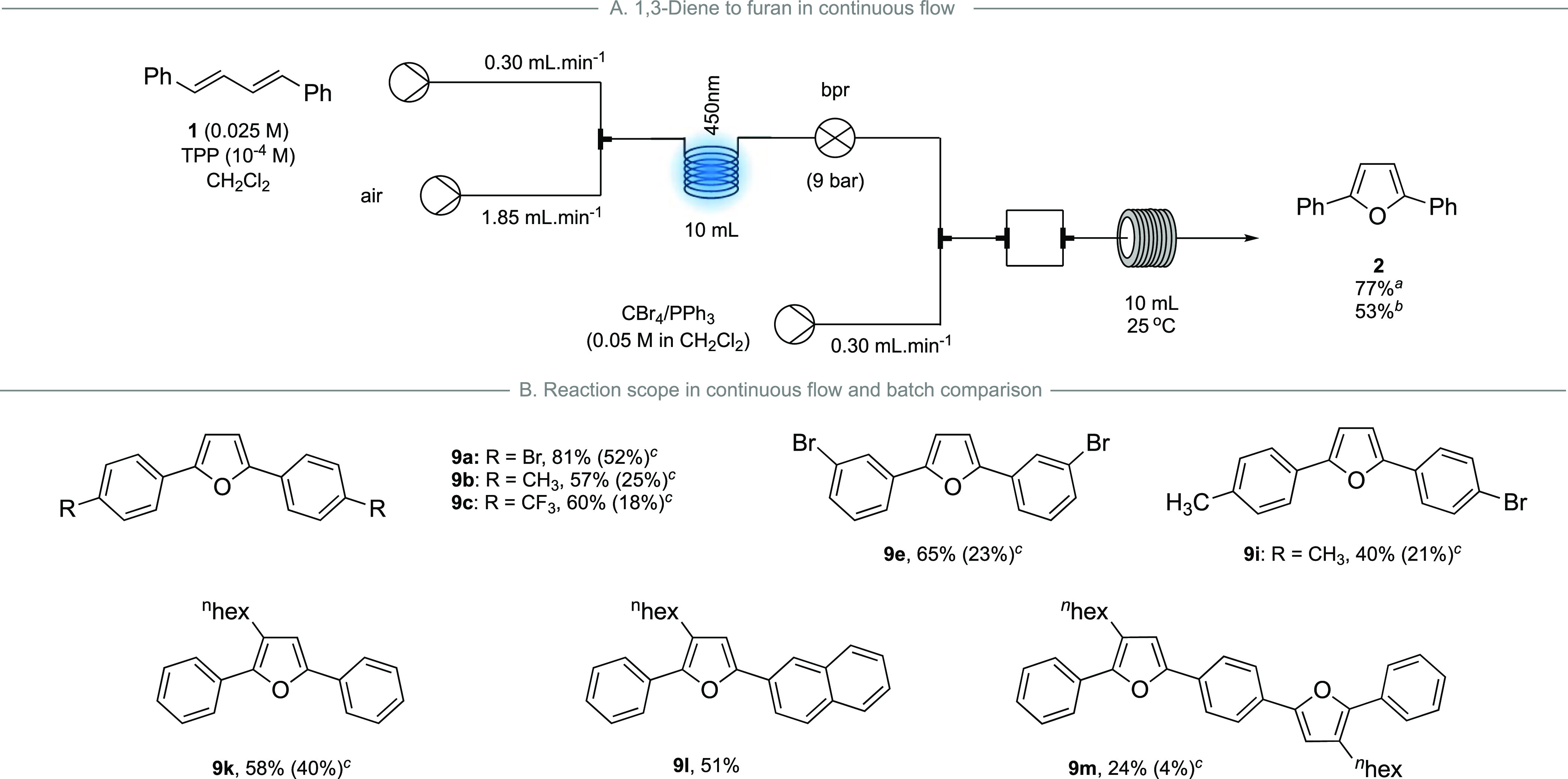
(a) Telescope Continuous-Flow Setup for the Synthesis of 2,5-Phenylfuran **2** and (B) Reaction Scope for the Synthesis of 2,5-Diarylfurans 1 mmol scale; ^b^10 mmol scale; ^c^calculated yield for the two-step batch process.

With optimization of each step completed, we then investigated conversion of **1** to **2**. The setup for the combined process saw the Appel reagent being introduced to the stream of newly generated endoperoxide after the UV-150 reactor coil (Scheme 7A). The BPR (9 bar) is placed after the first reactor coil and before the introduction of the Appel reagent; therefore, there is a step down in pressure which permitted the compressed gas to expand, minimizing the amount of dissolved oxygen in the solvent. We anticipated that this would minimize the quantity of oxygen in the system and result in minimal quenching of the active P(V) reagent in the dehydration step.

The conditions for the telescoped process consisted of a solution of **1** in CH_2_Cl_2_ (0.025 M) containing TPP (10^–4^ M) pumped at 0.30 mL min^–1^ and combined with air pumped at 1.85 mL min^–1^ under 9 bar pressure. After passing through a UV-150 reactor coil equipped with a 450 nm lamp, a step down to atmospheric pressure occurred and then freshly generated Appel reagent (0.05 M in CH_2_Cl_2_) was introduced at 0.30 mL min^–1^. The pressure step down resulted in a large expansion of dissolved gas in the tubing and so, to ensure adequate mixing of the generated endoperoxide and introduced Appel reagent, the reaction mixture was split and recombined before being passed through a 10 mL reactor coil held at 25 °C and the output was collected. This provided furan **2** in a good yield of 77%, without the need for purification of the intermediate endoperoxide in one multistep continuous-flow procedure. To ensure the scalability of the reaction procedure, this setup was performed on a 10.00 mmol scale of **1** giving furan **2** in an acceptable 53% yield.

Finally, we used the continuous-flow setup on several 1,3-diene substrates to ascertain substrate scope and to compare with the sequential batch conditions from Scheme 3 (Scheme 7B). The symmetrical 4-bromophenyl, 4-methylphenyl, and 4-trifluoromethylphenyl 1,3-dienes (**7a**–**c**) were transformed into their, respective, furans (**9a**–**c**), as was the symmetrical 3-bromophenyl diene **9e**, all in good yields. Importantly, the yields were significantly higher than the sequential batch approach. The unsymmetrical furan **9i**, whose yield was 21% using the sequential batch chemistry, could be obtained in a much improved 40% yield. The 3-hexyl 2,5-diphenylfuran **9k** synthesis performed better in continuous flow, being isolated in 58%, as did the naphthyl analogue **9l**. Finally, the furan-phenylene **9m**, previously obtained in only 4% yield using the sequential batch process, was isolated in 24% yield using the continuous-flow setup. It is likely that the observed improvements in yield are due to the increased efficiencies of photochemical reactions in continuous flow and telescoping the sequence thereby eliminating isolation of the intermediate endoperoxides.

### Optoelectronic Properties

**Figure 2:**
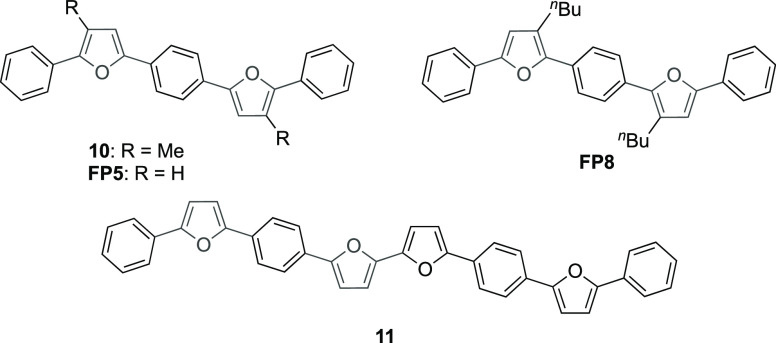
Current short-chain linear-conjugated furan oligomers.

A small number of homologues and regioisomeric homologues of **9m** have previously been reported which provided motivation to gain insights into the optical and electrochemical properties of this molecule (Figure 2).

The performance of **10**, the methyl-substituted homologue of **9m**, was presented in an early contribution concerning blue organic light-emitting diodes (OLEDs)^16a^ although no synthetic details were provided and, in any case, the molecule formed poor-quality films and therefore inefficient devices. The nonalkylated derivative **FP5** was first synthesized via Negishi coupling in 1987^16b^ and, more recently, has been resynthesized using a combination of traditional palladium-mediated Stille and Suzuki cross-coupling approaches to study its luminescent properties and its successful application in organic electronic devices.^16c−16f^ Common to the synthesis of **FP5** (and derivatives thereof) in all of these papers is the use of metal catalysts and/or organometallic reagents. We reiterate that our method circumvents the use of toxic or precious metals and facilitates the formation of only a single monodisperse product. This is important to highlight as during the study of **FP5** and related thiophene-phenylene co-oligomers, it became apparent that some key device characteristics of **FP5** were being modulated by interplay with trace quantities, estimated at little as <0.002%, of **FP8** which had formed as a homocoupling byproduct in the final stage of the synthesis.^16f^ While traces of **FP8** actually had a beneficial effect on the performance of **FP5** from a material standpoint, the ability to obtain single products cleanly and reliably is a central tenet of organic synthetic chemistry which is facilitated by the continuous-flow methodology presented here.

The only analogue of **9m** we are aware of that has been synthesized without relying on metal-catalyzed cross-coupling is the isomeric homologue **FP8** featuring *n*-butyl chains on 3,3′- rather than 4,4′-positions of the furan rings, which was elegantly synthesized using propargylic dithioacetals in conjunction with an organocuprate.^16g^

Compounds **2**, **9b**, **9c**, **9k**, **9l**, and **9m** were selected as a good series of compounds to study and identify any structure–property relationships arising from (1) inductive effects of the terminal end-groups; (2) the presence of the *n*-hexyl chains; and (3) increasing the effective conjugation length of the molecules. UV/vis absorbance and fluorescence spectroscopy were performed for these compounds. The onset of the lowest-energy absorbance was used to calculate the optical HOMO–LUMO gap (*E*_g_^opt^) of the compounds. DFT (B3LYP/def2-TZVP)^17a,17b^ and TDDFT calculations were also completed using ORCA v.4.0.1.2 and v.5.0.3.^18a,18b^ to assist interpretation of our experimental results where necessary. Ground-state geometries were optimized, and Frontier orbital energies and distributions were calculated. Calculated HOMO and LUMO energies and the calculated HOMO–LUMO gap (*E*_g_^calc^) are shown alongside the optical data in Table 2. Unless otherwise shown, ground-state geometries and Frontier orbital plots for all molecules can be found in the Supporting Information (Figure S1). Gratifying, the overall trends in both the experimental and theoretical results agree very well.

**Table 2:** Absorbance, Emission, and Calculated Frontier Orbital and Structural Properties for Compounds **2**, **9b**, **9c**, and **9k**–**m**

<!—Col Count:8F0E0	λ_abs_ (nm)	λ_em_ (nm)	*E*_g_^opt^ (eV)^a^	HOMO (eV)	LUMO (eV)	*E*_g_^calc^ (eV)	τ (deg)
**2**	318 (sh), 327, 342	355, 370, 384, 412 (sh)	3.41	–5.408	–1.484	3.92	0.2
**9b**	321 (sh), 331, 347	358, 376, 390, 417 (sh)	3.37	–5.207	–1.329	3.88	0.1
**9c**	326 (sh), 334, 351	364, 381, 397, 425 (sh)	3.33	–6.040	–2.237	3.80	0.2
**9k**	318	359, 377, 394, 421 (sh)	3.40	–5.367	–1.299	4.07	33.2, 4.2
**9l**	280, 290, 339, 364 (sh)	406	3.10	–5.273	–1.575	3.70	–26.3, −3.0
**9m**	305, 363, 377, 395	421, 444, 469, 506	2.81	–5.022	–1.689	3.33	26.5, 1.7

a *E*_g_^opt^ = 1240.68/λ_onset_.

**Figure 3:**
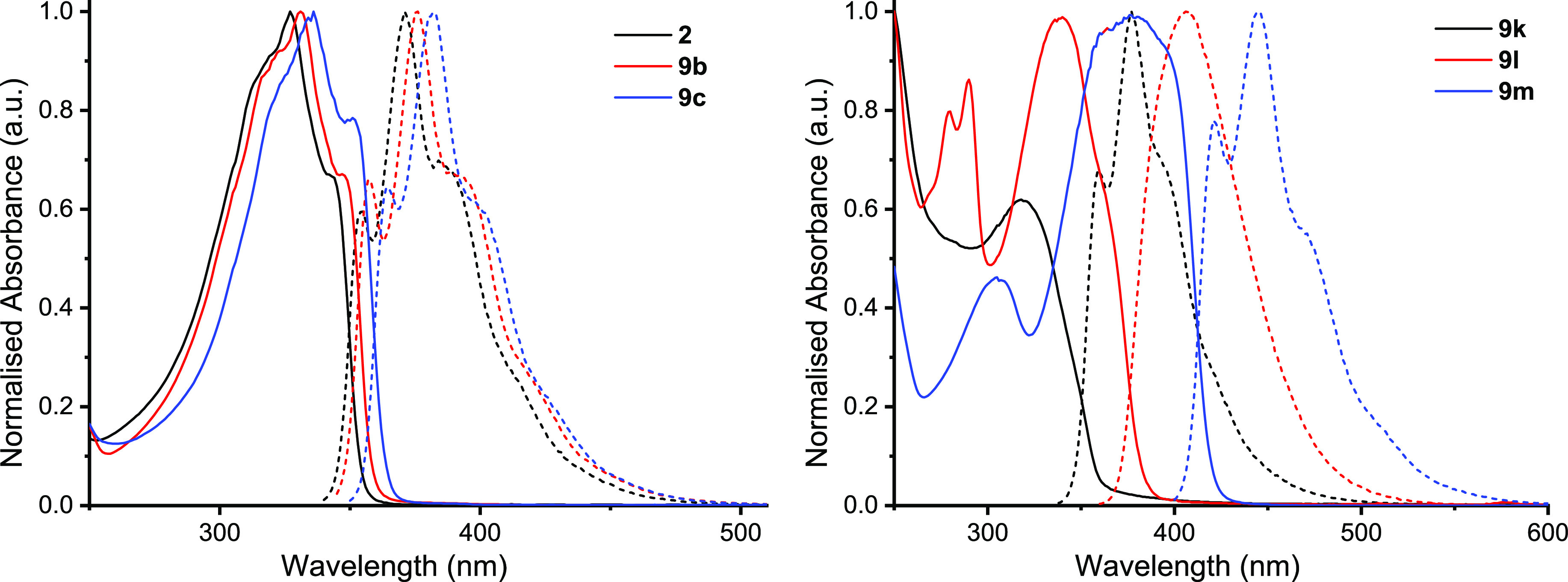
Absorbance and emission spectra of **2**, **9b**, **9c**, **9k**, **9l**, and **9m**.

The absorbance and emission spectra for **2**, **9b**, and **9c** (Figure 3) have essentially identical band shapes with clear vibronic structure evident in both which is in good agreement with previous reports for these molecules and related trimers.^3a^ Oligo furans and furan containing co-oligomers such as **FP5** are known to be significantly more rigid than their thiophene counterparts, which gives rise to the increased vibronic structure in their spectra.^3h,13a,13b,19a,19b^ Indeed, the computationally optimized geometries for these compounds are entirely coplanar with dihedral angles τ between the terminal phenyl and furan rings of τ = ∼0° in all cases. The emission spectra mirror the absorbance spectra closely which implies that there is no significant change in molecular conformation or dipole upon excitation. Both **9b** and **9c** are slightly red-shifted in comparison with **2** and therefore have a reduced *E*_g_^opt^. The DFT results confirmed that the redshift in absorbance for **9b** is due to the inductive electron-donating effect of the methyl groups which destabilizes the HOMO, while in **9c**, the strongly electron-withdrawing inductive effect of the CF_3_ groups serves to significantly stabilize both the HOMO and the LUMO.

The *n*-hexyl-bearing compounds **9k–m** demonstrate distinct differences from the other compounds. The only structural difference between **2** and **9k** is the presence of a hexyl chain on the outermost β-position of furan whose calculations indicate enforcing a twist τ = 26° between the furan and the phenyl rings adjacent to the hexyl chains. This causes stark differences between the spectra for the molecules; the fine structure observed in the absorbance spectrum for **2** has vanished but returns in the emission spectrum albeit slightly red-shifted and appearing almost identical to that of **9b**. We suggest this signifies that steric incumbency of the hexyl chain forces the ground-state molecule to adopt a less-rigid noncoplanar configuration; therefore, the fine structure of the absorbance spectrum has been lost. Upon excitation, the conjugated backbone of **9k** assumes the same coplanar conformation as for **9b** resulting in the vibronic fine structure of the emission band being re-established. While a decrease in effective conjugation length might be expected to lead to a wider *E*_g_, the electron-donating effect of the hexyl chain has raised the HOMO energy sufficiently to compensate for this.

**Figure 4:**
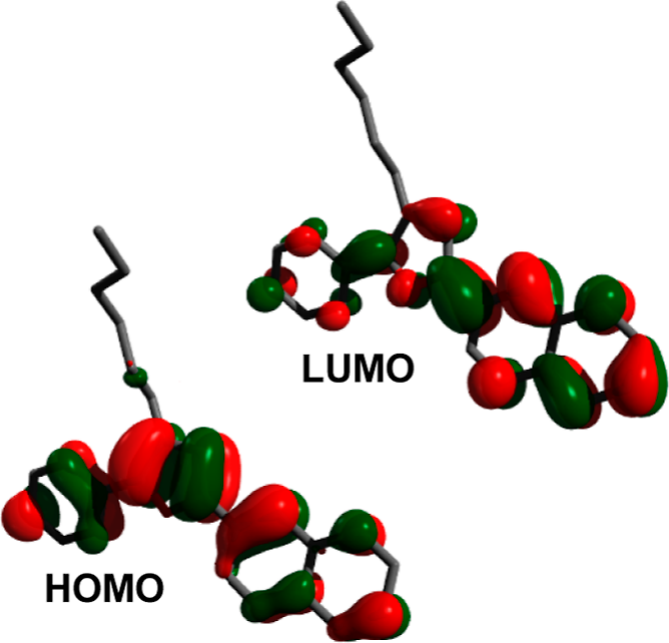
HOMO and LUMO of **9l**.

For **9l**, the absence of any significant fine structure in either the absorbance or emission spectra alongside a notable redshift in the profile of both the absorbance and emission is indicative of a transition with some dipolar character. This is corroborated by the computational results (Figure 4) which show the HOMO residing largely over the phenyl and furan rings, while the LUMO is sequestered more over the naphthalene moiety.

Finally, considering **9m**, the absorbance profile for the main band is very broad with two clear shoulders which are of almost equal intensity to the central λ_max_ at 377 nm. This molecule has the narrowest *E*_g_^opt^ and highest HOMO energy due to its longer conjugation length and the electron-donating effect of the *n*-hexyl chains. The emission spectrum for **9m** is only red-shifted by 7 nm in comparison with its near-isomer **11**.^16g^ The emission profile of **9m** displayed fine structure in a similar fashion to **9k** which indicates that the excited state assumes a coplanar geometry. To support this interpretation, TDDFT (CAM-B3LPY/6-31G**)^20^ was employed to optimize the geometry of the ground and first-excited singlet state of **9m** (Figure S2) which did indeed predict a planar structure in agreement with our key interpretations. For the excited-state calculation, *n*-propyl rather than *n*-hexyl chains was employed to reduce the computational cost.

**Figure 5:**
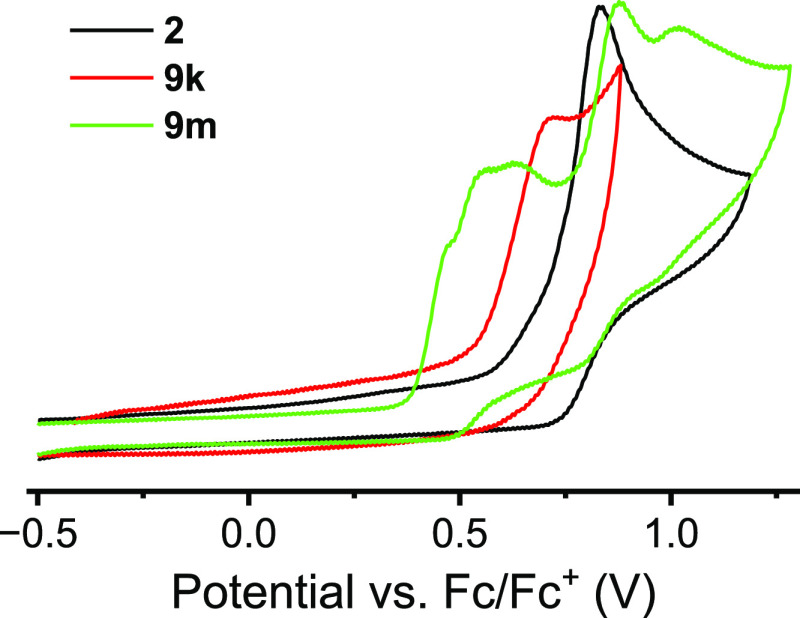
Cyclic voltammograms for compounds **2**, **9k**, and **9m**. Cyclic voltammogram plotted according to IUPAC convention.

Finally, cyclic voltammetry (CV) was performed on compounds **2**, **9k**, and **9m** to identify any changes in electrochemical properties arising from the twists induced by the hexyl chain(s) and to see how the longer effective conjugation length of **9m** impacts upon the number and reversibility of any redox events (Figure 5 and Table 3). All three compounds displayed only irreversible oxidation waves. **2** and **9k** had one oxidation peak, while the longer co-oligomer **9m** resulted in four peaks in the voltammogram. Estimates for the HOMO energy of these compounds were obtained using the onset of the lowest-energy oxidation wave, and the optical HOMO–LUMO gap was used to obtain an estimate of the LUMO energies of the compounds. **9k** has a lower first oxidation potential and a slightly shallower HOMO energy in comparison with **2**. In agreement with the computational results, this highlights again that for the short trimers, the electron-donating influence of the *n*-hexyl chain more than counterbalances the effect on the HOMO of any disturbance in the effective conjugation length of the molecule arising from steric hindrance on the trimer. For **9m**, the longer chain length and the presence of two *n*-hexyl chains result in a much more significant increase in the HOMO energy and a lower first oxidation peak potential.

**Table 3:** Electrochemical Properties of **2**, **9k**, and **9m**

	*E*^ox^ (V)^a^	*E*^ox2^ (V)^a^	*E*^red2^ (V)^a^	*E*^red2^ (V)^a^	HOMO (eV)^b^	LUMO (eV)^c^
**2**	+0.83^ir^				–5.64	–2.23
**9k**	+0.72^ir^				–5.69	–2.29
**9m**	+0.48^ir^	+0.57^ir^	+0.63^ir^	+0.88^ir^	–5.47	–2.66

a CV data were obtained from solutions of ca. 0.1 mmol analyte, 0.1 M *n*-Bu_4_NPF_6_ in CH_3_CN at RT. A 3 mm glassy carbon disc working electrode, nonaqueous Ag/AgNO_3_ reference electrode, and a Pt wire counter electrode were used. Scans began at +0.00 V and were first swept to anodic potentials at a scan rate of 100 mV/s. Potentials are quoted versus Fc/Fc^+^ couple which was used as the internal reference.

b *E*_HOMO_ = −(*E*_onset,ox_ vs Fc/Fc^+^ + 5.10) eV.^21^

c *E*_LUMO_ = *E*_HOMO_ + *E*_g_^opt^. ^ir^ Irreversible peak.

## Conclusions

In summary, we have used ^1^O_2_ oxidation and Appel reagent-mediated dehydration conditions and established a robust metal-free synthesis of 2,5-diarylfurans from 1,3-diene precursors.^22^ Performing this sequence in traditional batch conditions required isolation of the intermediate endoperoxide. Subsequent dehydration of each endoperoxide afforded a broad range of medicinally important 2,5-diarylfuran precursors, containing synthetic handles for post functionalization. Additionally, this sequence provided several 2,5-diarylfurans with applicability in material chemistry and was further exemplified by a transition-metal-free synthesis of a novel furan-phenylene co-oligomer. A short study of the absorbance and emission spectroscopy and the CV of a selection of these new compounds was completed to establish structure–property relationships with interpretation supported by calculations, adding to the comparatively small amount of literature concerning furan-based π-functional materials thereby providing some direction to those working in this area. Furthermore, this methodology presents an opportunity to investigate the thiophene equivalents,^23^ which are currently synthesized through transition-metal cross coupling methods. By utilizing flow technology, the batch sequence was translated into one continuous process, bypassing the isolation of the challenging endoperoxide. This was achieved by careful matching of flow rates of the oxidation and Appel dehydration steps and notably using air instead of O_2_ in the crucial photooxidation step. Additionally, to achieve optimum flow conditions, a BPR at 9 bar was required after the first photoreactor, with a subsequent step down in pressure prior to the introduction of the Appel reagent. Finally, this work offers a technically simple and streamlined metal-free method to directly convert 1,3-dienes into 2,5-diarylfurans, thereby reducing waste, increasing safety, and demonstrating the utility of continuous-flow technology.

## Experimental Sections

### Batch Photooxidation—Dehydration General Procedure

A solution of diene (1.00 mmol) in 19:1 CH_2_Cl_2_/MeOH (30 mL) containing 10^–4^ M disodium rose Bengal was irradiated for 5 days at room temperature with a 400 W halogen light source (at a distance of approximately 20 cm), while a constant stream of oxygen was passed through the solution.^11c,15^ After this period, solvents were removed in vacuo, and the residue was purified by column chromatography. The stability of the endoperoxide proved capricious, so yield and product purity were determined through ^1^H and ^13^C NMR analysis and then used directly in the dehydration step. To a solution of CBr_4_ (1.10 equiv) in CH_2_Cl_2_ cooled to 0 °C was added PPh_3_ (1.10 equiv), and the resulting mixture was stirred for 20 min. After this period, a solution of endoperoxide in CH_2_Cl_2_ was added, and the solution was brought to room temperature and stirred for 16 h. Solvents were removed in vacuo, and the residue was purified by column chromatography to yield the product.

Using this procedure, the following substrates were synthesized:

#### 2,5-Diphenylfuran **2**(11c)

Using the batch general procedure, commercially available *trans*,*trans*-1,4-diphenyl-1,3-butadiene (**1**) (412 mg, 2.00 mmol) and rose Bengal disodium salt (6 mg, 0.005 mmol) in 19:1 CH_2_Cl_2_/MeOH (60 mL) provided the intermediate endoperoxide, 3,6-diphenyl-3,6-dihydro-1,2-dioxine (**3**) as a colorless solid after column chromatography (308 mg, 1.30 mmol, 65%); *R*_*f*_ = 0.15 (9/1 hexanes/ethyl acetate); ^1^H NMR (CDCl_3_, 500 MHz): δ 7.77 (d, 4H, *J* = 8.0 Hz), 7.43 (t, 4H, *J* = 7.5 Hz), 7.29 (t, 2H, *J* = 7.5 Hz), 6.75 (s, 2H); ^13^C {^1^H} NMR (CDCl_3_, 126 MHz): δ 137.7, 128.8, 128.7, 128.5, 127.5, 80.3. Using the general batch procedure and **3** (120 mg, 0.50 mmol), PPh_3_ (144 mg, 0.55 mmol), and CBr_4_ (182 mg, 0.55 mmol), the title compound **2** was obtained as a colorless solid after column chromatography (112 mg, 0.50 mmol, 99%); *R*_*f*_ = 0.40 (9/1 hexanes/ethyl acetate); ^1^H NMR (CDCl_3_, 500 MHz): δ 7.77 (d, 4H, *J* = 7.3 Hz), 7.43 (t, 4H, *J* = 7.5 Hz), 7.29 (t, 2H, *J* = 7.5 Hz), 6.76 (s, 2H); ^13^C {^1^H} NMR (CDCl_3_, 126 MHz): δ 153.4, 130.9, 128.8, 127.4, 123.8, 107.3; IR (ν_max_, cm^–1^) 2980, 1662, 1600, 1587, 1508, 1479, 1447, 1386.

#### 2,5-Bis(4-bromophenyl)furan **9a**(24a)

Using the batch general procedure and (1*E*,3*E*)-1,4-bis(4-bromophenyl)buta-1,3-diene (**7a**) (364 mg, 1.00 mmol), rose Bengal disodium salt (3 mg, 0.005 mmol) in 19:1 CH_2_Cl_2_/MeOH (30 mL) provided the intermediate endoperoxide, 3,6-bis-(4-bromophenyl)-3,6-dihydro-6-phenyl-1,2-dioxine (**8a**), as a colorless solid after column chromatography (235 mg, 0.60 mmol, 60%); *R*_*f*_ = 0.27 (9/1 hexanes/ethyl acetate). ^1^H NMR (CDCl_3_, 500 MHz): δ 7.50 (dt, 4H, *J* = 8.7, 2.1 Hz), 7.28 (dd, 4H, *J* = 6.6, 1.9 Hz), 6.29 (d, 2H, *J* = 0.6 Hz), 5.58 (s, 2H); ^13^C {^1^H} NMR (CDCl_3_, 126 MHz): δ 136.7, 133.3, 130.3, 129.7, 127.6, 127.1, 124.4, 79.0. Using the general batch procedure and **8a** (106 mg, 0.27 mmol), PPh_3_ (78 mg, 0.30 mmol), and CBr_4_ (99 mg, 0.30 mmol), the title compound **9a** was obtained as a colorless solid after column chromatography (88 mg, 0.24 mmol, 87%); *R*_*f*_ = 0.35 (19/1 hexanes/ethyl acetate); ^1^H NMR (CDCl_3_, 400 MHz): δ 7.61–7.59 (m, 4H), 7.55–7.52 (m, 4H), 6.74 (s, 2H); ^13^C {^1^H} NMR (CDCl_3_, 100 MHz): δ 152.7, 132.0, 129.5, 127.9, 125.3, 121.4, 108.0; IR (ν_max_, cm^–1^): 2980, 1578, 1472, 1407, 1346; HRMS (ESI) *m*/*z*: [M + K]^+^ calcd for C_16_H_12_Br_2_O_2_K, 432.8841; found, 432.8868.

#### 2,5-Di-*p*-tolylfuran **9b**(24a)

Using the batch general procedure and (1*E*,3*E*)-1,4-di-*p*-tolylbuta-1,3-diene (**7b**) (234 mg, 1.00 mmol), rose Bengal disodium salt (3 mg, 0.005 mmol) in 19:1 CH_2_Cl_2_/MeOH (30 mL) provided the intermediate endoperoxide, 3,6-bis-(4-methylphenyl)-3,6-dihydro-6-phenyl-1,2-dioxine (**8b**) as a colorless solid after column chromatography (124 mg, 0.47 mmol, 47%). *R*_*f*_ = 0.27 (9/1 hexanes/ethyl acetate); ^1^H NMR (CDCl_3_, 500 MHz): δ 7.33 (d, 4H, *J* = 7.9 Hz), 7.18 (d, 4H, *J* = 7.9 Hz), 6.29 (s, 2H), 5.59 (s, 2H), 2.35 (s, 6H); ^13^C {^1^H} NMR (CDCl_3_, 126 MHz): δ 138.7, 134.8, 129.3, 128.6, 127.5, 80.1, 21.3. Using the general batch procedure and **8b** (123 mg, 0.46 mmol), the title compound **9b**, PPh_3_ (132 mg, 0.51 mmol), and CBr_4_ (168 mg, 0.51 mmol) were obtained as a colorless solid after column chromatography (60 mg, 0.18 mmol, 53%). *R*_*f*_ = 0.62 (9/1 hexanes/ethyl acetate); ^1^H NMR (CDCl_3_, 500 MHz): δ 7.67–7.65 (m, 4H), 6.95–6.93 (m, 4H), 6.58 (s, 2H), 3.85 (s, 6H); ^13^C {^1^H} NMR (CDCl_3_, 126 MHz): δ 159.0, 152.9, 125.1, 124.2, 114.2, 105.7, 55.4; IR (ν_max_, cm^–1^): 2963, 2841, 1610, 1588, 1490, 1467, 1440, 1413, 1301; HRMS (ESI) *m*/*z*: [M + H]^+^ calcd for C_18_H_16_O_3_, 280.1094; found, 280.1093.

#### 2,5-Bis(4-(trifluoromethyl)phenyl)furan **9c**(24b)

Using the batch general procedure and (1*E*,3*E*)-1,4-bis(4-(trifluoromethyl)phenyl)buta-1,3-diene (**7c**) (342 mg, 1.00 mmol), rose Bengal disodium salt (3 mg, 0.005 mmol) in 19:1 CH_2_Cl_2_/MeOH (30 mL) provided the intermediate endoperoxide, 3,6-bis-(4-trifluoromethylphenyl)-3,6-dihydro-6-phenyl-1,2-dioxine (**8c**), as a colorless solid after column chromatography (114 mg, 0.31 mmol, 31%). *R*_*f*_ = 0.16 (9/1 hexanes/ethyl acetate); ^1^H NMR (CDCl_3_, 500 MHz): δ 7.64 (d, 2H, *J* = 8.2 Hz), 7.53 (d, 2H, *J* = 8.2 Hz), 6.36 (d, 1H, *J* = 0.9 Hz), 5.71 (s, 1H); ^13^C {^1^H} NMR (CDCl_3_, 126 MHz): δ 141.3, 131.2, 130.9, 128.5, 127.3, 125.8, 125.7, 125.1, 122.9, 79.5. Using the general batch procedure and **9b** (114 mg, 0.32 mmol), PPh_3_ (95 mg, 0.36 mmol), and CBr_4_ (120 mg, 0.36 mmol), the title compound **9c** was obtained as a colorless solid after column chromatography (66 mg, 0.18 mmol, 58%). *R*_*f*_ = 0.47 (9/1 hexanes/ethyl acetate; ^1^H NMR (CDCl_3_, 500 MHz): δ 7.84 (d, 4H, *J* = 8.2 Hz), 7.67 (d, 2H, *J* = 8.2 Hz), 6.88 (s, 2H); ^13^C {^1^H} NMR (CDCl_3_, 126 MHz): δ 152.9, 133.5, 129.5 (q, *J* = 32.6 Hz), 125.9 (q, *J* = 4.3 Hz), 124.1 (q, *J* = 272.8 Hz), 124.0, 109.4; IR (ν_max_, cm^–1^): 1616, 1424, 1318; MS (ESI) *m*/*z*: 395.1 [M + K]^+^; no molecular ion was detected by electrospray ionization–high-resolution mass spectroscopy (ESI-HRMS). Crystals suitable for single-crystal X-ray analysis of **9c** were obtained by slow evaporation of a CDCl_3_ solution and confirmed the structure.

#### 2,5-Bis-(4-methoxyphenyl)furan **9d**(24a)

Using the batch general procedure (1*E*,3E)-1,4-bis(4-(methoxy)phenyl)buta-1,3-diene (**7d**) (298 mg, 1.00 mmol), rose Bengal disodium salt (3 mg, 0.005 mmol) in 19:1 CH_2_Cl_2_/MeOH (30 mL) provided the intermediate endoperoxide, 3,6-bis(4-methoxyphenyl)-3,6-dihydro-6-phenyl-1,2-dioxine (**8d**) as a colorless solid after column chromatography (63 mg, 0.21 mmol, 21%). *R*_*f*_ = 0.15 (4/1 hexanes/ethyl acetate); ^1^H NMR (CDCl_3_, 500 MHz): δ 7.38–7.36 (m, 4H), 6.91–6.89 (m, 4H), 6.29 (s, 2H), 5.57 (s, 2H), 3.81 (s, 6H); ^13^C {^1^H} NMR (CDCl_3_, 126 MHz): δ 160.1, 130.1, 127.6, 114.0, 79.8, 55.4. Using the general batch procedure and **7d** (63 mg, 0.21 mmol), PPh_3_ (63 mg, 0.24 mmol), and CBr_4_ (80 mg, 0.24 mmol), the title compound **9d** was obtained as a colorless solid after column chromatography (12 mg, 0.04 mmol, 21%). *R*_*f*_ = 0.23 (9/1 hexanes/ethyl acetate); ^1^H NMR (CDCl_3_, 500 MHz): δ 7.67–7.65 (m, 4H), 6.95–6.93 (m, 4H), 6.58 (s, 2H), 3.85 (s, 6H); ^13^C {^1^H} NMR (CDCl_3_, 126 MHz): δ 159.0, 152.9, 125.1, 124.2, 114.2, 105.7, 55.4; IR (ν_max_, cm^–1^): 2963, 2841, 1610, 1588, 1490, 1467, 1440, 1413, 1301; HRMS (ESI) *m*/*z*: [M + H]^+^ calcd for C_18_H_16_O_3_, 280.1094; found, 280.1093.

#### 2,5-Bis(3-bromophenyl)furan **9e**(24c)

Using the batch general procedure, (1*E*,3*E*)-1,4-bis(3-bromophenyl)buta-1,3-diene (**7e**) (364 mg, 1.00 mmol) and rose Bengal disodium salt (3 mg, 0.005 mmol) in 19:1 CH_2_Cl_2_/MeOH (30 mL) provided the intermediate endoperoxide, 3,6-bis(3-bromophenyl)-3,6-dihydro-6-phenyl-1,2-dioxine (**8e**), as a colorless solid after column chromatography (106 mg, 0.27 mmol, 27%). *R*_*f*_ = 0.14 (19/1 hexanes/ethyl acetate); ^1^H NMR (CDCl_3_, 500 MHz): δ 7.56 (t, 2H, *J* = 1.5 Hz), 7.50–7.48 (m, 2H), 7.38–7.34 (m, 2H), 7.26 (t, 2H, *J* = 8.5 Hz, 15.5 Hz), 6.31 (s, 2H), 5.59 (s, 2H); ^13^C {^1^H} NMR (CDCl_3_, 126 MHz): δ 136.7, 133.3, 130.3, 129.6, 127.6, 127.1, 124.4, 79.0. Using the general batch procedure and **8e** (106 mg, 0.27 mmol), PPh_3_ (79 mg, 0.30 mmol), and CBr_4_ (100 mg, 0.30 mmol), the title compound **9e** was obtained as a colorless solid after column chromatography (88 mg, 0.24 mmol, 87%). *R*_*f*_ = 0.35 (19/1 hexanes/ethyl acetate); ^1^H NMR (CDCl_3_, 500 MHz): δ 7.87 (t, 2H, *J* = 2.0 Hz), 7.69–7.61 (m, 2H), 7.45–7.37 (m, 2H), 7.28 (t, 2H, *J* = 7.5 Hz), 6.76 (s, 2H); ^13^C {^1^H} NMR (CDCl_3_, 126 MHz): δ 152.4, 132.4, 130.5, 130.4, 126.7, 123.1, 122.4, 108.5; IR (ν_max_, cm^–1^): 1602, 1581, 1560, 1526, 1465, 1408, 1327; HRMS (ESI) *m*/*z*: [M + K]^+^ calcd for C_16_H_12_Br_2_O_2_K, 432.8841; found, 432.8868.

#### 2,5-Bis(2-bromophenyl)furan **9f**

Using the batch general procedure and (1*E*,3*E*)-1,4-bis(2-bromophenyl)buta-1,3-diene (**7f**) (364 mg, 1.00 mmol), rose Bengal disodium salt (3 mg, 0.005 mmol) in 19:1 CH_2_Cl_2_/MeOH (30 mL) provided the intermediate endoperoxide, 3,6-bis(2-bromophenyl)-3,6-dihydro-6-phenyl-1,2-dioxine (**8f**), as a colorless solid after column chromatography (125 mg, 0.32 mmol, 32%). *R*_*f*_ = 0.25 (19/1 hexanes/ethyl acetate); ^1^H NMR (CDCl_3_, 500 MHz): δ 7.61 (dd, 2H, *J* = 8.1, 1.1 Hz, 7.51 (dd, 2H, *J* = 7.9, 1.6 Hz), 7.32 (td, 2H, *J* = 7.6, 1.2 Hz), 7.21 (td, 2H, *J* = 7.7, 1.7 Hz), 6.35 (s, 2H), 6.08 (s, 2H); ^13^C {^1^H} NMR (CDCl_3_, 126 MHz): δ 136.7, 133.3, 130.3, 129.6, 127.6, 127.1, 124.4, 79.0. Using the general batch procedure and **8f** (125 mg, 0.32 mmol), PPh_3_ (95 mg, 0.36 mmol), and CBr_4_ (120 mg, 0.36 mmol), the title compound **9f** was obtained as a colorless solid after column chromatography (10 mg, 0.03 mmol, 9%). *R*_*f*_ = 0.57 (9/1 hexanes/ethyl acetate); ^1^H NMR (CDCl_3_, 500 MHz): δ 7.90 (dd, 2H, *J* = 7.8, 1.6 Hz), 7.68 (dd, 2H, *J* = 7.8, 1.2 Hz), 7.39 (td, 2H, *J* = 7.6, 1.1 Hz), 7.30 (s, 2H), 7.15 (td, 2H, *J* = 7.6, 1.6 Hz); ^13^C {^1^H} NMR (CDCl_3_, 126 MHz): δ 151.0, 134.3, 131.0, 128.9, 128.7, 127.5, 119.7, 112.5, 29.8; IR (ν_max_, cm^–1^): 2926, 1678, 1596, 1566, 1502, 1485, 1458, 1421; HRMS (ESI) *m*/*z*: [M + K]^+^ calcd for C_16_H_12_Br_2_O_2_K, 432.8841; found, 432.8868.

#### 2-(4-Bromophenyl)-5-(4-chlorophenyl)furan **9h**(24d)

Using the batch general procedure and 1-bromo-4-[(1*E*,3*E*)-4-(4-chlorophenyl)-1,3-butadien-1-yl]benzene (**7h**) (319 mg, 1.00 mmol), rose Bengal disodium salt (3 mg, 0.005 mmol) in 19:1 CH_2_Cl_2_/MeOH (30 mL) provided the intermediate endoperoxide, 3-(4-bromophenyl)-6-(4-chlorophenyl)-3,6-dihydro-6-phenyl-1,2-dioxine (**8h**), as a colorless solid after column chromatography (42 mg, 0.12 mmol, 12%). *R*_*f*_ = 0.18 (19/1 hexanes/ethyl acetate); ^1^H NMR (CDCl_3_, 500 MHz): δ 7.51 (dd, 2H, *J* = 6.6, 1.6 Hz), 7.40–7.26 (m, 6H), 6.29 (s, 2H), 5.59 (d, 2H, *J* = 6.0 Hz); ^13^C {^1^H} NMR (CDCl_3_, 126 MHz): δ 136.6, 135.9, 134.9, 131.9, 130.9, 130.0, 129.8, 128.9, 127.4, 127.3, 123.1, 79.5. Using the general batch procedure and **8h** (42 mg, 0.12 mmol), PPh_3_ (37 mg, 0.14 mmol), and CBr_4_ (47 mg, 0.14 mmol), the title compound **9h** was obtained as a colorless solid after column chromatography (35 mg, 0.10 mmol, 88%). *R*_*f*_ = 0.31 (19/1 hexanes/ethyl acetate); ^1^H NMR (CDCl_3_, 500 MHz): δ 7.67–7.64 (m, 2H), 7.61–7.58 (m, 2H), 7.54–7.53 (m, 2H), 7.39–7.36 (m, 2H), 6.74 (d, 1H, *J* = 4 Hz), 6.72 (d, 2H, *J* = 4 Hz); ^13^C {^1^H} NMR (CDCl_3_, 126 MHz): δ 152.7, 152.6, 133.3, 132.0, 129.5, 129.1, 129.1, 125.3, 125.0, 121.3, 107.9, 107.9; IR (ν_max_, cm^–1^): 2921, 2285, 1719, 1618, 1559, 1473, 1409; HRMS (ESI) *m*/*z*: [M + K]^+^ calcd for C_16_H_10_BrClOK, 370.9235; found, 370.9235.

#### 2-(4-Bromophenyl)-5-(4-methylphenyl)furan **9i**(24e)

Using the batch general procedure and 1-bromo-4-[(1*E*,3*E*)-4-(4-methylphenyl)-1,3-butadien-1-yl]benzene (**7i**) (299 mg, 1.00 mmol), rose Bengal disodium salt (3 mg, 0.005 mmol) in 19:1 CH_2_Cl_2_/MeOH (30 mL) provided the intermediate endoperoxide, 3-(4-methylphenyl)-6-(4-bromophenyl)-3,6-dihydro-6-phenyl-1,2-dioxine (**8i**), as a colorless solid after column chromatography (112 mg, 0.34 mmol, 34%). *R*_*f*_ = 0.23 (9/1 hexanes/ethyl acetate); ^1^H NMR (CDCl_3_, 500 MHz): δ 7.52–7.49 (m, 2H), 7.36–7.31 (m, 2H), 7.30–7.28 (m, 2H), 7.18 (d, 2H, *J* = 8 Hz), 6.35–6.22 (m, 2H), 5.64 (d, 1H, *J* = 1.9 Hz), 5.54 (d, 1H, *J* = 1.6 Hz), 2.35 (s, 3H); ^13^C {^1^H} NMR (CDCl_3_, 126 MHz): δ 139.0, 137.2, 134.1, 131.8, 130.1, 129.4, 128.5, 128.2, 126.7, 122.8, 80.2, 79.4, 21.3. Using the general batch procedure and **8i** (112 mg, 0.34 mmol), PPh_3_ (100 mg, 0.38 mmol), and CBr_4_ (125 mg, 0.38 mmol), the title compound **9i** was obtained as a colorless solid after column chromatography (65 mg, 0.21 mmol, 61%). Using the general continuous-flow procedure and **7i** (145 mg, 0.50 mmol), the title compound **9i** was obtained as a colorless solid after column chromatography (63 mg, 0.20 mmol, 40%). *R*_*f*_ = 0.40 (9/1 hexanes/ethyl acetate); ^1^H NMR (CDCl_3_, 500 MHz): δ 7.66–7.55 (m, 4H), 7.55–7.46 (m, 2H), 7.22 (d, 2H, *J* = 8.2 Hz), 6.73 (d, 1H, *J* = 3.5 Hz), 6.68 (d, 1H, *J* = 3.5 Hz), 2.38 (s, 3H); ^13^C {^1^H} NMR (CDCl_3_, 126 MHz): δ 151.1, 152.0, 137.6, 131.9, 29.9, 129.5, 127.9, 125.2, 123.8, 120.9, 107.9, 106.7, 21.4; IR (ν_max_, cm^–1^): 2980, 1494, 1474, 1399; HRMS (ESI) *m*/*z*: [M + H]^+^ calcd for C_17_H_13_BrOH, 313.0228; found, 313.0221. Crystals suitable for single-crystal X-ray analysis of **9i** were obtained by the slow evaporation of a CDCl_3_ solution. Data matched with that previously reported, but the crystal structure is new.

#### 2-(4-Bromophenyl)-5-phenylfuran **9j**(24f)

Using the batch general procedure and (*E*,*E*)-1-(4-bromophenyl)-4-phenylbuta-1,3-diene (**7j**) (130 mg, 0.50 mmol), rose Bengal disodium salt (1.5 mg, 0.0025 mmol) in 19:1 CH_2_Cl_2_/MeOH (15 mL) provided the intermediate endoperoxide, 3-phenyl-6-(4-bromophenyl)-3,6-dihydro-1,2-dioxine (**8j**), as a colorless solid after column chromatography (21 mg, 0.07 mmol, 14%). *R*_*f*_ = 0.10 (9/1 hexanes/DCM); ^1^H NMR (CDCl_3_, 500 MHz): δ 7.51 (dt, 2H, *J* = 8.6, 2.2 Hz), 7.42–7.35 (m, 5H), 7.34–7.31 (m, 2H), 6.31 (dddd, 2H, *J* = 21.6, 10.3, 2.5, 1.8 Hz), 5.66 (q, 1H, *J* = 2.1 Hz), 5.56 (q, 1H, *J* = 2.1 Hz); ^13^C {^1^H} NMR (CDCl_3_, 126 MHz): δ 137.2, 137.1, 131.8, 130.1, 129.0, 129.7, 128.5, 128.1, 126.8, 122.9, 80.3, 79.4. Using the general batch procedure and **8j** (21 mg, 0.07 mmol), PPh_3_ (21 mg, 0.08 mmol), and CBr_4_ (27 mg, 0.08 mmol), the title compound **9j** was obtained as a colorless solid after column chromatography (17 mg, 0.06 mmol, 86%). *R*_*f*_ = 0.36 (9/1 hexanes/ethyl acetate); ^1^H NMR (CDCl_3_, 500 MHz): δ 7.75–7.73 (m, 2H), 7.62–7.59 (m, 2H), 7.53 (dt, 2H, *J* = 8.8, 2.0 Hz), 7.41 (t, 2H, *J* = 7.8 Hz), 7.29 (t, 1H, *J* = 7.4 Hz), 6.74 (t, 2H, *J* = 3.9 Hz); ^13^C {^1^H} NMR (CDCl_3_, 126 MHz): δ 153.8, 152.4, 131.9, 130.6, 129.8, 128.8, 127.7, 125.2, 123.9, 121.1, 107.9, 107.4.

#### 3-Hexyl-2,5-diphenylfuran **9k**(24g)

Using the batch general procedure and ((1*E*,3*E*)-2-hexyl-1,4-diphenylbuta-1,3-diene (**7k**) (290 mg, 1.00 mmol), rose Bengal disodium salt (3 mg, 0.005 mmol) in 19:1 CH_2_Cl_2_/MeOH (30 mL) provided the intermediate endoperoxide, 5-hexyl-3,6-diphenyl-3,6-dihydro-1,2-dioxine (**8k**), as a colorless solid after column chromatography (200 mg, 0.62 mmol, 62%). *R*_*f*_ = 0.44 (9/1 hexanes/ethyl acetate); ^1^H NMR (CDCl_3_, 500 MHz): δ 7.51–7.31 (m, 10H), 6.02–6.00 (m, 1H), 5.69 (s, 1H), 5.36 (s, 1H), 2.06–1.88 (m, 2H), 1.51–1.39 (m, 2H), 1.28–1.18 (m, 6H), 0.86 (t, 3H, *J* = 6.5 Hz); ^13^C {^1^H} NMR (CDCl_3_, 126 MHz): δ 138.1, 129.4, 128.8, 128.7, 128.6, 128.5, 128.5, 121.9, 83.2, 80.6, 32.6, 31.7, 29.0, 27.2, 22.6, 14.1. Using the general batch procedure and **8k** (149 mg, 0.46 mmol), PPh_3_ (134 mg, 0.51 mmol), and CBr_4_ (170 mg, 0.51 mmol), the title compound **9k** was obtained as a colorless solid after column chromatography (90 mg, 0.30 mmol, 64%). Using the general continuous-flow procedure and **7k** (145 mg, 0.50 mmol), the title compound **9k** was obtained as a colorless solid after column chromatography (88 mg, 0.19 mmol, 58%). *R*_*f*_ = 0.51 (9/1 hexanes/ethyl acetate); ^1^H NMR (CDCl_3_, 500 MHz): δ 7.89–7.65 (m, 4H), 7.50–7.34 (m, 4H), 7.34–7.13 (m, 3H), 6.67 (s, 1H), 2.70 (t, 2H, *J* = 7.5 Hz), 1.76–1.63 (m, 2H), 1.48–1.38 (m, 2H), 1.38–1.24 (m, 4H), 0.90 (t, 3H, *J* = 7 Hz); ^13^C {^1^H} NMR (CDCl_3_, 126 MHz): δ 152.0, 148.0, 131.9, 130.9, 128.6, 128.6, 127.2, 126.9, 125.6, 124.2, 123.7, 109.2, 31.8, 30.0, 29.3, 26.1, 22.7, 14.2; IR (ν_max_, cm^–1^): 2926, 2855, 1663, 1595, 1492, 1482, 1447; HRMS (ESI) *m*/*z*: [M + H]^+^ calcd for C_22_H_24_OH, 305.1900; found, 305.1899.

#### 1,4-Bis(4-hexyl-5-phenylfuran-2-yl)benzene **9m**

Using the batch general procedure and (1,4-bis((*E*)-3-((*E*)-benzylidene)non-1-en-1-yl)benzene (**7m**) (502 mg, 1.00 mmol), rose Bengal disodium salt (3 mg, 0.005 mmol) in 19:1 CH_2_Cl_2_/MeOH (30 mL) provided the intermediate endoperoxide **8m** as a colorless solid after column chromatography (95 mg, 0.17 mmol, 17%). *R*_*f*_ = 0.15 (19/1 hexanes/ethyl acetate); ^1^H NMR (CDCl_3_, 500 MHz): δ 7.53–7.30 (m, 14H), 6.00 (s, 2H), 5.69 (s, 2H), 5.37 (s, 2H), 2.03–1.86 (m, 4H), 1.51–1.20 (m, 16H), 0.86 (t, 6H, *J* = 7 Hz); ^13^C {^1^H} NMR (CDCl_3_, 126 MHz): δ 138.3, 137.4, 129.4, 128.9, 128.7, 128.6, 128.5, 121.7, 83.3, 80.3, 32.6, 31.7, 29.0, 27.2, 22.6, 14.1. Using the general batch procedure and **8m** (94 mg, 0.17 mmol), PPh_3_ (50 mg, 0.19 mmol), and CBr_4_ (63 mg, 0.19 mmol), the title compound **9m** was obtained as a colorless solid after column chromatography (22 mg, 0.04 mmol, 24%). Using the general continuous-flow procedure and **7m** (251 mg, 0.50 mmol), the title compound **9m** was obtained as a colorless solid after column chromatography (63 mg, 0.20 mmol, 24%). *R*_*f*_ = 0.51 (19/1 hexanes/ethyl acetate); ^1^H NMR (CDCl_3_, 400 MHz): δ 7.60–7.66 (m, 8H), 7.48–7.41 (m, 4H), 7.32–7.26 (m, 2H), 6.69 (s, 2H), 2.71 (t, 4H, *J* = 8 Hz), 1.79–1.65 (m, 4H), 1.49–1.31 (m, 12H), 0.90 (t, 6H, *J* = 7.2 Hz); ^13^C {^1^H} NMR (CDCl_3_, 100 MHz): δ 151.8, 148.1, 131.8, 129.5, 128.6, 126.9, 125.6, 124.4, 124.0, 109.4, 31.8, 30.0, 29.3, 26.2, 22.7, 14.2; HRMS (ESI) *m*/*z*: [M + H]^+^ calcd for C_38_H_42_O_2_, 530.3185; found, 530.3179.

### Continuous-Flow Synthesis General Procedure

A solution of diene in CH_2_Cl_2_ (0.025 M) containing tetraphenylporphyrin (10^–4^ M) was pumped at 0.30 mL/min combining with an air stream at 1.85 mL/min into a “Y” junction under 9.0 bar back pressure. The resulting segmented flow stream was passed through a UV-150 reactor coil equipped with a 450 nm LED light source (10 mL, τ = 30 min) at 35 °C. The resulting output stream was returned to atmospheric pressure and combined with a stream of preformed Appel reagent (PPh_3_/CBr_4_, 0.05 M in CH_2_Cl_2_ stirred for 30 min) pumped at 0.30 mL/min. The reaction stream was split and recombined through two “T” splitters to aid with reagent mixing and pumped through a second reactor coil held at 25 °C (10 mL, τ = 33 min). The resulting output stream was collected, solvents were removed in vacuo, and the residue was purified by column chromatography.

Using these procedures, the following substrates were synthesized.

#### 2,5-Diphenylfuran **2**(11c)

Using the general continuous-flow procedure and **1** (2.06 g, 10.0 mmol), the title compound **2** was obtained as a colorless solid after column chromatography (1.17 g, 5.30 mmol, 53%). The data was in agreement with the batch process given above.

#### 2,5-Bis(4-bromophenyl)furan **9a**(24a)

Using the general continuous-flow procedure and **7a** (91 mg, 0.25 mmol), the title compound **9a** was obtained as a colorless solid after column chromatography (77 mg, 0.20 mmol, 81%). The data was in agreement with the batch process given above.

#### 2,5-Di-*p*-tolylfuran **9b**(24a)

Using the general continuous-flow procedure and **7b** (117 mg, 0.50 mmol), the title compound **9b** was obtained as a colorless solid after column chromatography (71 mg, 0.29 mmol, 57%). The data was in agreement with the batch process given above.

#### 2,5-Bis(4-(trifluoromethyl)phenyl)furan **9c**(24b)

Using the general continuous-flow procedure and **7c** (171 mg, 0.50 mmol), the title compound **9c** was obtained as a colorless solid after column chromatography (107 mg, 0.30 mmol, 60%). The data was in agreement with the batch process above.

#### 2,5-Bis(3-bromophenyl)furan **9e**(24c)

Using the general continuous-flow procedure and **7e** (182 mg, 0.50 mmol), the title compound **9e** was obtained as a colorless solid after column chromatography (123 mg, 0.34 mmol, 65%). The data was in agreement with the batch process above.

#### 2-(4-Bromophenyl)-5-(4-methylphenyl)furan **9i**(24e)

Using the general continuous-flow procedure and **7i** (145 mg, 0.50 mmol), the title compound **9i** was obtained as a colorless solid after column chromatography (63 mg, 0.20 mmol, 40%). The data agreed with the batch process given above.

#### 3-Hexyl-2,5-diphenylfuran **9k**(23)

Using the general continuous-flow procedure and **7k** (145 mg, 0.50 mmol), the title compound **9k** was obtained as a colorless solid after column chromatography (88 mg, 0.19 mmol, 58%). The data was in agreement with the batch process given above.

#### 3-Hexyl-2-(2-naphthyl)-5-phenylfuran **9l**

Using the general continuous-flow procedure and (*E*,*E*)-1-phenyl-2-hexyl-4-(2-naphthyl)buta-1,3-diene (**8l**) (128 mg, 0.38 mmol) and rose Bengal disodium salt (1.5 mg, 0.002 mmol) in 19:1 CH_2_Cl_2_/MeOH (15 mL), the title compound **9l** was obtained as a colorless solid after column chromatography (68.9 mg, 0.19 mmol, 51%). *R*_*f*_ = 0.36 (9/1 hexanes/ethyl acetate); ^1^H NMR (CDCl_3_, 400 MHz): δ 8.19 (s, 1H), 7.90–7.80 (m, 4H), 7.76–7.74 (m, 2H), 7.54–7.38 (m, 4H), 7.31 (t, 1H, *J* = 7.4, 1.4 Hz), 6.79 (s, 1H), 2.74 (t, 2H, *J* = 7.8 Hz), 1.81–1.66 (m, 2H), 1.52–1.29 (m, 6H), 0.91 (t, 3H, *J* = 7.0 Hz); ^13^C {^1^H} NMR (CDCl_3_, 100 MHz): δ 152.1, 148.4, 133.7, 131.8, 128.7, 128.5, 128.4, 128.3, 128.2, 127.8, 127.0, 126.5, 125.9, 125.7, 124.4, 122.4, 121.9, 109.9, 31.8, 30.1, 29.3, 26.2, 22.7, 14.2; HRMS (ESI) *m*/*z*: [M + K]^+^ calcd for C_26_H_26_OK, 393.1615; found, 393.1824.

#### 1,4-Bis(4-hexyl-5-phenylfuran-2-yl)benzene **9m**

Using the general continuous-flow procedure and **7m** (251 mg, 0.50 mmol), the title compound **9m** was obtained as a colorless solid after column chromatography (63 mg, 0.20 mmol, 24%). The data was in agreement with the batch process given above.

## Data Availability

The data underlying this study are available in the published article and its Supporting Information.

## Supporting Information Available

Experimental procedures, compound characterization, crystallographic data (CCDC 2234724–8), computational figures, and copies of NMR spectra (PDF)

## Accession Codes

CCDC 2234724–2234728 contain the supplementary crystallographic data for this paper. These data can be obtained free of charge via www.ccdc.cam.ac.uk/data_request/cif, by emailing data_request@ccdc.cam.ac.uk, or by contacting the Cambridge Crystallographic Data Centre, 12 Union Road, Cambridge CB2 1EZ, UK; fax: +44 1223 336033.
