# Supplementary material for: Transition-Metal-Free Continuous-Flow Synthesis of 2,5-Diaryl Furans: Access to Medicinal Building Blocks and Optoelectronic Materials
Source: J Org Chem. 2023 Dec 25;89(1):484–97. doi: 10.1021/acs.joc.3c02237 (PMC10777415; doi:10.1021/acs.joc.3c02237)

Transition metal free continuous flow synthesis of 2,5-diaryl furans: access to medicinal building blocks and optoelectronic materials.

Helena F. Grantham,[1] Robert J. Lee,[1] Grzegorz M. Wardas,[1] Jai-Ram Mistry,[1] Mark R. J. Elsegood,[1] Iain A. Wright,[2*] Gareth J. Pritchard[1*] and Marc C. Kimber[1*]

[1]*Department of Chemistry, School of Science, Loughborough University, LE11 3TU, UK.*

[2]*The School of Chemistry, University of Edinburgh, Joseph Black Building, Edinburgh, EH9 3FJ, UK.*

M.C.Kimber@lboro.ac.uk

G.J.Pritchard@lboro.ac.uk

Iain.wright@ed.ac.uk

Supporting Information

Table of contents S1

General information S2

Synthetic procedures for preparation of 1,3-dienes **7a-l** S2-S11

Batch photooxidation S12

Flow optimisation of the preparation of **3** and **2** S13

Computational Results S14-S23

X-ray data for **7a**, **7i**, **7m**, **9c,** and **9i** S24-S36

References S37

NMR spectra of prepared compounds - benzylacrylaldehyde S38-S45

NMR spectra of prepared compounds – 1,3-dienes S46-S58

NMR spectra of prepared compounds – endoperoxides S59-S70

NMR spectra of prepared compounds – furans S71-S83

General information

All reactants and reagents were purchased from commercial suppliers and used without further purification unless otherwise stated. Column chromatography was carried out using silica gel 60, 40−60 µm mesh (Apollo Scientific) and Aluminium oxide 90 active neutral 0.063−0.200 mm (70−230 mesh ASTM, Merck). Analytical thin-layer chromatography was performed on precoated aluminum silica gel 60 F254 plates (Merck) and Macherey-Nagel ALUGRAM Alox N/UV_{254} Aluminum Sheets (Fisher), which were approximately 2 × 6 cm in size and visualized using ultraviolet light (254/365 nm).

NMR spectra were recorded on Jeol ECS 400 MHz and Jeol ECZ 500 MHz spectrometers. Chemical shifts are reported in ppm downfield of tetramethylsilane (TMS) using TMS or the residual solvent as an internal reference. NMR spectra were processed using MestReNova. Multiplicities are reported as singlet (s), doublet (d), triplet (t), and multiplet (m). Melting points were determined in open-ended capillaries using a Stuart Scientific SMP10 melting point apparatus at a ramping rate of 1 °C/min. They are recorded to the nearest 1 °C and are uncorrected. IR spectra were collected on a Thermo Scientific Nicolet FTIR spectrometer. Atmospheric solids analysis probe (ASAP) mass spectra were recorded on a Waters LCT Premier XE spectrometer. The sample was introduced as a solid, applied directly to a glass probe tip. Matrix-assisted laser desorption time-of-flight (MALDI–TOF) mass spectra were recorded on a Bruker Daltonik Autoflex II spectrometer running in positive ion reflection mode.

UV−vis absorbance was measured using a UV-1800 UV−vis spectrophotometer (Shimadzu) and UVProbe version 2.33 software. Emission spectra were recorded on a SPEX Fluoromax luminescence spectrometer using dM300 version 3.12 software.

Electrochemistry was performed using Autolab PGSTAT128 potentiostat using NOVA 2.1 software. The working electrode was washed with CH_2Cl_2 and polished with alumina between measurements. Solutions were degassed by sparging with Ar and the experiments run under a blanket of Ar. Potential limits chosen were dictated by the nature of the analyte.

Synthetic procedures for preparation of 1,3-dienes 7a-l

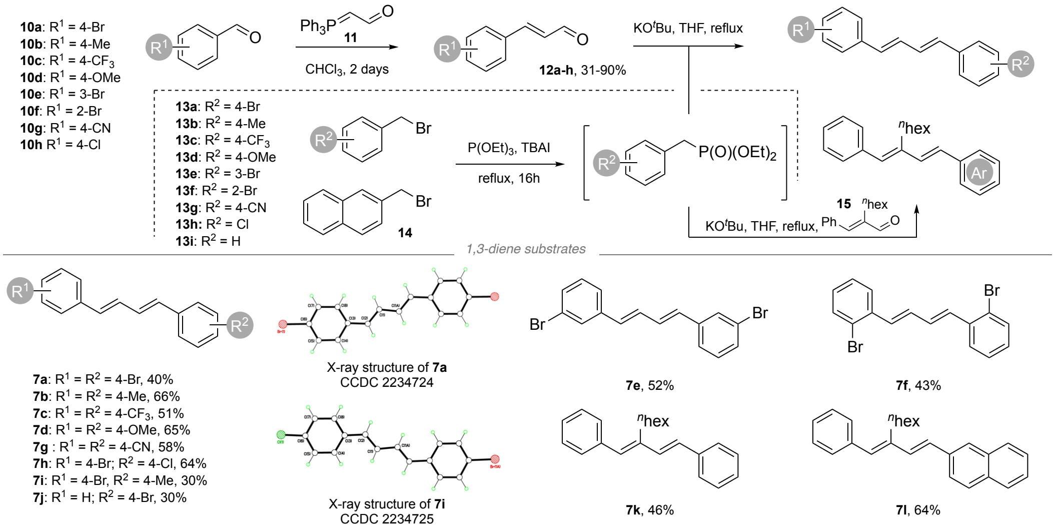

Scheme S1. Preparation of 1,3-dienes **7a-l**

General procedure used for synthesising arylacylaldehydes 12a-h.

To a solution of benzaldehyde (5.00 mmol) in $CHCl_3$ (50 mL) was added (triphenylphosphoranylidene)acetaldehyde **11** (1.70 g, 5.60 mmol), and the mixture heated to reflux for 48 h. After this period the mixture was cooled, and solvents were removed *in vacuo*. The crude material was purified by column chromatography providing the following substrates:

3-(4-Bromophenyl)acrylaldehyde 12a.

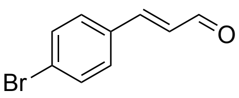

Prepared from 4-bromobenzaldehyde **10a** (930 mg, 5.00 mmol) using the general procedure. The crude material was purified by column chromatography (4:1, hexanes/ethyl acetate, R_f = 0.24) to give the title compound (629 mg, 3.00 mmol, 60 %). ^{1}H NMR (500 MHz, $CDCl_3$) δ 9.71 (d, *J* = 7.6 Hz, 1H), 7.59 – 7.56 (m, 2H), 7.49 – 7.38 (m, 3H), 6.70 (dd, *J* = 6.0 Hz, 16.0 Hz, 1H); ^{13}C{^{1}H} NMR (126 MHz, $CDCl_3$) δ 193.4, 151.2, 133.0, 132.5, 129.9, 129.1, 125.8; IR (ν_{max}, cm^{-1}): 1470, 1623, 1580, 1562, 1486, 1406; HRMS (ESI) m/z: $[M + H]^+$ Calcd for C_9H_7BrOH 210.9753; Found 210.9754. Data consistent with that reported in the literature.[1]

3-(*p*-Tolyl)acrylaldehyde 12b.

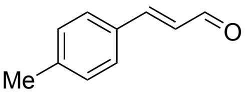

To a solution of *p*-tolualdehyde **10b** (2.40 mL, 20.0 mmol) and sodium hydroxide (80 mg, 2.0 mmol) in ethanol (50 mL) and water (10 mL) cooled to 0 °C was added a solution of acetaldehyde (2.80 mL, 50.0 mmol) in ethanol (20 mL) dropwise over a 2 h period. The resulting mixture was allowed to stir at 0 °C for 16 h, then the solution was acidified to pH 5 with 1M HCl solution. The mixture was concentrated to *ca.* 20 mL in vacuo, then extracted with ethyl acetate (3 x 50 mL), the combined organics washed with saturated $NaHCO_3$ solution (20 mL), brine (20 mL), then dried over $MgSO_4$, filtered, and solvents removed *in vacuo*. The crude material was purified by column chromatography (4:1, hexanes/ethyl acetate, R_f = 0.32), to give the product as a colourless oil (0.89 g, 6.1 mmol, 31%). ^{1}H NMR (500 MHz, $CDCl_3$) δ 9.68 (d, *J* = 7.4 Hz, 1H), 7.51 – 7.42 (m, 3H), 7.24 (d, *J* = 8 Hz, 2H), 6.69 (dd, *J* = 8.0, 16.0 Hz, 1H), 2.40 (s, 3H); ^{13}C{^{1}H} NMR (100 MHz, $CDCl_3$) δ 193.9, 153.0, 142.1, 131.4, 129.9, 128.6, 127.9, 21.7; IR ($ν_{max}$, cm^{-1}): 1670, 1622, 1604, 1568, 1511, 1421; HRMS (ESI) m/z: $[M + H]^+$ Calcd for $C_{10}H_{10}OH$ 147.0805; Found 147.0804. Data consistent with that reported in the literature.[1]

3-(4-(Trifluoromethyl)phenyl)acrylaldehyde 12c.

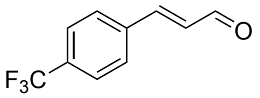

Prepared from 4-(trifluoromethyl)benzaldehyde **10c** (870 mg, 5.00 mmol) using the general procedure. The crude material was purified by column chromatography (4:1, hexanes/ethyl acetate, R_f = 0.26) to give the title compound (812 mg, 3.00 mmol, 81 %). ^{1}H NMR (400 MHz, $CDCl_3$) δ 9.74 (d, *J* = 7.5 Hz, 1H), 7.71 – 7.67 (m, 4H), 7.50 (d, *J* = 16 Hz, 1H), 6.76 (dd, *J* = 7.6 Hz, 16.0 Hz, 1H); ^{13}C{^{1}H} NMR (100 MHz, $CDCl_3$) δ 193.4, 150.5, 137.4, 132.7 (q, J_{CF} = 32.5 Hz), 130.5, 128.7, 126.1 (q, J_{CF} = 3.8 Hz), 123.8 (q, J_{CF} = 271.7 Hz); **IR ($ν_{max}$, cm^{-1}):** 2803, 2730, 1677, 1629, 1577, 1420. Data consistent with that reported in the literature.[1]

3-(4-Methoxyphenyl)acrylaldehyde 12d.

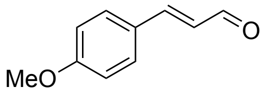

Prepared from 4-methoxybenzaldehyde **10d** (680 mg, 5.00 mmol) using the general procedure. The crude material was purified by column chromatography (4:1, hexanes/ethyl acetate, R_f = 0.18) to give the title compound (700 mg, 4.45 mmol, 89 %, mixture of E/Z isomers). ^{1}H NMR (400 MHz, $CDCl_3$) δ *E*-isomer: 9.65 (d, *J* = 7.8 Hz, 1H), 7.52 (m, 2H), 7.44 (m, 1H), 6.95 (dd, *J* = 11.7, 3.1 Hz, 2H), 6.61 (dd, *J* = 7.6, 16.0 Hz, 1H), 3.86 (s, 3H); *Z*-isomer: 9.59 (d, *J* = 8.2 Hz, 1H), 7.47 – 7.44 (m, 2H), 7.28 – 7.22 (m, 1H), 6.91 (dd, *J* = 6.2, 2.5 Hz, 2H), 6.23 (dd, *J* = 7.6, 16.0 Hz, 1H), 3.84 (s, 3H^{13}C{^{1}H} NMR (100 MHz, $CDCl_3$) δ *E*-isomer: 193.8, 162.3, 152.8, 130.4, 126.9, 126.6, 114.7, 55.5; Z-isomer: 193.8, 161.0, 142.4, 130.7, 129.2, 128.5, 114.5, 55.5; HRMS (ESI) m/z: $[M + Na]^+$ Calcd for $C_{10}H_{10}O_2Na$ 185.0575; Found 185.0574. Data consistent with that reported in the literature.[1]

3-(3-Bromophenyl)acrylaldehyde 12e.

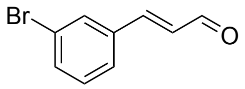

Prepared from 3-bromobenzaldehyde **10e** (930 mg, 5.00 mmol) using the general procedure. The crude material was purified by column chromatography (4:1, hexanes/ethyl acetate, R_f = 0.28) to give the title compound (290 mg, 3.27 mmol, 66 %). ^{1}H NMR (500 MHz, $CDCl_3$) δ 9.71 (d, *J* = 7.6 Hz, 1H), 7.71 (t, *J* = 2.0 Hz, 1H), 7.58 – 7.56 (m, 1H), 7.49 (d, *J* = 7.9 Hz, 1H), 7.40 (d, *J* = 15.8 Hz, 1H), 7.31 (t, *J* = 8 Hz, 1H), 6.70 (dd, *J* = 8 Hz, 16.5 Hz, 1H); ^{13}C{^{1}H} NMR (126 MHz, $CDCl_3$) δ 193.4, 150.8, 136.1, 134.1, 131.1, 130.7, 129.7, 127.0, 123.3; IR ($ν_{max}$, cm^{-1}): 2980, 2602, 1672, 1623, 1564, 1469, 1418; HRMS (ESI) m/z: $[M + H]^+$ Calcd for C_9H_7BrOH 210.9753; Found 210.9753. Data consistent with that reported in the literature.[2]

3-(2-Bromophenyl)acrylaldehyde 12f.

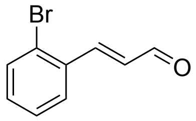

Prepared from 2-bromobenzaldehyde **10f** (930 mg, 5.00 mmol) using the general procedure. The crude material was purified by column chromatography (4:1, hexanes/ethyl acetate, R_f = 0.28) to give the title compound (846 mg, 4.00 mmol, 80 %). ^{1}H NMR (400 MHz, $CDCl_3$) δ 9.76 (d, *J* = 7.8 Hz, 1H), 7.89 (d, *J* = 16.0 Hz, 1H), 7.66 – 7.64 (m, 2H), 7.39 – 7.35 (m, 1H), 7.30 – 7.27 (m, 1H), 6.66 (dd, *J* = 16 Hz, 7.6 Hz, 1H); ^{13}C{^{1}H} NMR (100 MHz, $CDCl_3$) δ 193.6, 150.7, 133.9, 133.7, 132.2, 130.8, 128.1, 128.0, 125.8; IR ($ν_{max}$, cm^{-1}): 2808, 2735, 1672, 1616, 1584, 1559, 1462, 1439, 1426; HRMS (ESI) m/z: $[M + H]^+$ Calcd for C_9H_7BrOH 210.9753; Found 210.9753. Data consistent with that reported in the literature.[3]

4-(3-Oxoprop-1-en-1-yl)benzonitrile 12g.

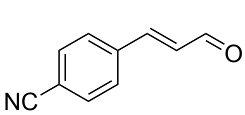

Prepared from 4-formylbenzonitrile **10g** (660 mg, 5.00 mmol) using the general procedure. The crude material was purified by column chromatography (4:1, hexanes/ethyl acetate, R_f = 0.08) to give the title compound (700 mg, 4.45 mmol, 89 %).^{1}H NMR (400 MHz, $CDCl_3$) δ 9.76 (d, *J* = 7.4 Hz, 1H), 7.74 – 7.72 (m, 2H), 7.67 – 7.65 (m, 2H), 7.48 (d, *J* = 16.0 Hz, 1H), 6.77 (dd, *J* = 7.6, 16.4 Hz, 1H); ^{13}C{^{1}H} NMR (100 MHz, $CDCl_3$) δ 192.9, 149.5, 138.2, 132.9, 131.3, 128.8, 118.2, 114.4; IR ($ν_{max}$, cm^{-1}): 2980, 2222, 1675, 1624, 1604, 1559, 1502, 1416, 1392; HRMS (ESI) m/z: $[M + H]^+$ Calcd for $C_{10}H_7NOH$ 158.0600; Found 158.0601. Data consistent with that reported in the literature.[4]

3-(4-Chlorophenyl)acrylaldehyde 12h.

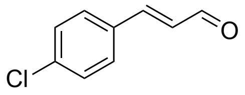

Prepared from 4-chlorobenzaldehyde **10h** (702 mg, 5.00 mmol) using the general procedure. The crude material was purified by column chromatography (9:1, hexanes/ethyl acetate, R_f = 0.16) to give the title compound (502 mg, 4.45 mmol, 60 %). ^{1}H NMR (400 MHz, $CDCl_3$) δ 9.70 (d, *J* = 7.5 Hz, 1H), 7.48 (d, *J* = 8.4 Hz, 2H), 7.43 – 7.38 (m, 3H) 6.68 (dd, *J* = 7.6, 16.0 Hz, 1H); ^{13}C{^{1}H} NMR (100 MHz, $CDCl_3$) δ 193.5, 151.2, 137.4, 132.6, 129.7, 129.5, 129.0; IR (ν_{max}, cm^{-1}): 2980, 2851, 1677, 1625, 1589, 1489, 1411, 1396; HRMS (ESI) m/z: [M + H]$^+$ Calcd for C_9H_7ClOH 167.0258; Found 167.0259. Data consistent with that reported in the literature.[1]

General HWE procedure used for synthesising 1,3-dienes 7a-l.

A mixture of triethyl phosphite (1.30 mL, 7.60 mmol), the benzylbromide **13b** (5.00 mmol), and tetrabutylammonium iodide (160 mg, 0.50 mmol) was heated to reflux for 16 h, after which excess triethyl phosphite was removed *in vacuo*. A portion of the crude benzyl phosphonate (2.50 mmol) was taken and dissolved in anhydrous THF (2.00 mL). The benzylacrylaldehyde (2.50 mmol) was added, followed by KO^{t}Bu (340 mg, 3.00 mmol) in one portion. The resulting mixture was heated to reflux for 1 hour, then the mixture cooled and diluted with water (25 mL) and solids collected by suction filtration. The crude product was then obtained by recrystallisation or column chromatography providing the following substrates.

(*1E,3E*)-1,4-Bis(4-bromophenyl)buta-1,3-diene 7a.

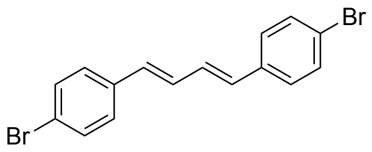

Prepared from 4-bromobenzyl bromide **13a** and 3-(4-bromophenyl)acrylaldehyde **12a** (630 mg, 3.00 mmol) using the general procedure to give the title compound (434 mg, 1.20 mmol, 40 %). ^{1}H NMR (400 MHz, $CDCl_3$) δ 7.49 – 7.42 (m, 4H), 7.35 – 7.28 (m, 4H), 6.97 – 6.85 (m, 2H), 6.67 – 6.56 (m, 2H); ^{13}C{^{1}H} NMR (100 MHz, $CDCl_3$) δ 136.2, 132.2, 131.9, 129.6, 127.9, 121.5; IR (ν_{max}, cm^{-1}): 1579, 1483, 1397, 1071. Data consistent with that reported in the literature.[5] Crystals suitable for single crystal X-ray analysis of **7a** were obtained from d-chloroform.

***(1E,3E)*-1,4-Di-p-tolylbuta-1,3-diene 7b.**

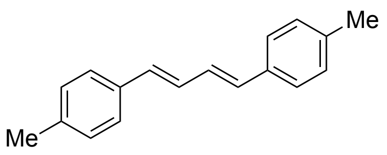

Prepared from 4-methylbenzyl bromide **13b** (0.93 g, 5.00 mmol) and 3-(*p*-tolyl)acrylaldehyde **12b** (290 mg, 2.00 mmol) using the general procedure to give the title compound (308 mg, 1.31 mmol, 66 %). ^{1}H NMR (500 MHz, $CDCl_3$) δ 7.33 (d, *J* = 7.9 Hz, 4H), 7.14 (d, *J* = 8.2 Hz, 4H), 6.95 – 6.83 (m, 2H), 6.67 – 6.57 (m, 2H), 2.35 (s, 6H); ^{13}C{^{1}H} NMR (126 MHz, $CDCl_3$) δ 137.4, 134.8, 132.3, 129.4, 128.6, 126.3, 21.3; IR (ν_{max}, cm^{-1}): 2980, 2911, 1564, 1508, 1444, 129. Data consistent with that reported in the literature.[6]

***(1E,3E)*-1,4-Bis(4-(trifluoromethyl)phenyl)buta-1,3-diene 7c**

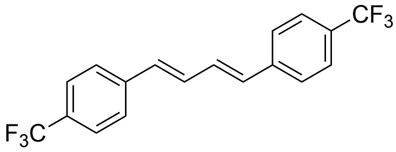

Prepared from 4-(trifluoromethyl)benzyl bromide **13c** (1.20 g, 5.00 mmol) and 4-(trifluoromethyl)benzyl phosphonate **12c** (740 g, 2.50 mmol) using the general procedure to give the title compound (351 mg, 1.03 mmol, 51 %). ^{1}H NMR (400 MHz, $CDCl_3$) δ 7.59 (d, *J* = 8.2 Hz, 4H), 7.54 (d, *J* = 8.2 Hz, 4H), 7.06 – 7.02 (m, 2H), 6.78 – 6.72 (m, 2H); ^{13}C{^{1}H} NMR (100 MHz, $CDCl_3$) δ 140.4, 132.8, 131.0, 129.6 (q, J_{CF} = 32.4 Hz), 126.7, 125.7 (q, J_{CF} = 3.8 Hz), 124.2 (q, J_{CF} = 270.8 Hz); IR (ν_{max}, cm^{-1}): 1611, 1412, 1317. Data consistent with that reported in the literature.[7]

***(1E,3E)*-1,4-Bis(4-(methoxy)phenyl)buta-1,3-diene 7d.**

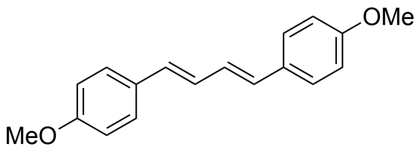

Prepared from 4-methoxybenzyl bromide **13d** (1.00 g, 5.00 mmol) and 3-(4-methoxyphenyl)acrylaldehyde **12d** (324 mg, 2.00 mmol) using the general procedure to give the title compound (345 mg, 1.30 mmol, 65 %). ^{1}H NMR (400 MHz, $CDCl_3$) δ 7.42 – 7.32 (m, 4H), 6.93 – 6.70 (m, 6H), 6.63 – 6.49 (m, 2H), 3.82 (s, 6H); ^{13}C{^{1}H} NMR (100 MHz, $CDCl_3$) δ 159.2, 131.4, 130.5, 127.6, 127.5, 114.2, 55.4; IR (ν_{max}, cm^{-1}): 3012, 2955, 2837, 1597, 1570, 1506, 1464, 1438, 1416, 1301. Data consistent with that reported in the literature.[8]

(*1E,3E*)-1,4-Bis(3-bromophenyl)buta-1,3-diene 7e.

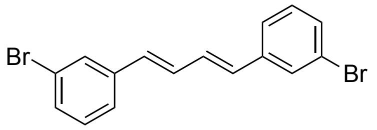

Prepared from 3-bromobenzyl bromide **13e** (1.25 g, 5.00 mmol) and 3-(3-bromophenyl)acrylaldehyde **12e** (420 mg, 2.00 mmol) using the general procedure to give the title compound (378 mg, 1.00 mmol, 52 %). ^{1}H NMR (500 MHz, $CDCl_3$) δ 7.59 (t, *J* = 1.5 Hz, 2H), 7.43 – 7.30 (m, 4H), 7.20 (t, *J* = 7.5 Hz, 2H), 7.00 – 6.84 (m, 2H), 6.69 – 6.53 (m, 2H); ^{13}C{^{1}H} NMR (126 MHz, $CDCl_3$) δ 139.4, 132.2, 130.6, 130.2, 130.2, 129.3, 125.2, 123.0. Data consistent with that reported in the literature.[9]

***(1E,3E)*-1,4-Bis(2-bromophenyl)buta-1,3-diene 7f.**

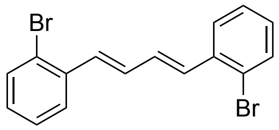

Prepared from 2-bromobenzyl bromide **13f** (1.25 g, 5.00 mmol) and 3-(2-bromophenyl)acrylaldehyde **12f** (423 mg, 2.00 mmol) using the general procedure to give the title compound (312 mg, 0.86 mmol, 43 %). ^{1}H NMR (500 MHz, $CDCl_3$) δ 7.65 (dd, *J* = 7.9, 1.6 Hz, 2H), 7.57 (dd, *J* = 8.1, 1.1 Hz, 2H), 7.30 (t, *J* = 8.0 Hz, 2H), 7.14 – 7.04 (m, 4H), 7.01 – 6.94 (m, 2H); ^{13}C{^{1}H} NMR (126 MHz, $CDCl_3$) δ 136.8, 133.3, 132.3, 131.7, 129.0, 127.6, 126.6, 124.0; IR (ν_{max}, cm^{-1}): 1601, 1581, 1555, 1458, 1432. Data consistent with that reported in the literature.[10]

4,4'-((*1E,3E)*-Buta-1,3-diene-1,4-diyl)dibenzonitrile 7g.

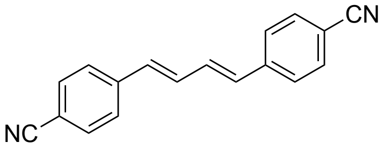

Prepared from 4-cyanobenzyl bromide **13g** (0.98 g, 5.00 mmol) and 4-(3-oxoprop-1-en-1-yl)benzonitrile **12g** (700 mg, 4.50 mmol) using the general procedure to give the title compound (670 mg, 2.60 mmol, 58 %). ^{1}H NMR (500 MHz, $CDCl_3$) δ 7.63 (d, *J* = 8.5 Hz, 4H), 7.52 (d, *J* = 8.5 Hz, 4H), 7.11 – 6.99 (m, 2H), 6.83 – 6.70 (m, 2H); ^{13}C{^{1}H} NMR (126 MHz, $CDCl_3$) δ 141.3, 133.1, 132.6, 131.9, 127.0, 118.9, 111.2; IR (ν_{max}, cm^{-1}): 2218, 1654, 1599, 1498, 1409, 1321. Data consistent with that reported in the literature.[11]

1-(4-Bromophenyl)-4-(4-chlorophenyl)buta-1,3-diene 7h.

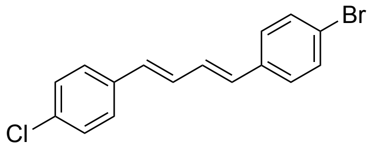

Prepared from 4-chlorobenzyl bromide **13h** (1.03 g, 5.00 mmol) and 3-(4-bromophenyl)acrylaldehyde **12a** (420 mg, 2.00 mmol) using the general procedure to give the title compound (410 mg, 1.30 mmol, 64 %). ^{1}H NMR (400 MHz, $CDCl_3$) δ 7.45 (d, *J* = 8.7 Hz, 2H), 7.36 (d, *J* = 8.7 Hz, 2H), 7.29 (d, *J* = 8.7 Hz, 4H), 6.97 – 6.84 (m, 2H), 6.66 – 6.57 (m, 2H); ^{13}C{^{1}H} NMR (100 MHz, $CDCl_3$) δ 136.2, 135.8, 133.4, 132.1, 132.1, 131.9, 129.7, 129.5, 128.9, 127.9, 127.6, 121.5; IR (ν_{max}, cm^{-1}): 3025, 1585, 1483, 1399; MS (ESI) m/z 318.9 [M + H]$^+$; no molecular ion detected by ESI-HRMS. However, crystals suitable for single crystal X-ray analysis of **7h** were obtained from d-chloroform and confirmed the structure.

1-(4-Bromophenyl)-4-(4-methylphenyl)buta-1,3-diene 7i.

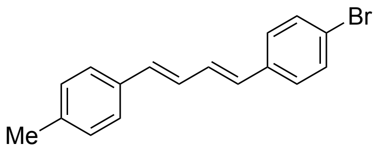

Prepared from 4-methylbenzyl bromide **13b** (930 mg, 5.00 mmol) and 3-(4-bromophenyl)acrylaldehyde **12a** (420 mg, 2.00 mmol) using the general procedure to give the title compound (180 mg, 0.60 mmol, 30 %). ^{1}H NMR (500 MHz, $CDCl_3$) δ 7.45 – 7.43 (m, 2H), 7.34 (d, *J* = 7.9 Hz, 2H), 7.29 (d, *J* = 8.5 Hz, 2H), 7.14 (d, *J* = 7.9 Hz, 2H), 6.97 – 6.81 (m, 2H), 6.66 (d, *J* = 14.8 Hz, 1H), 6.57 (d, *J* = 14.8 Hz, 1H), 2.35 (s, 3H); ^{13}C{^{1}H} NMR (126 MHz, $CDCl_3$) δ 137.8, 136.5, 134.5, 133.6, 131.8, 130.9, 130.2 (C^9), 129.5, 128.0, 127.8, 126.5, 121.1, 21.3; IR (ν_{max}, cm^{-1}): 1509, 1484, 1398; HRMS (ESI) m/z: [M + H]$^+$ Calcd for $C_{17}H_{16}Br$ 299.0435; Found 299.0431. Crystals suitable for single crystal X-ray analysis of **7i** were obtained from d-chloroform. Previously, this compound has been isolated as a mixture.[12]

1-(4-Bromophenyl)-4-phenylbuta-1,3-diene 7j.

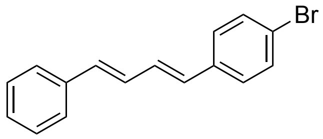

Prepared from bromobenzyl bromide **13i** (860 mg, 5.00 mmol) and 3-(4-bromophenyl)acrylaldehyde **12a** (420 mg, 2.00 mmol) using the general procedure to give the title compound (180 mg, 0.60 mmol, 30 %). ^{1}H NMR (400 MHz, $CDCl_3$) δ 7.47 – 7.43 (m, 4H), 7.37 – 7.23 (m, 5H), 6.98 – 6.90 (m, 2H), 6.71 – 6.58 (m, 2H); ^{13}C{^{1}H} NMR (100 MHz, $CDCl_3$) δ 137.3, 136.4, 133.6, 131.8, 131.5, 130.0, 129.0, 128.8, 127.9, 127.8, 126.5, 121.3. Data consistent with that reported in the literature.[13]

***(1E,3E)*-2-Hexyl-1,4-diphenylbuta-1,3-diene 7k.**

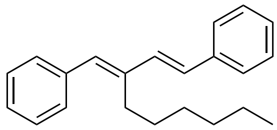

Prepared from benzyl bromide **13i** (855 mg, 5.00 mmol) and hexylcinnamaldehyde **15** (430 mg, 2.00 mmol) using the general procedure to give the title compound as a colourless oil (268 mg, 0.92 mmol, 46 %). ^{1}H NMR (400 MHz, $CDCl_3$) δ 7.48 (d, *J* = 7.4 Hz, 2H), 7.42 – 7.29 (m, 6H), 7.29 – 7.14 (m, 2H), 6.88 (d, *J* = 15.7 Hz, 1H), 6.71 – 6.59 (m, 2H), 2.63 – 2.53 (m, 2H), 1.74 – 1.57 (m, 2H), 1.50 – 1.22 (m, 6H), 0.90 (t, *J* = 6.8 Hz, 3H); ^{13}C{^{1}H} NMR (100 MHz, $CDCl_3$) δ 141.0, 137.9, 137.8, 133.4, 131.9, 128.9, 128.7, 128.4, 127.7, 127.3, 126.7, 126.5, 31.7, 29.7, 29.3, 27.6, 22.7, 14.2; **IR (ν_{max}, cm^{-1}):** 3023, 2953, 2924, 2855, 1593, 1491, 1465, 1444, 1377; HRMS (ESI) m/z: [M + H]$^+$ Calcd for $C_{22}H_{27}$ 291.2113; Found 291.2107.

(*E*,*E*)-1-Phenyl-2-hexyl-4-(2-napthyl)buta-1,3-diene 7l.

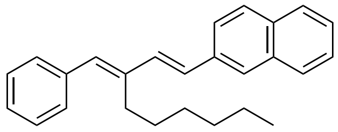

Prepared from 2-(bromomethyl)naphthalene **14** (1.03 g, 5.00 mmol) and hexylcinammaldehyde **15** (420 mg, 2.00 mmol) using the general procedure to give the title compound (210 mg, 0.60 mmol, 64 %). R_f= 0.99, 100% DCM. ^{1}H NMR (400 MHz, $CDCl_3$) δ 7.84 – 7.79 (m, 4H), 7.72 (dd, *J* = 8.9, 1.4 Hz, 1H), 7.50 – 7.36 (m, 6H), 7.29 – 7.25 (m, 1H), 7.03 (d, *J* = 16.1 Hz, 1H), 6.85 (d, *J* = 16.5 Hz, 1H), 6.69 (s, 1H), 2.65 – 2.61 (m, 2H), 1.74 – 1.66 (m, 2H), 1.52 – 1.33 (m, 6H), 0.94 (t, *J* = 7.0 Hz, 3H); ^{13}C{^{1}H} NMR (100 MHz, $CDCl_3$) δ 141.1, 137.9, 135.3, 133.9, 133.8, 133.0, 132.1, 128.9, 128.4, 128.3, 128.0, 127.8, 126.8, 126.4, 125.8, 123.7, 31.7, 29.8, 29.4, 27.6, 22.8, 14.2.

Preparation of 1,4-Bis((*E*)-3-((*E*)-benzylidene)non-1-en-1-yl)benzene 7m.

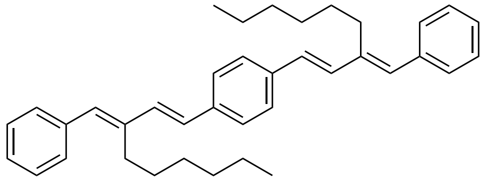

A mixture of triethyl phosphite (2.60 mL, 15.20 mmol), *p*-xylylene dibromide **16** (1.32 g, 5.00 mmol), and tetrabutylammonium iodide (160 mg, 0.50 mmol) was heated to reflux for 16 h, after which excess triethyl phosphite was removed *in vacuo*. The crude bis-phosphonate (1.96 g, 5.00 mmol) was taken, and dissolved in anhydrous THF (2.00 mL). Hexyl cinnamaldehyde **15** (3.40 mL, 14.00 mmol) was added, followed by KO^{t}Bu (680 mg, 6.00 mmol) in one portion. The resulting mixture was heated to reflux for 1 hour, then the mixture cooled and diluted with water (50 mL) and solids collected by suction filtration. The crude product was purified by column chromatography (100 % hexanes, R_f = 0.50) to give

the product as a yellow solid (2.20 g, 4.38 mmol, 88%). ^{1}H NMR (400 MHz, $CDCl_3$) δ 7.44 (s, 4H), 7.41 – 7.29 (m, 8H), 7.29 – 7.21 (m, 2H), 6.90 (d, *J* = 15.7 Hz, 2H), 6.65 (d, *J* = 17.7 Hz, 4H), 2.65 – 2.45 (m, 4H), 1.76 – 1.56 (m, 4H), 1.50 – 1.39 (m, 4H), 1.39 – 1.19 (m, 8H), 0.91 (t, *J* = 5.6 Hz, 6H); ^{13}C{^{1}H} NMR (100 MHz, $CDCl_3$) δ 141.1, 137.9, 136.8, 133.2, 131.9, 128.9, 128.4, 127.4, 126.7, 31.7, 29.8, 29.3, 27.6, 22.8, 14.2; IR (ν_{max}, cm^{-1}): 2980, 2928, 2865, 1679, 1592, 1452, 1441, 1413, 1380; HRMS (ESI) m/z: $[M + H]^+$ Calcd for $C_{38}H_{47}$ 503.3674; Found 503.3678. Crystals suitable for single crystal X-ray analysis of **7m** were obtained from dichloromethane.

Batch photooxidation apparatus

In this work, endoperoxides were prepared by the treatment of analogous 1,3-dienes with singlet oxygen; a process carried out by simultaneously irradiating a solution of the diene either in a dichloromethane/methanol (95:5) mixture containing 10-4 M disodium rose Bengal in, or in dichloromethane containing 10-4 M methylene blue in, with a 400W halogen lamp. Reactions were conducted in a bespoke reaction vessel (Figure SX) which allowed for a constant stream of oxygen to pass through the solution in the form of small bubbles. A constant flow of water through the exterior jacket of the reactor maintained the reaction at a constant temperature, as heat generated from the light source was not insignificant. The apparatus can be seen in Figure SX.

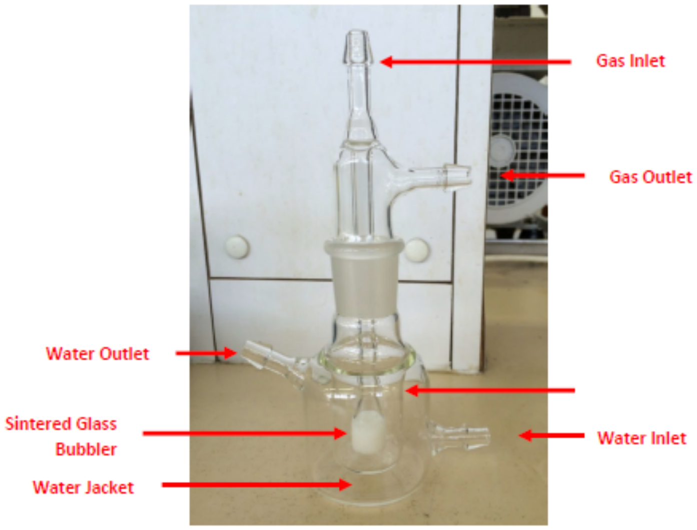

Figure S1: Custom made small scale photoreactor

Flow optimisation for the preparation of 3 & 2

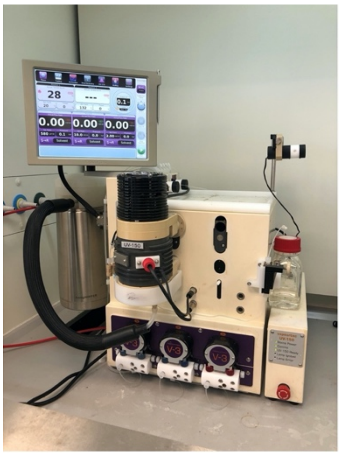

Figure S2a: Vapourtec E-Series (easy-Photochem)

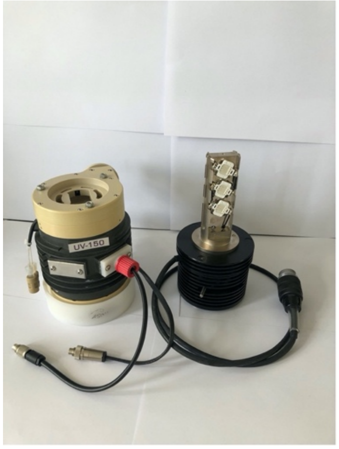

Figure S2b: UV-150 photochemical reactor with interchangeable LEDs

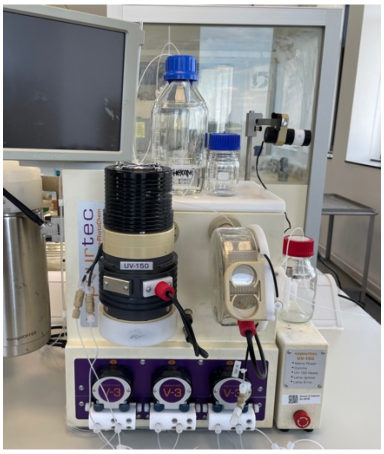

Figure S2c: UV-150 and second reactor

Table S1. Flow optimisation for **3**.[a]

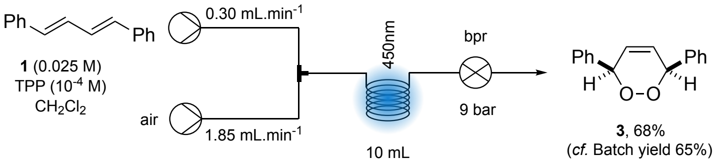

Entry	Diene Flow Rate (mL.min^{-1})	Air Flow Rate (mL.min^{-1})	Back Pressure (bar)	Yield (%)[b]
1	0.30	1.10	0	25
2	0.30	1.10	5	54
3	0.30	1.10	9	60
4	0.30	1.50	9	66
5	0.30	1.85	9	68
6[c]	0.30	1.50	9	-
7[d]	0.30	1.50	9	-

[a] Performed on 0.25 mmol of **1**; [b] Isolated yields; [c] No TPP; [d] No irradiation.

Table S2. Flow optimisation for **2**.[a]

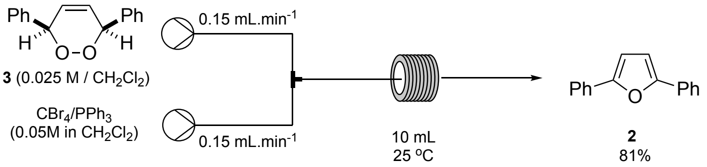

Entry	Endoperoxide Concentration (M)	CBr_4/PPh_3 Concentration (M)	Flow Rate (mL.min^{-1})	Yield (%)[b]
1	0.025	0.03	0.30	76
2	0.025	0.03	0.15	79
3	0.025	0.05	0.15	81

[a] Performed on 0.25 mmol of **1**; [b] Isolated yields.

Computational Results

LUMO

HOMO

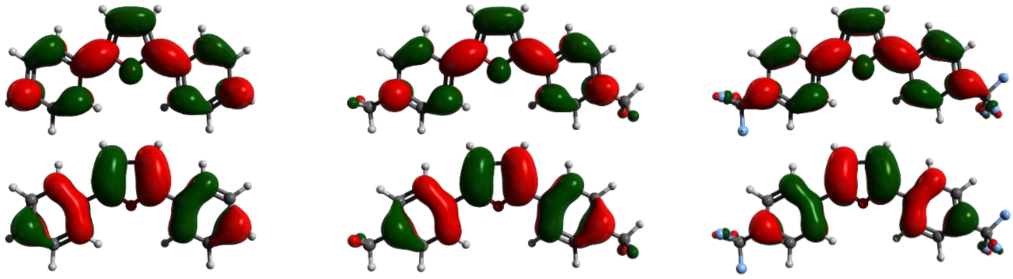

2 9b 9c

LUMO

HOMO

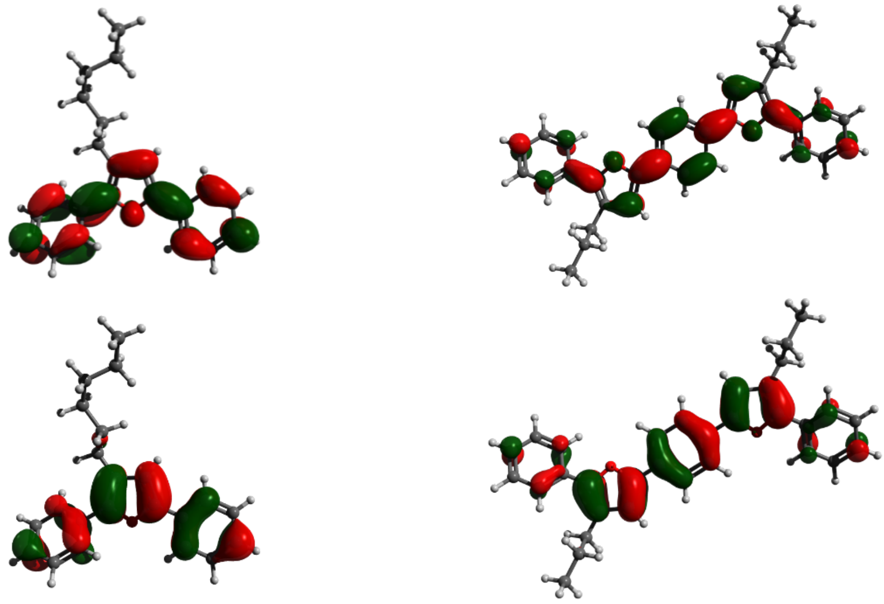

9k 9m

Figure S3. HOMO (lower) and LUMO (upper) distributions for compounds **2, 9b,c,k,** and **m**

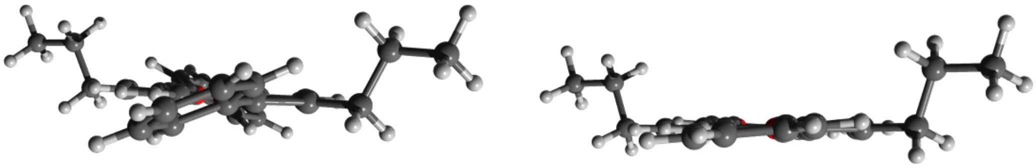

Ground state First excited state

Figure S4. Optimised geometries for the ground state and first excited singlet state of **9m**. The end-on view highlights the greater coplanarity along the conjugated backbone upon excitation.

Compound 2

Cartesian Coordinates (Å)

O	1.052710	0.019299	0.291123
C	0.607423	-0.080046	-0.997301
C	-0.697496	-0.492655	-0.982996
H	-1.323076	-0.663253	-1.842713
C	-1.066115	-0.652965	0.377132
H	-2.025950	-0.968473	0.749627
C	0.030737	-0.329875	1.129207
C	0.288285	-0.294271	2.558846
C	-0.719706	-0.640931	3.470890
H	-1.695756	-0.936434	3.107763
C	-0.481810	-0.609806	4.835869
H	-1.274125	-0.881378	5.522633
C	0.766174	-0.232546	5.325087
H	0.950178	-0.208897	6.391776
C	1.772994	0.113287	4.430579
H	2.747728	0.408500	4.799409
C	1.540592	0.084020	3.062286
H	2.330067	0.354763	2.374915
C	1.540975	0.248415	-2.061390
C	2.854012	0.648790	-1.777751
H	3.178994	0.715005	-0.748666
C	3.735736	0.959188	-2.804104
H	4.746271	1.266490	-2.563959
C	3.330223	0.877866	-4.131631
H	4.020023	1.120475	-4.930113
C	2.027948	0.481381	-4.424808
H	1.700272	0.414447	-5.455004
C	1.143204	0.170349	-3.404432
H	0.134279	-0.135752	-3.649844

Number of Imaginary Frequencies: 0

Final Single Point Energy: −691.976760428160

Compound 9b

Cartesian Coordinates (Å)

O	1.001476	0.013956	0.274501
C	0.554865	-0.088406	-1.014039
C	-0.748915	-0.503541	-0.996967
H	-1.375322	-0.677259	-1.855482
C	-1.115481	-0.663122	0.364388
H	-2.074187	-0.980200	0.738426
C	-0.018228	-0.338310	1.114896
C	0.243295	-0.303728	2.543270
C	-0.756156	-0.657673	3.462344
H	-1.732860	-0.959291	3.105731
C	-0.512691	-0.627587	4.824019
H	-1.305797	-0.906869	5.508931
C	0.732886	-0.245555	5.333500
C	0.984854	-0.217171	6.817722
C	1.723709	0.105149	4.418228
H	2.700153	0.405511	4.781126
C	1.490535	0.079116	3.049450
H	2.280007	0.357457	2.365013
C	1.488370	0.238035	-2.077960
C	2.802992	0.638564	-1.799427
H	3.130546	0.707673	-0.771263
C	3.683408	0.944255	-2.824810
H	4.694269	1.250304	-2.578207
C	3.298373	0.865914	-4.165318
C	4.265764	1.200287	-5.269598
C	1.989013	0.467826	-4.438784
H	1.658494	0.397533	-5.468846
C	1.099404	0.159548	-3.420885
H	0.091717	-0.145108	-3.673422
H	2.003254	0.102220	7.040925
H	0.838306	-1.204171	7.264772
H	0.300645	0.469051	7.324065
H	3.803650	1.086151	-6.250650
H	5.145593	0.552044	-5.236887
H	4.623028	2.230225	-5.186261

Number of Imaginary Frequencies: 0

Final Single Point Energy: −770.582100749957

Compound 9c

Cartesian Coordinates (Å)

O	1.007630	0.008532	0.273378
C	0.556185	-0.091601	-1.012094
C	-0.750163	-0.501858	-0.993152
H	-1.380490	-0.671582	-1.849503
C	-1.113301	-0.660177	0.366678
H	-2.072657	-0.973498	0.742056
C	-0.011640	-0.338621	1.113904
C	0.249873	-0.303755	2.541005
C	-0.756696	-0.651894	3.456155
H	-1.733886	-0.948037	3.098489
C	-0.517364	-0.622480	4.816410
H	-1.305101	-0.894270	5.507343
C	0.735366	-0.244156	5.301812
C	0.964834	-0.222638	6.787432
C	1.741115	0.103237	4.407862
H	2.713941	0.397417	4.776545
C	1.501745	0.074009	3.041577
H	2.291518	0.346165	2.355867
C	1.484360	0.233497	-2.079512
C	2.797673	0.641322	-1.800602
H	3.127111	0.716246	-0.774052
C	3.674503	0.948254	-2.826018
H	4.684361	1.261211	-2.593156
C	3.263963	0.856271	-4.154780
C	4.240691	1.196152	-5.246175
C	1.964079	0.453313	-4.445697
H	1.638186	0.379446	-5.473814
C	1.085506	0.145875	-3.419906
H	0.079044	-0.166053	-3.665542
F	2.213993	0.159882	7.113389
F	0.765885	-1.440258	7.343800
F	0.115289	0.623909	7.414545
F	3.709318	1.062613	-6.476546
F	5.338554	0.406356	-5.201648
F	4.685487	2.470024	-5.143551

Number of Imaginary Frequencies: 0

Final Single Point Energy: −1366.121165528386

Compound 9k

Cartesian Coordinates (Å)

O	1.421696	0.400841	0.477543
C	0.954955	0.314448	-0.810750
C	-0.360602	-0.077910	-0.794482
C	-0.705094	-0.222624	0.583924
C	0.402589	0.077931	1.323314
C	0.672364	0.123966	2.751620
C	-0.316905	-0.243356	3.675497
C	-0.065820	-0.199452	5.037956
C	1.177537	0.210710	5.512115
C	2.166192	0.577002	4.605431
C	1.920049	0.535982	3.239735
C	1.926799	0.617611	-1.856891
C	3.282338	0.308451	-1.668687
C	4.218001	0.589651	-2.653581
C	3.824918	1.182869	-3.849449
C	2.486275	1.505229	-4.043904
C	1.547468	1.233754	-3.057306
C	-1.266164	-0.394731	-1.949130
H	-1.666208	-0.518048	0.971371
H	-1.287662	-0.568141	3.323156
H	-0.843624	-0.487755	5.734510
H	1.371950	0.244152	6.576750
H	3.136696	0.899046	4.962929
H	2.692741	0.827688	2.541756
H	3.593463	-0.161740	-0.745347
H	5.259008	0.339369	-2.489076
H	4.556158	1.399465	-4.618153
H	2.171758	1.985828	-4.962306
H	0.518647	1.532168	-3.205344
H	-0.744436	-0.211481	-2.889092
H	-2.127276	0.283786	-1.935113
C	-1.780437	-1.843704	-1.943462
H	-0.924994	-2.527396	-1.960718
C	-2.702140	-2.140225	-3.127967
H	-2.305044	-2.033555	-1.003798
H	-2.160219	-1.929156	-4.056400
H	-3.547321	-1.441129	-3.108143
C	-3.235297	-3.575524	-3.193395
H	-2.391256	-4.270666	-3.279915
H	-3.811740	-3.687961	-4.118421
C	-4.113270	-4.002447	-2.014899
H	-3.549608	-3.915144	-1.081703

C	-4.634597	-5.432953	-2.149623
H	-4.959721	-3.311536	-1.927122
H	-3.808537	-6.146400	-2.210495
H	-5.239878	-5.548499	-3.052688
H	-5.254331	-5.715662	-1.295988

Number of Imaginary Frequencies: 0

Final Single Point Energy: −927.768595720266

Compound 9l

Cartesian Coordinates (Å)

O	1.348824	0.134826	0.529947
C	0.805514	0.104801	-0.729642
C	-0.541622	-0.158026	-0.635368
C	-0.814209	-0.299717	0.756269
C	0.358590	-0.115700	1.432750
C	0.722886	-0.141064	2.839304
C	-0.233896	-0.327025	3.819503
C	0.103656	-0.357493	5.189096
C	1.469378	-0.191088	5.573141
C	2.434954	-0.001053	4.554279
C	2.080241	0.023874	3.233946
C	1.723346	0.394862	-1.823346
C	2.891901	1.136314	-1.585286
C	3.771939	1.427702	-2.616709
C	3.510145	0.991806	-3.912099
C	2.360507	0.250330	-4.160955
C	1.479993	-0.051108	-3.130770
C	-1.570120	-0.254337	-1.722509
H	-1.778088	-0.505541	1.192929
H	-1.274871	-0.450342	3.545204
H	3.473943	0.124796	4.836901
H	2.833968	0.164992	2.471975
H	3.098877	1.489579	-0.584412
H	4.665486	2.003722	-2.408809
H	4.197395	1.221934	-4.716533
H	2.152654	-0.108583	-5.161646
H	0.613425	-0.661637	-3.339738
H	-1.249631	0.321990	-2.593537
H	-2.491908	0.219309	-1.368989
C	-1.896111	-1.695698	-2.150621
H	-0.982220	-2.185414	-2.504602
C	-2.966654	-1.760251	-3.242177
H	-2.216179	-2.259237	-1.270002
H	-2.642153	-1.144241	-4.088227
H	-3.889744	-1.298668	-2.871238
C	-3.278985	-3.171377	-3.753836
H	-2.353839	-3.639344	-4.111542
H	-3.932020	-3.088622	-4.629679
C	-3.948295	-4.095772	-2.733548
H	-3.293520	-4.232477	-1.867956
C	-4.303596	-5.464456	-3.313090
H	-4.856775	-3.612789	-2.355855

H	-3.411186	-5.985473	-3.669999
H	-4.989721	-5.369572	-4.158990
H	-4.782763	-6.101867	-2.566871
C	-0.870197	-0.547479	6.203058
C	1.805131	-0.222290	6.947177
C	-0.512015	-0.572512	7.525827
C	0.839153	-0.408562	7.903264
H	2.842924	-0.096000	7.234065
H	1.108712	-0.430489	8.951919
H	-1.907411	-0.673378	5.914049
H	-1.266316	-0.718657	8.289248

Number of Imaginary Frequencies: 0

Final Single Point Energy: −1081.369374047393

9m (NB. Ethyl rather than n-hexyl chains were used in the calculations).

Cartesian Coordinates (Å)

O	1.211944	-0.027752	-0.179469
C	0.647998	-0.322871	-1.395539
C	-0.281729	-1.323391	-1.232916
C	-1.127394	-2.011193	-2.262522
C	-0.279522	-1.635234	0.157889
H	-0.887622	-2.384451	0.638354
C	0.641247	-0.827686	0.765484
C	1.086272	-0.672457	2.136779
C	0.571411	-1.485572	3.157150
H	-0.166725	-2.241289	2.920792
C	0.989560	-1.343626	4.466817
H	0.573061	-1.982506	5.233229
C	1.946858	-0.379473	4.819187
C	2.395470	-0.225463	6.189377
C	2.460156	0.434931	3.799113
H	3.199116	1.189940	4.035190
C	2.041427	0.293555	2.489690
H	2.451822	0.938048	1.724682
C	1.171359	0.419158	-2.534833
C	2.462256	0.969554	-2.485170
H	3.060971	0.832447	-1.595227
C	2.977207	1.673213	-3.563541
H	3.976650	2.086310	-3.502829
C	2.221855	1.845160	-4.719573
H	2.626056	2.393815	-5.560893
C	0.939085	1.311982	-4.779867
H	0.334568	1.452157	-5.667624
C	0.415638	0.613056	-3.700566
H	-0.597766	0.242080	-3.753952
C	3.277573	0.619653	6.803058
C	3.302229	0.291701	8.189737
C	2.416351	-0.748939	8.345903
O	1.870409	-1.064923	7.126701
H	3.863160	1.388367	6.325478
C	1.996807	-1.581398	9.465360
C	2.055425	-1.122470	10.789613
C	1.515753	-2.880925	9.237530
C	1.123411	-3.689499	10.293665
H	2.377536	-0.111994	10.996149
C	1.668338	-1.939029	11.843563
C	1.201945	-3.226810	11.603738
H	1.719458	-1.560719	12.857185

H	0.896992	-3.860265	12.427102
H	1.461355	-3.253911	8.224029
H	0.759261	-4.689718	10.093145
C	4.173567	0.959975	9.210985
H	5.153645	1.148145	8.760066
H	4.352349	0.284799	10.051152
C	3.616029	2.294168	9.735361
H	-0.651865	-1.944157	-3.243973
C	-2.565932	-1.474227	-2.353561
H	-1.169948	-3.078191	-2.019614
H	-2.539345	-0.405671	-2.587839
H	-3.039006	-1.551816	-1.370042
C	-3.406555	-2.213331	-3.392457
H	3.455778	2.970054	8.890103
C	4.534910	2.956128	10.759865
H	2.627923	2.126515	10.174739
H	-2.972342	-2.122250	-4.391672
H	-4.423034	-1.817219	-3.435406
H	-3.475069	-3.279376	-3.160212
H	5.518226	3.167046	10.331284
H	4.686941	2.312911	11.630689
H	4.118105	3.901003	11.114274

Number of Imaginary Frequencies: 0

Final Single Point Energy: −1387.568273444852

X-ray data for 7a, 7i, 7m, 9c, and 9i.

Diffraction data were collected on a Rigaku diffractometer[13] equipped with Varimax confocal mirrors and a HyPix 600HE detector at 100 K. The data were corrected for absorption and Lp effects via a multi-scan method based on repeated data.[13] The structures were solved by a dual space charge flipping algorithm[14] and refined by full matrix-least-squares procedures.[15] Full data collection and crystal data details are presented in Tables S3–S7 below and the following paragraphs for the individual structures. Additional views of the molecule and the packing are provided in the figures below. CCDC 2234724-8 contain the supplementary crystallographic data for this paper. These data can be obtained free of charge from The Cambridge Crystallographic Data Centre via www.ccdc.cam.ac.uk/structures.

For **7a**: Half the molecule comprises the asymmetric unit and it lies on an inversion centre. The molecule is essentially co-planar and has *trans trans* geometry at the double bonds.

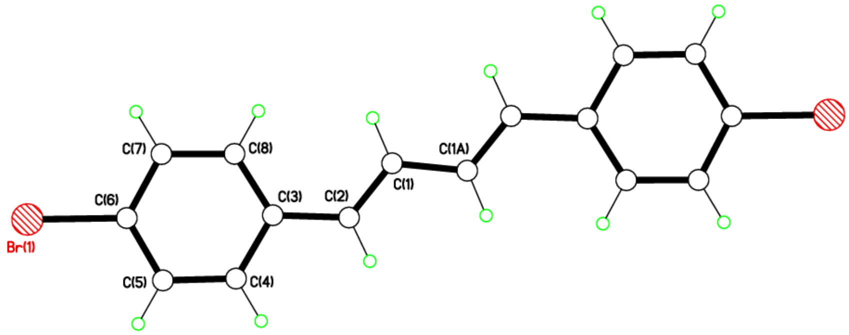

Figure S5. Single molecule of **7a.**

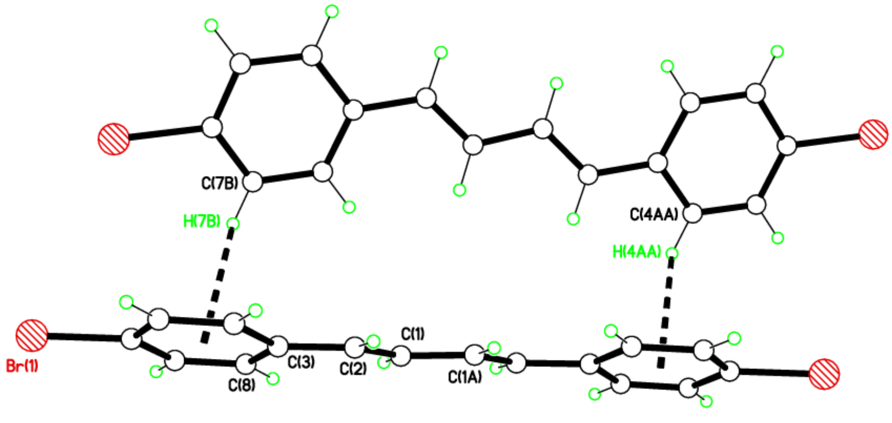

Figure S6. C–H···π interactions between pairs of molecules in **7a**. H to ring centroid distances are 2.75 and 2.76 Å.

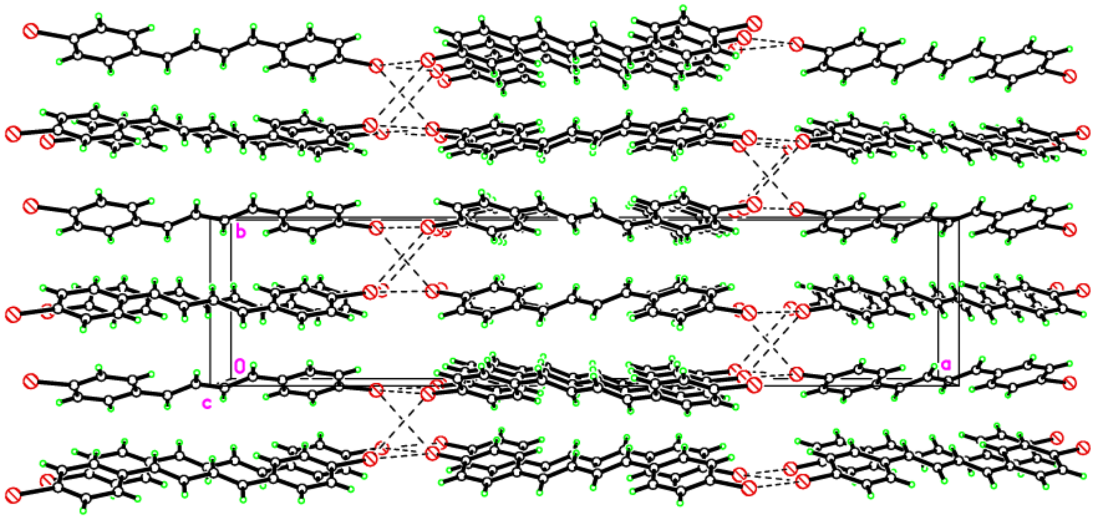

Figure S7. Packing plot of **7a** viewed parallel to *c* showing Br···Br halogen bond interactions at distances 3.762-3.863 Å.

For **7i**: Half of the molecule is the asymmetric unit. The molecule lies on an inversion centre so the Cl/Br are exactly 50/50 disordered. The two different C–X bond lengths were discernible. Restraints were required for the halogen anisotropic displacement parameters due to their close proximity. The twist angle between atoms C(1)/C(2)/C(3) and the aromatic ring C(3) > C(8) is 14.6(3)°. Thus, the molecule is not co-planar. While this is chemically very similar to the Br/Br analogue, **7a**, the unit cells and space group are very different, as is the packing of the molecules.

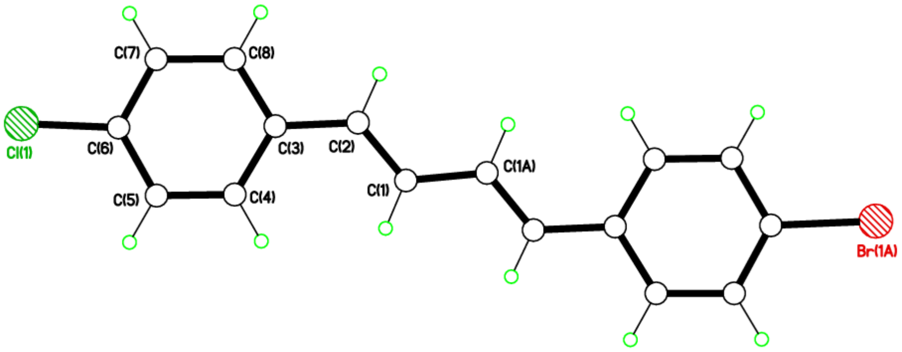

Figure S8. Single molecule of **7i**.

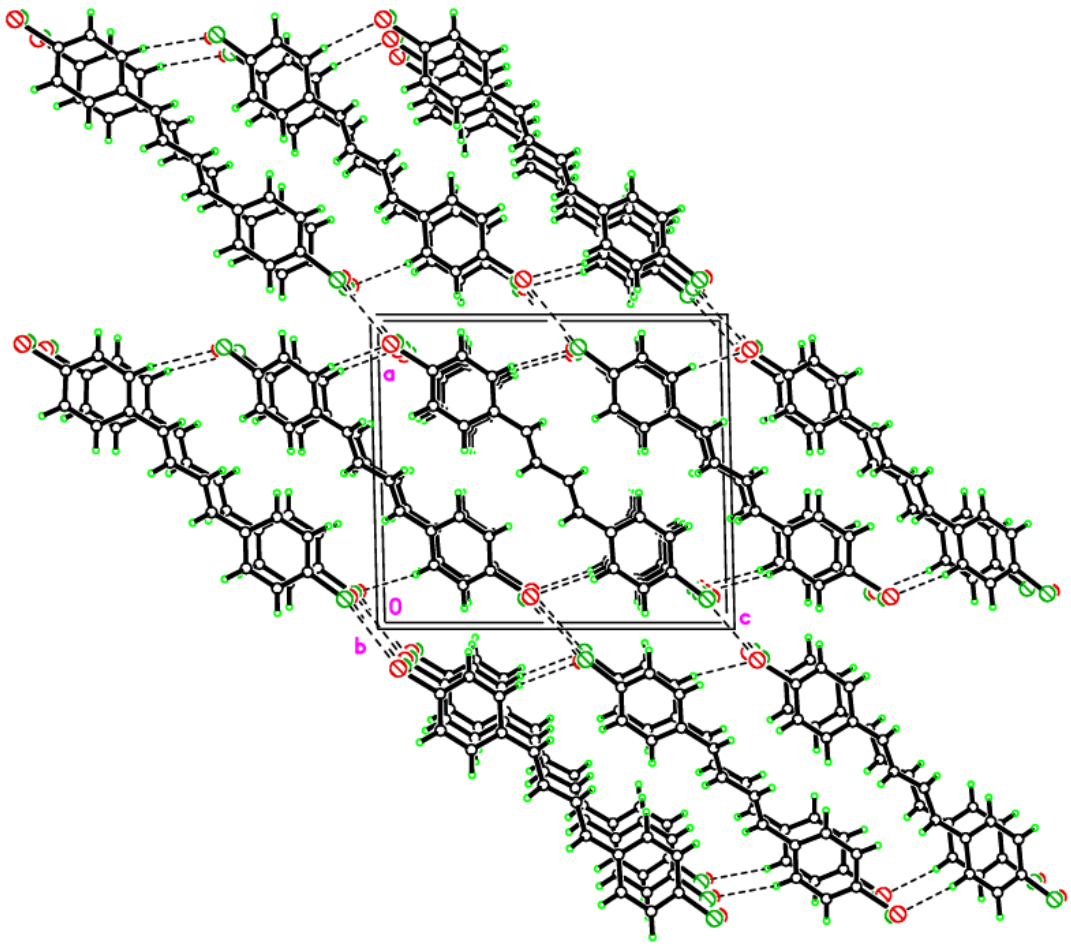

Figure S9. Packing plot of **7i** viewed parallel to *b*. There are some weak Ar(C)-H(8)···X (at 3.03 Å for X = Br) and X···X interactions at 3.557 and 3.585 Å for Br···Br and Br···Cl, respectively, linking molecules within and between layers, respectively.

For **7m**: The molecule lies on an inversion centre so half is unique. Twist angle between the central and the terminal aromatic rings = 64.81(4)□.

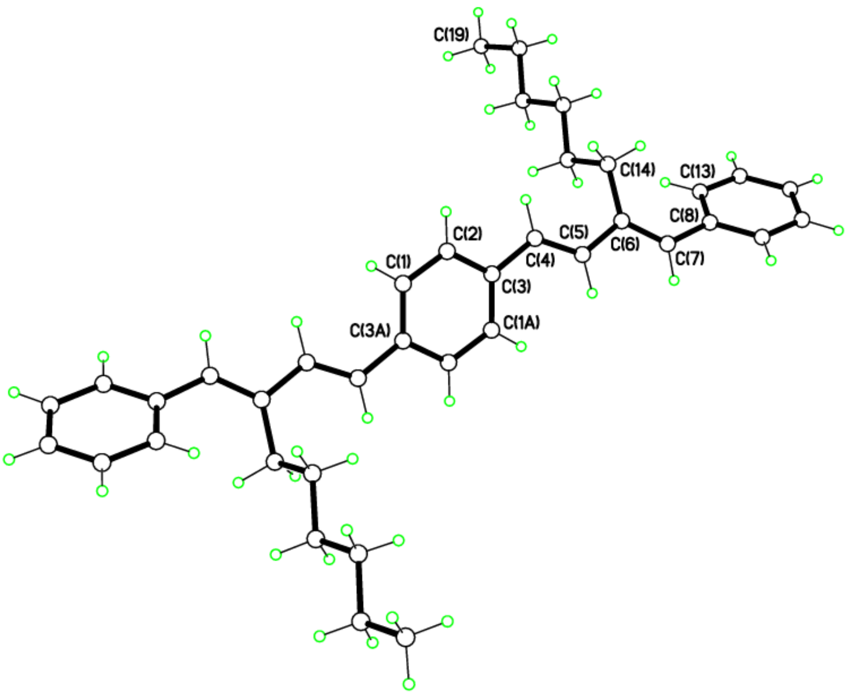

Figure S10. Single molecule of **7m**.

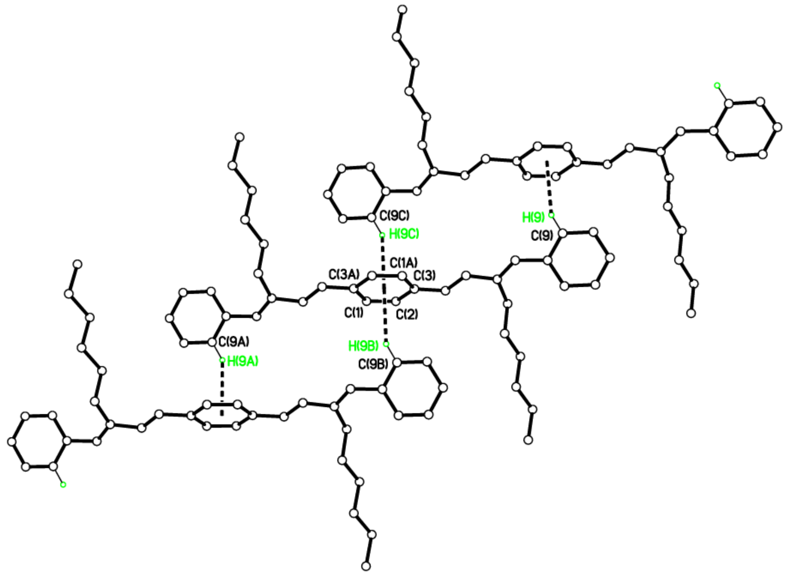

Figure S11. Molecules of **7m** form a stepped ladder motif in the *c* direction, built up from C–H···π interactions with H(9)···ring centroid distances of 2.71 Å. Each molecule has two donors, and the central ring accepts from both faces equally due to the inversion symmetry.

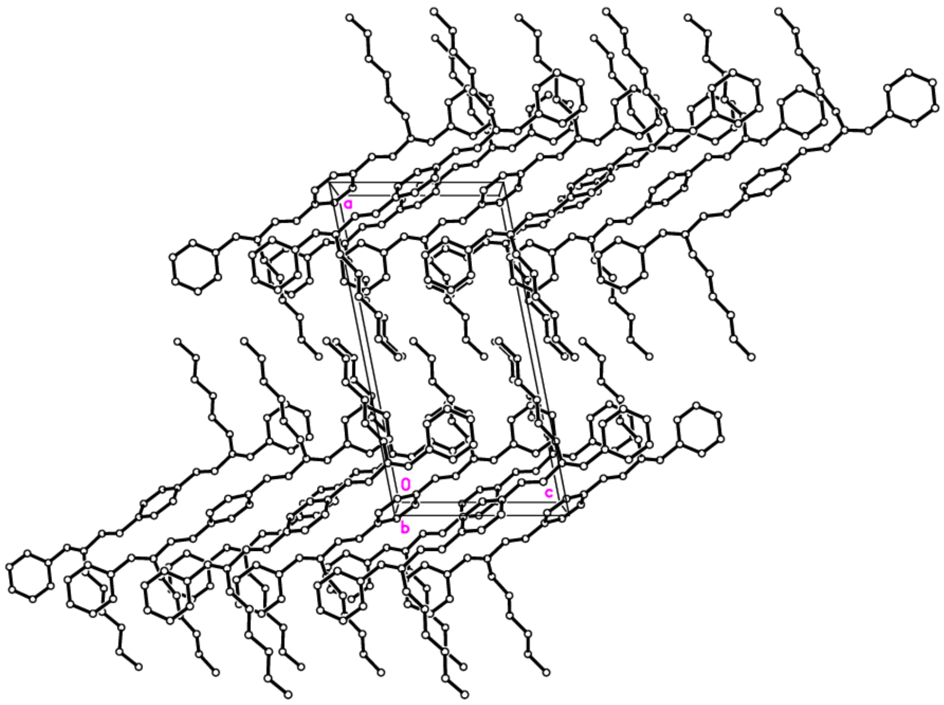

Figure S10. Packing plot of **7m** viewed parallel to *b*. The two terminal C atoms in the C_6 chain interdigitate with other nearby molecules.

For **9c**: A whole molecule forms the asymmetric unit. Data collected at 200 K, rather than 100 K, because crystals shattered at the lower temperature. Since there is no solvent of crystallisation, this suggests a significant phase change below 200K. There was some degree of ambiguity in the space group. An attempt was made to solve the structure in the highest symmetry, centro-symmetric, space group, *Pnma*. The best *R* factor that could be achieved was *ca*. 6.5% and modelling the disordered CF_3 groups across the mirror plane was problematic. A significantly better result was obtained in non-centrosymmetric space group $Pna2_1$. Here an *R* factor of *ca*. 4% was achieved with a much more satisfactory modelling of the disordered CF_3 groups. The absolute structure could not be reliably determined. The three rings are co-planar. The CF_3 groups were both modelled with the F atoms split over two sets of positions. For that at C(11) the major component occupancy was 51.1(14)%, while that at C(18) was 56.8(12)%. In both cases restraints were applied to geometric and anisotropic displacement parameters.

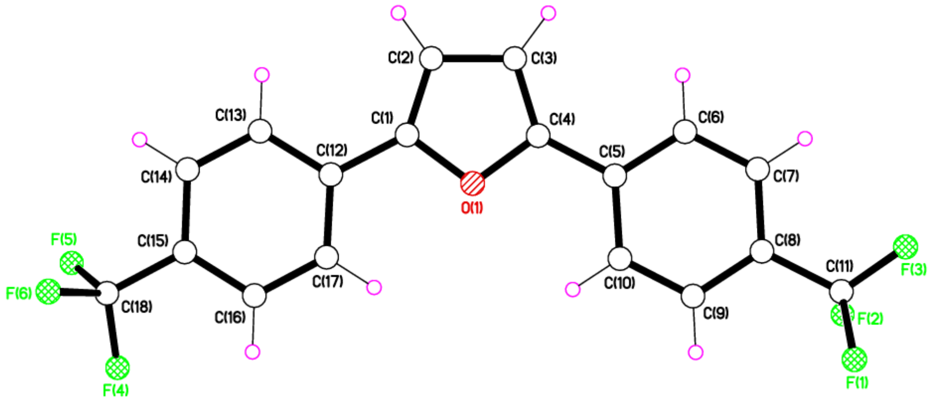

Figure S11. Single molecule of **9c**.

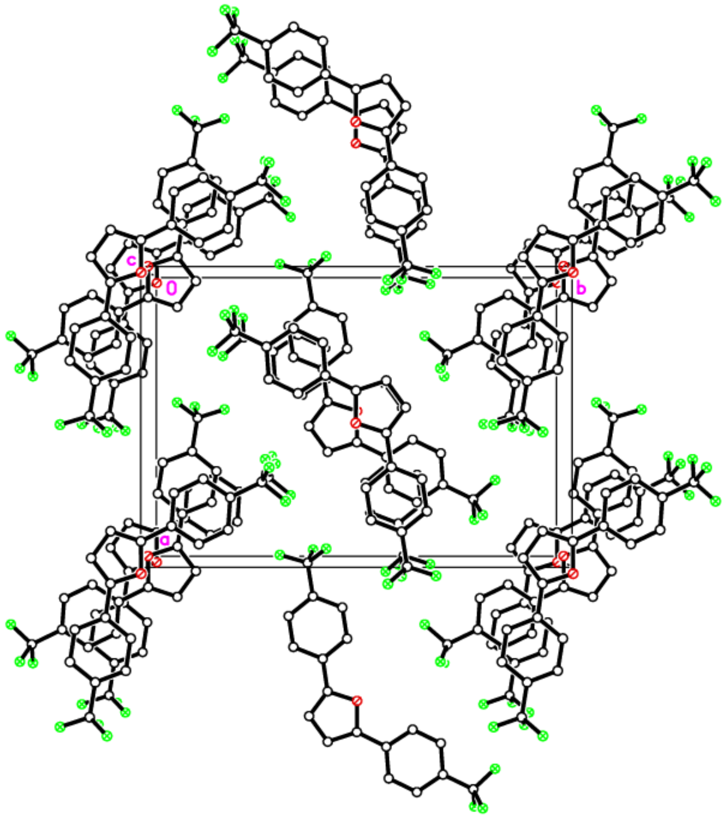

Figure S12. Packing plot of **9c** viewed parallel to *c*. Molecules form stacks parallel to *c* with neighbouring molecules rotated 180□and separated by *c*/2 = 3.40 Å, with substantially slipped π···π overlap, but avoiding $CF_3 \cdots CF_3$ clashes. There are no halogen-halogen interactions.

For **9i**: Due to the C_2 symmetry, and half the molecule being unique, the Br and Me groups were modelled as exactly 50/50 disordered. The (Ar)C–Me bond length was restrained. The

Me H atoms were clearly shown in electron density difference maps prior to being modelled as a constrained group. The twist angle about C(2)–C(3) is 11.53(6)□, so the C_6H_4 aromatic rings are not co-planar with the central ring.

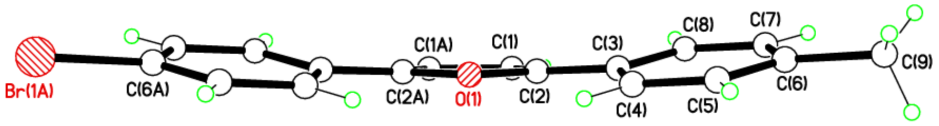

Figure S13. Side-on view of **9i**, showing molecule is not completely planar.

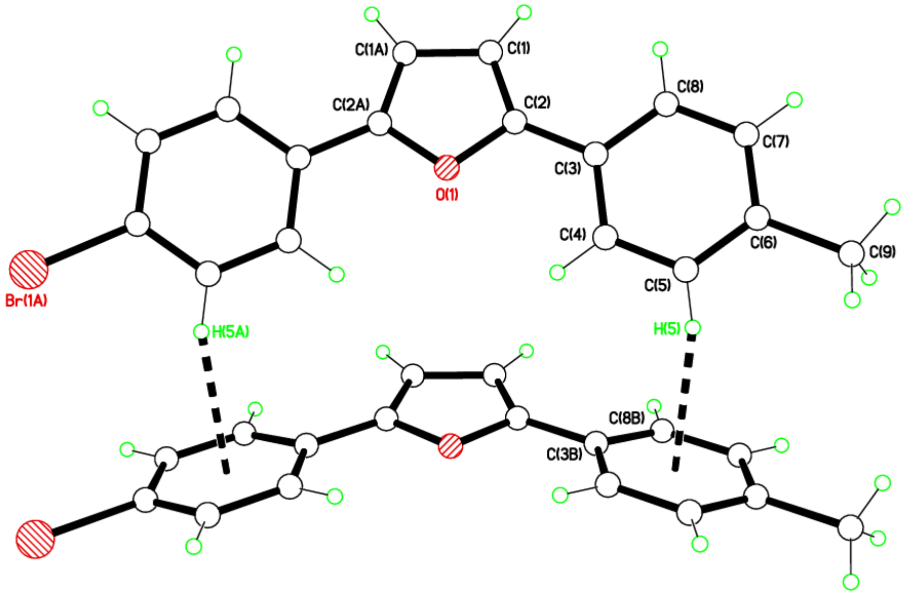

Figure S14. C–H···π interactions in **9i** linking molecules in the *c* direction with an H(5) to ring centroid distance of 2.66 Å.

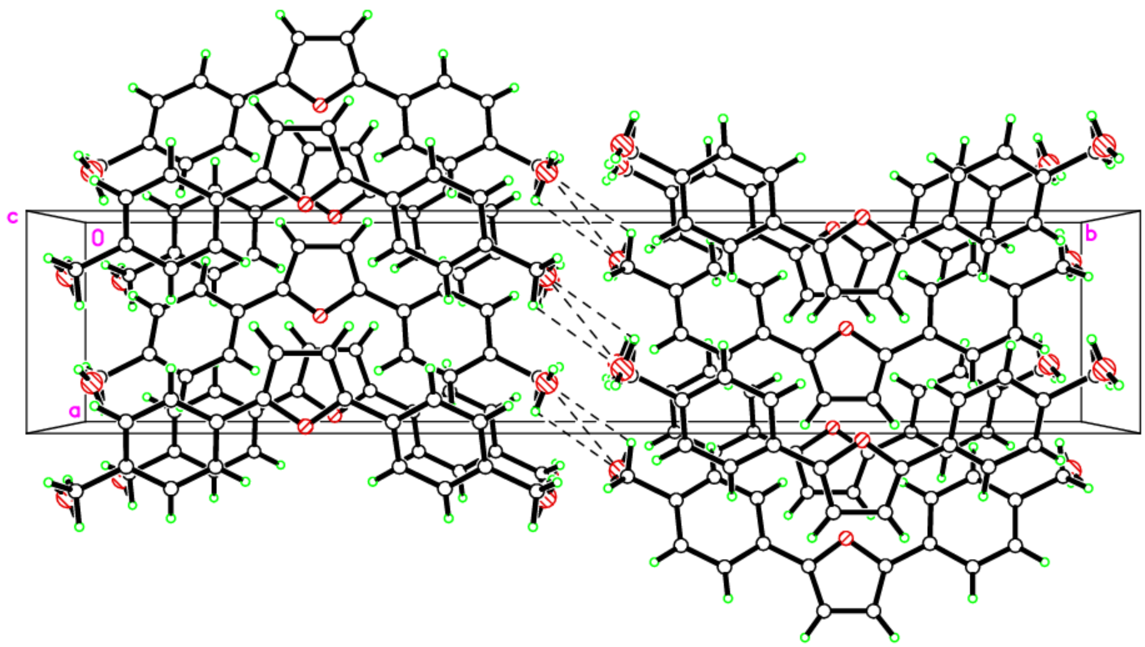

Figure S15. Packing plot of **9i** viewed parallel to *c*. Either Br(1)···Br(1') =3.377 Å or Br(1)···H(9A') = 3.05 Å intermolecular interactions giving sinusoidal chains along *b*. This depends on the exact groups in close proximity considering the disorder.

Table S3

*Experimental details for **7a**.*

Crystal data	
Chemical formula	$C_{16}H_{12}Br_2$
M_r	364.08
Crystal system, space group	Orthorhombic, *Pccn*
Temperature (K)	100
a, *b*, *c* (Å)	32.074 (2), 7.2091 (5), 5.8863 (3)
V (Å^3)	1361.06 (14)
Z	4
Radiation type	Mo $K\alpha$
□ (mm^{-1})	5.93
Crystal size (mm^3)	0.24 × 0.22 × 0.02
Data collection	
Diffractometer	Rigaku FRE+ equipped with Arc)Sec VHF Varimax confocal mirrors and an UG2 goniometer and HyPix 6000HE detector
Absorption correction	Analytical. *CrysAlis PRO* 1.171.42.59a (Rigaku Oxford Diffraction, 2022). Analytical numeric absorption correction using a multifaceted crystal model based on expressions derived by R.C. Clark & J.S. Reid. (Clark, R. C. & Reid, J. S. (1995). Acta Cryst. A51, 887-897) Empirical absorption correction using spherical harmonics, implemented in SCALE3 ABSPACK scaling algorithm.
T_{min}, T_{max}	0.856, 0.971
No. of measured, independent and observed [$I > 2\sigma(I)$] reflections	18578, 2049, 1660
R_{int}	0.060
$(\sin \theta/\lambda)_{max}$ (Å^{-1})	0.714
Refinement	
$R[F^2 > 2\sigma(F^2)]$, $wR(F^2)$, S	0.071, 0.194, 1.22
No. of reflections	2049
No. of parameters	82
H-atom treatment	H-atom parameters constrained
	$w = 1/[\sigma^2(F_o^2) + (0.0411P)^2 + 30.560P]$ where $P = (F_o^2 + 2F_c^2)/3$
$\Delta\rangle_{max}$, $\Delta\rangle_{min}$ (e Å^{-3})	3.30, -2.17

Computer programs: *CrysAlis PRO* 1.171.42.59a (Rigaku OD, 2022), SHELXT-2018/2 (Sheldrick, 2015), *SHELXL2018*/3 (Sheldrick, 2018), Bruker *SHELXTL*.

Table S4

*Experimental details for **7i**.*

Crystal data	
Chemical formula	$C_{16}H_{12}BrCl$
M_r	319.62
Crystal system, space group	Monoclinic, $P2_1/c$
Temperature (K)	106
a, b, c (Å)	12.1007 (5), 3.94582 (15), 13.7282 (5)
β (°)	91.329 (3)
V (Å^3)	655.31 (4)
Z	2
Radiation type	Mo $K\alpha$
[illegible] (mm^{-1})	3.32
Crystal size (mm^3)	0.28 × 0.07 × 0.02
Data collection	
Diffractometer	Rigaku FRE+ equipped with Arc)Sec VHF Varimax confocal mirrors and an UG2 goniometer and HyPix 6000HE detector
Absorption correction	Multi-scan. *CrysAlis PRO* 1.171.42.51a (Rigaku Oxford Diffraction, 2022). Empirical absorption correction using spherical harmonics, implemented in SCALE3 ABSPACK scaling algorithm.
T_{min}, T_{max}	0.618, 1.000
No. of measured, independent and observed [$I > 2\sigma(I)$] reflections	24806, 1705, 1595
R_{int}	0.045
$(\sin \theta/\lambda)_{max}$ (Å^{-1})	0.676
Refinement	
$R[F^2 > 2\sigma(F^2)]$, $wR(F^2)$, S	0.028, 0.064, 1.17
No. of reflections	1705
No. of parameters	91
No. of restraints	30
H-atom treatment	H-atom parameters constrained
$\Delta\rangle_{max}$, $\Delta\rangle_{min}$ (e Å^{-3})	0.45, -0.30

Computer programs: *CrysAlis PRO* 1.171.42.51a (Rigaku OD, 2022), SHELXT-2018/2 (Sheldrick, 2015), *SHELXL2018*/3 (Sheldrick, 2018), Bruker *SHELXTL*.

Table S5

Experimental details for 7m.

Crystal data	
Chemical formula	$C_{38}H_{46}$
M_r	502.75
Crystal system, space group	Monoclinic, $P2_1/c$
Temperature (K)	100
a, b, c (Å)	18.6524 (3), 8.60695 (18), 9.57890 (18)
β (°)	101.1633 (16)
V ($Å^3$)	1508.70 (5)
Z	2
Radiation type	Cu $K\alpha$
□ (mm^{-1})	0.46
Crystal size (mm^3)	0.47 × 0.26 × 0.10
Data collection	
Diffractometer	Rigaku 007HF. diffractometer with HF Varimax confocal mirrors, an UG2 goniometer and HyPix 6000HE detector
Absorption correction	Analytical. *CrysAlis PRO* 1.171.42.59a (Rigaku Oxford Diffraction, 2022). Analytical numeric absorption correction using a multifaceted crystal model based on expressions derived by R.C. Clark & J.S. Reid. (Clark, R. C. & Reid, J. S. (1995). Acta Cryst. A51, 887-897) Empirical absorption correction using spherical harmonics, implemented in SCALE3 ABSPACK scaling algorithm.
T_{min}, T_{max}	0.936, 0.979
No. of measured, independent and observed [$I > 2\sigma(I)$] reflections	16036, 3129, 2893
R_{int}	0.043
$(\sin \theta/\lambda)_{max}$ ($Å^{-1}$)	0.631
Refinement	
$R[F^2 > 2\sigma(F^2)]$, $wR(F^2)$, S	0.038, 0.103, 1.06
No. of reflections	3129
No. of parameters	265
H-atom treatment	All H-atom parameters refined
$\Delta\rangle_{max}$, $\Delta\rangle_{min}$ (e $Å^{-3}$)	0.27, -0.16

Computer programs: *CrysAlis PRO* 1.171.42.59a (Rigaku OD, 2022), SHELXT 2018/2 (Sheldrick, 2018), *SHELXL2018*/3 (Sheldrick, 2018), Bruker *SHELXTL*.

Table S6

*Experimental details for **9c**.*

Crystal data	
Chemical formula	$C_{18}H_{10}F_6O$
M_r	356.26
Crystal system, space group	Orthorhombic, $Pna2_1$
Temperature (K)	200
a, *b*, *c* (Å)	12.7746 (4), 18.3157 (7), 6.80562 (18)
V (Å^3)	1592.35 (9)
Z	4
Radiation type	Cu $K\alpha$
□ (mm^{-1})	1.24
Crystal size (mm^3)	0.24 × 0.05 × 0.04
Data collection	
Diffractometer	Rigaku 007HF diffractometer with HF Varimax confocal mirrors, an UG2 goniometer and HyPix 6000HE detector
Absorption correction	Multi-scan. *CrysAlis PRO* 1.171.42.51a (Rigaku Oxford Diffraction, 2022). Empirical absorption correction using spherical harmonics, implemented in SCALE3 ABSPACK scaling algorithm.
T_{min}, T_{max}	0.486, 1.000
No. of measured, independent and observed [$I > 2\sigma(I)$] reflections	13935, 2328, 1885
R_{int}	0.032
$(\sin \theta/\lambda)_{max}$ (Å^{-1})	0.610
Refinement	
$R[F^2 > 2\sigma(F^2)]$, $wR(F^2)$, S	0.041, 0.122, 1.04
No. of reflections	2328
No. of parameters	282
No. of restraints	369
H-atom treatment	H-atom parameters constrained
$\Delta\rangle_{max}$, $\Delta\rangle_{min}$ (e Å^{-3})	0.11, -0.16
Absolute structure	Classical Flack method preferred over Parsons because s.u. lower.
Absolute structure parameter	0.2 (5)

Computer programs: *CrysAlis PRO* 1.171.42.51a (Rigaku OD, 2022), SHELXT 2018/2 (Sheldrick, 2018), *SHELXL2018*/3 (Sheldrick, 2018), Bruker *SHELXTL*.

Table S7

*Experimental details for **9i**.*

Crystal data	
Chemical formula	$C_{17}H_{13}BrO$
M_r	313.18
Crystal system, space group	Orthorhombic, *Pnma*
Temperature (K)	101
a, *b*, *c* (Å)	6.0754 (2), 30.383 (1), 7.2266 (3)
V ($Å^3$)	1333.95 (8)
Z	4
Radiation type	Mo *K*α
□ (mm^{-1})	3.07
Crystal size (mm^3)	0.43 × 0.22 × 0.11
Data collection	
Diffractometer	Rigaku FRE+ equipped with Arc)Sec VHF Varimax confocal mirrors and an UG2 goniometer and HyPix 6000HE detector
Absorption correction	Analytical. *CrysAlis PRO* 1.171.42.59a (Rigaku Oxford Diffraction, 2022). Analytical numeric absorption correction using a multifaceted crystal model based on expressions derived by R.C. Clark & J.S. Reid. (Clark, R. C. & Reid, J. S. (1995). Acta Cryst. A51, 887-897). Empirical absorption correction using spherical harmonics, implemented in SCALE3 ABSPACK scaling algorithm.
T_{min}, T_{max}	0.809, 0.936
No. of measured, independent and observed [$I > 2\sigma(I)$] reflections	16349, 2438, 2001
R_{int}	0.031
$(\sin \theta/\lambda)_{max}$ ($Å^{-1}$)	0.779
Refinement	
$R[F^2 > 2\sigma(F^2)]$, $wR(F^2)$, *S*	0.032, 0.077, 1.06
No. of reflections	2438
No. of parameters	98
No. of restraints	26
H-atom treatment	H-atom parameters constrained
$\Delta\rangle_{max}$, $\Delta\rangle_{min}$ (e $Å^{-3}$)	0.42, -0.25

Computer programs: *CrysAlis PRO* 1.171.42.59a (Rigaku OD, 2022), SHELXT 2018/2 (Sheldrick, 2018), *SHELXL2018*/3 (Sheldrick, 2018), Bruker *SHELXTL*.

References.

(1) Liu, Y.; Cai, L.; Xu, S.; Pu, W.; Tao, X. Palladium-Catalyzed Hydroformylation of Terminal Arylacetylenes with Glyoxylic Acid. *Chem. Commun.* **2018**, *54*, 2166–2168. https://doi.org/10.1039/C7CC09629A.

(2) Wang, M.-M.; Ning, X.-S.; Qu, J.-P.; Kang, Y.-B. Dehydrogenative Synthesis of Linear α,β-Unsaturated Aldehydes with Oxygen at Room Temperature Enabled by t BuONO. *ACS Catal.* **2017**, *7* (6), 4000–4003. https://doi.org/10.1021/acscatal.7b01008.

(3) Saifuddin, M.; Guo, C.; Biewenga, L.; Saravanan, T.; Charnock, S. J.; Poelarends, G. J. Enantioselective Aldol Addition of Acetaldehyde to Aromatic Aldehydes Catalyzed by Proline-Based Carboligases. *ACS Catal*. **2020**, *10* (4), 2522–2527. https://doi.org/10.1021/acscatal.0c00039.

(4) Jiang, T.-S.; Li, J.-H. Palladium-Catalyzed Oxidative Tandem Reaction of Allylamines with Aryl Halides Leading to α,β-Unsaturated Aldehydes. *Chem. Communn.* **2009**, 7236. https://doi.org/10.1039/b917782e.

(5) Ren, S.; Seki, T.; Necas, D.; Shimizu, H.; Nakajima, K.; Kanno, K.; Song, Z.; Takahashi, T. Selective Dimerization of Aryl-Substituted Terminal Alkynes on Bis(Indenyl)Zirconocene Derivatives. *Chem. Lett.* **2011**, *40*, 1443–1444. https://doi.org/10.1246/cl.2011.1443.

(6) Hintermann, L.; Schmitz, M.; Chen, Y. A Direct Synthesis of Symmetrical (*E*,*E*)-1,4-Diaryl-1,3-Butadienes by Wenkert Arylation of Thiophene. *Adv. Synth. Catal.* **2010**, *352*, 2411–2415. https://doi.org/10.1002/adsc.201000350.

(7) Babudri, F.; Farinola, G.; Naso, F.; Ragni, R.; Spina, G. A Novel Stereoselective Synthesis of Symmetrical (1*E* ,3*E*)-1,4-Diarylbuta-1,3-Dienes. *Synthesis* **2007**, *2007*, 3088–3092. https://doi.org/10.1055/s-2007-983833.

(8) Denmark, S. E.; Tymonko, S. A. Sequential Cross-Coupling of 1,4-Bissilylbutadienes: Synthesis of Unsymmetrical 1,4-Disubstituted 1,3-Butadienes. *J. Am. Chem. Soc.* **2005**, *127*, 8004–8005. https://doi.org/10.1021/ja0518373.

(9) Zhang, S.; Zhang, D.; Liebeskind, L. S. Ambient Temperature, Ullmann-like Reductive Coupling of Aryl, Heteroaryl, and Alkenyl Halides. *J. Org. Chem.* **1997**, *62*, 2312–2313. https://doi.org/10.1021/jo9700078.

(10) Legendre, S. V. A.-M.; Sumby, C. J.; Karton, A.; Greatrex, B. W. Desymmetrization and Kinetic Resolution of Endoperoxides Using a Bifunctional Organocatalyst. *J. Org. Chem.* **2023**, *88*, 11444–11449. https://doi.org/10.1021/acs.joc.3c00278.

(11) Mitsudo, T.; Fischetti, W.; Heck, R. F. Palladium-Catalyzed Syntheses of Aryl Polyenes. *J. Org. Chem.* **1984**, *49*, 1640–1646. https://doi.org/10.1021/jo00183a029.

(12) Weber, M.; Singh, F. v.; Vieira, A. S.; Stefani, H. A.; Paixão, M. W. Palladium-Catalyzed Oxidative Homocoupling of Potassium Alkenyltrifluoroborates: Synthesis of Symmetrical 1,3-Dienes. *Tetrahedron Lett*. **2009**, *50*, 4324–4327. https://doi.org/10.1016/j.tetlet.2009.04.127.

(13) Wang, J.-X.; Wang, K.; Zhao, L.; Li, H.; Fu, Y.; Hu, Y. Palladium-Catalyzed Stereoselective Synthesis of (E)-Stilbenes via Organozinc Reagents and Carbonyl Compounds. *Adv. Synth. Catal.* **2006**, *348*, 1262–1270. https://doi.org/10.1002/adsc.200606016.

NMR spectra of prepared compounds - benzylacrylaldehyde

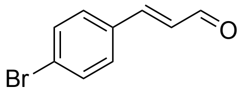

12a ^{1}H NMR ($CDCl_3$, 500 MHz)

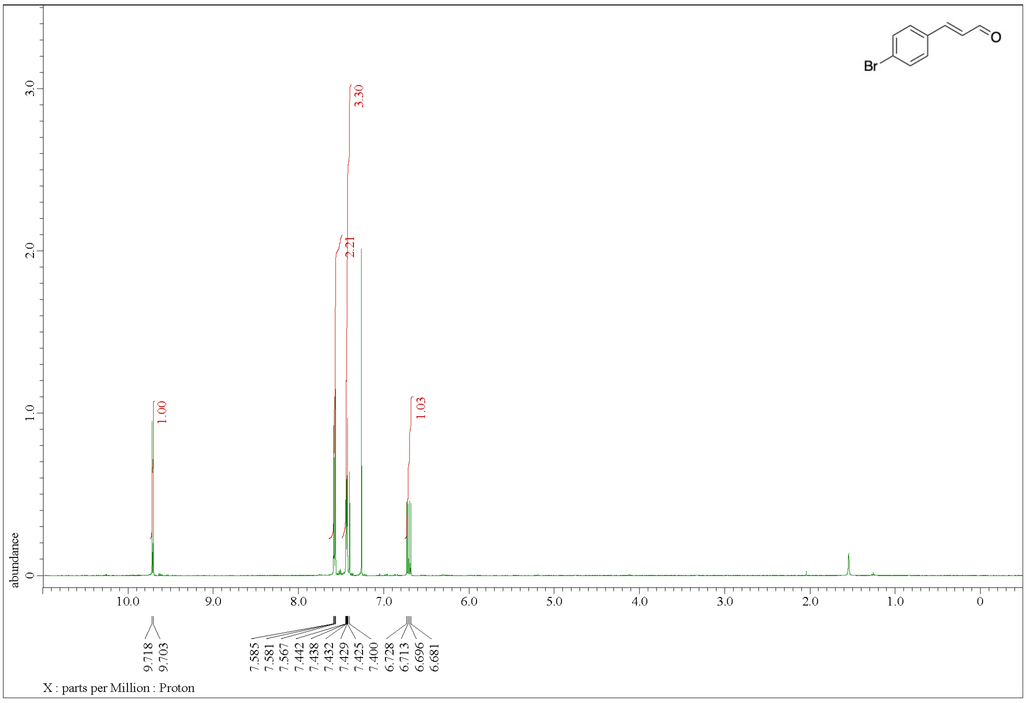

12a ^{13}C NMR ($CDCl_3$, 126 MHz)

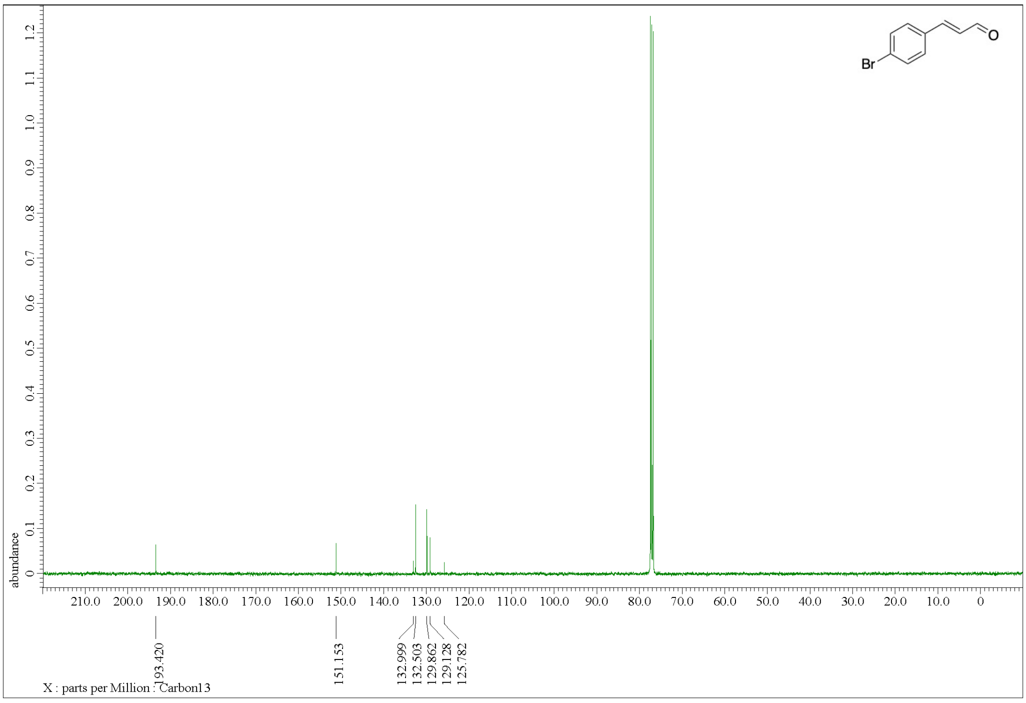

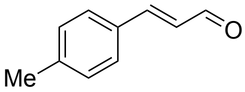

12b ^{1}H NMR ($CDCl_3$, 400 MHz)

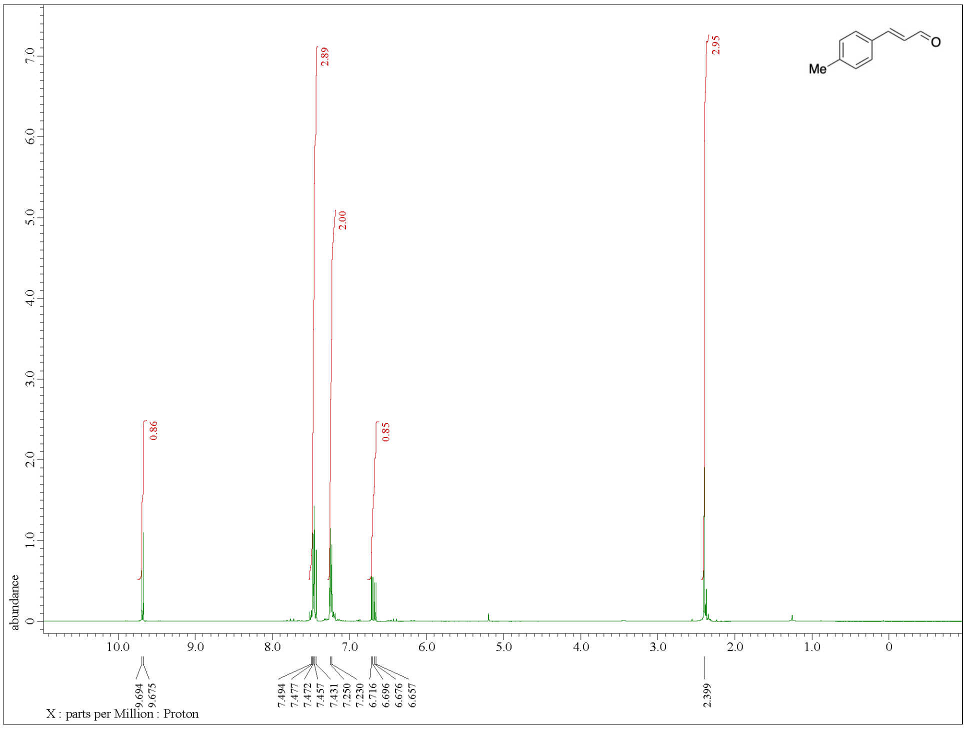

12b ^{13}C NMR ($CDCl_3$, 100 MHz)

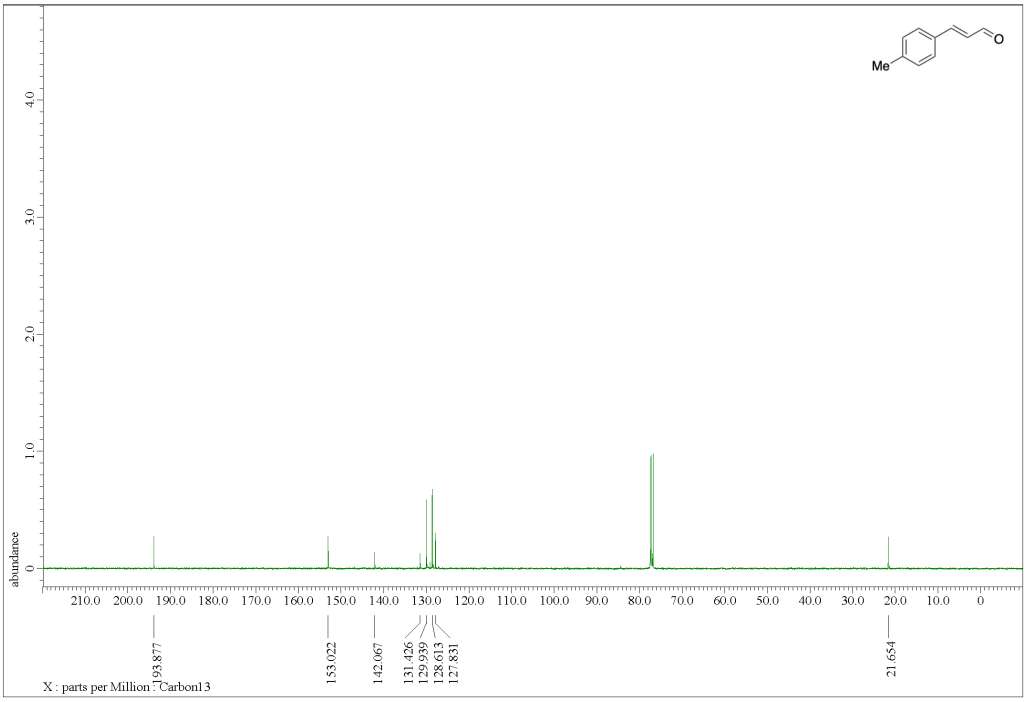

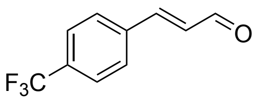

12c ^{1}H NMR ($CDCl_3$, 400 MHz)

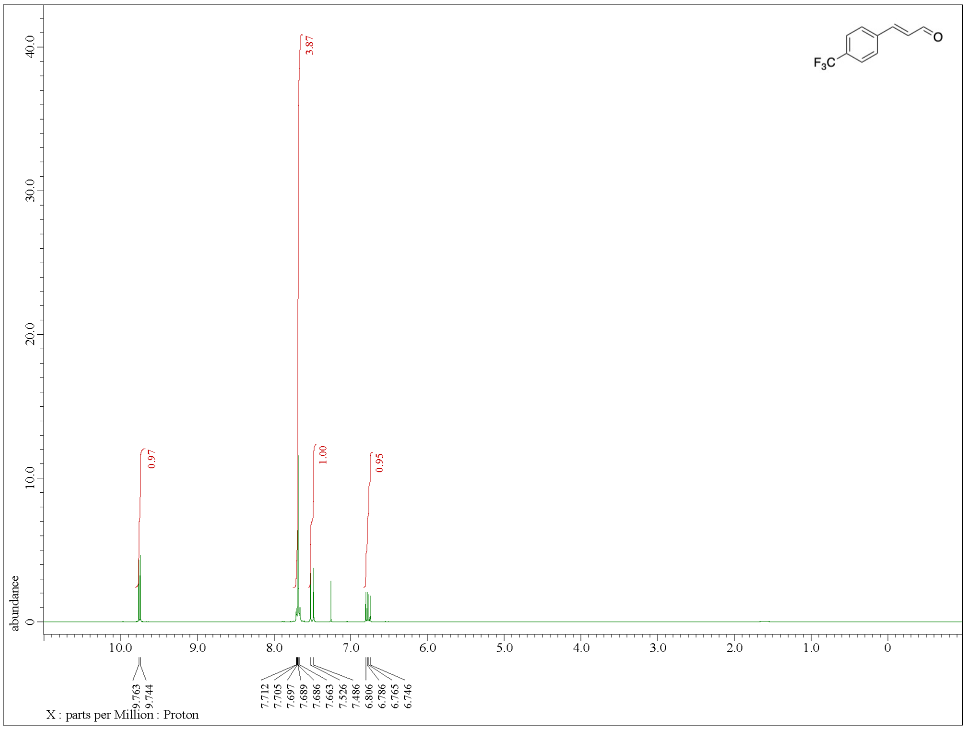

12c ^{13}C NMR ($CDCl_3$, 100 MHz)

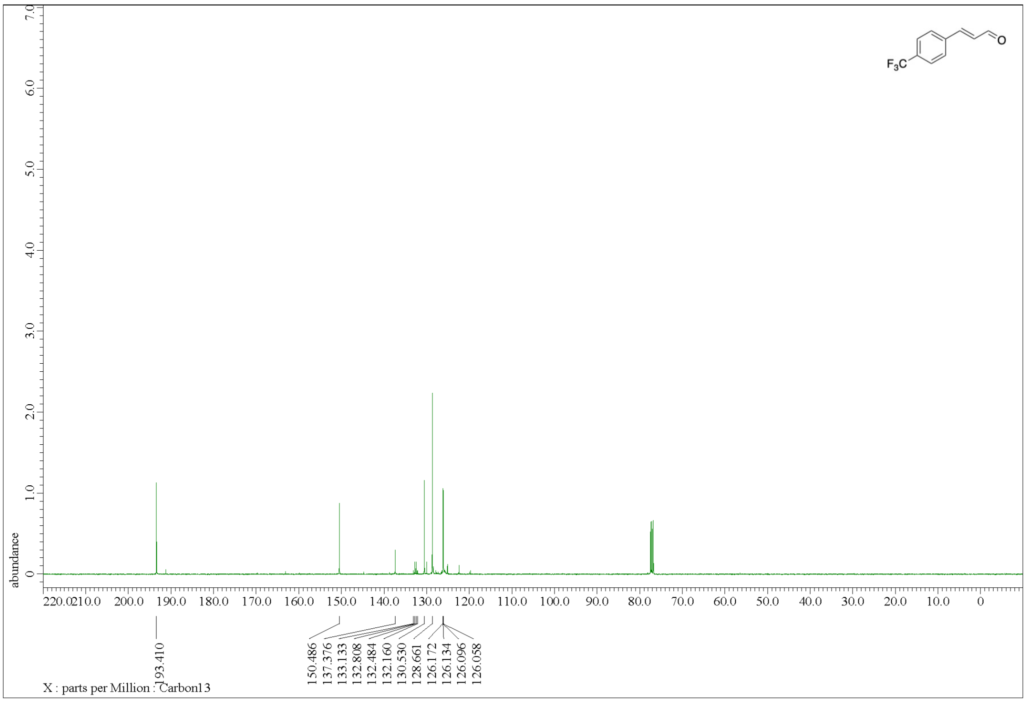

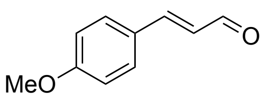

12d ^{1}H NMR ($CDCl_3$, 400 MHz)

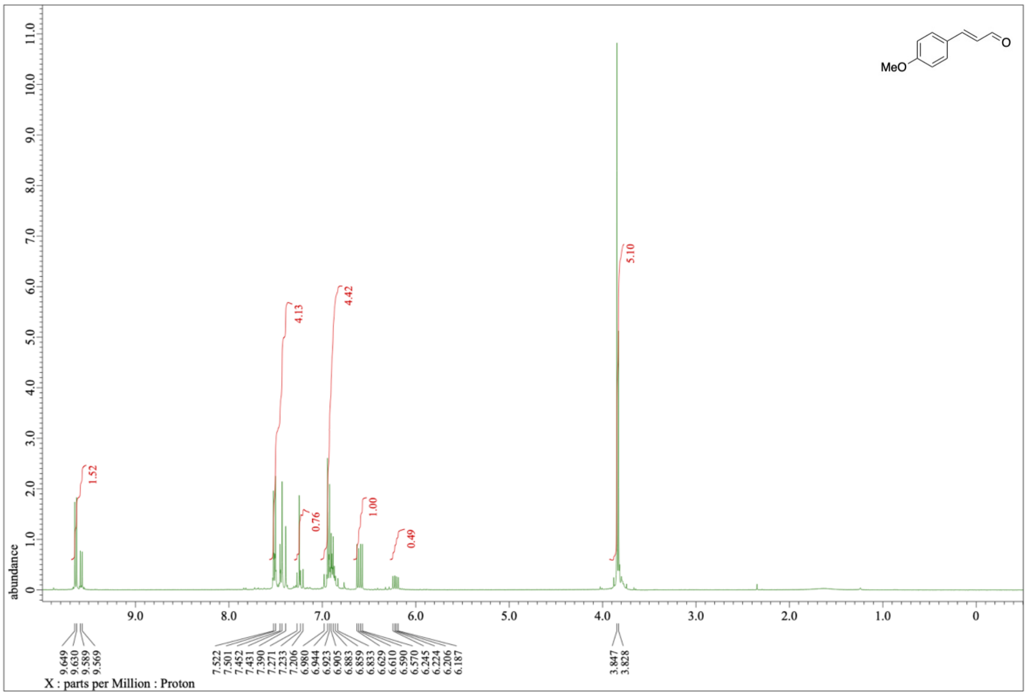

12d ^{13}C NMR ($CDCl_3$, 100 MHz)

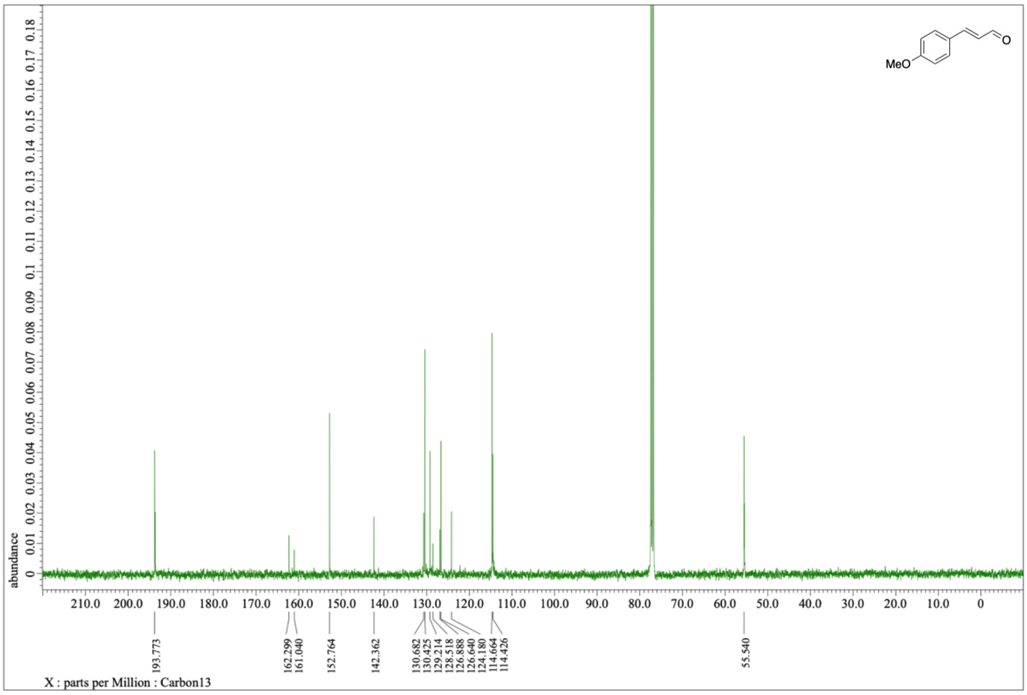

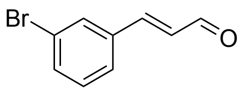

12e ^{1}H NMR ($CDCl_3$, 500 MHz)

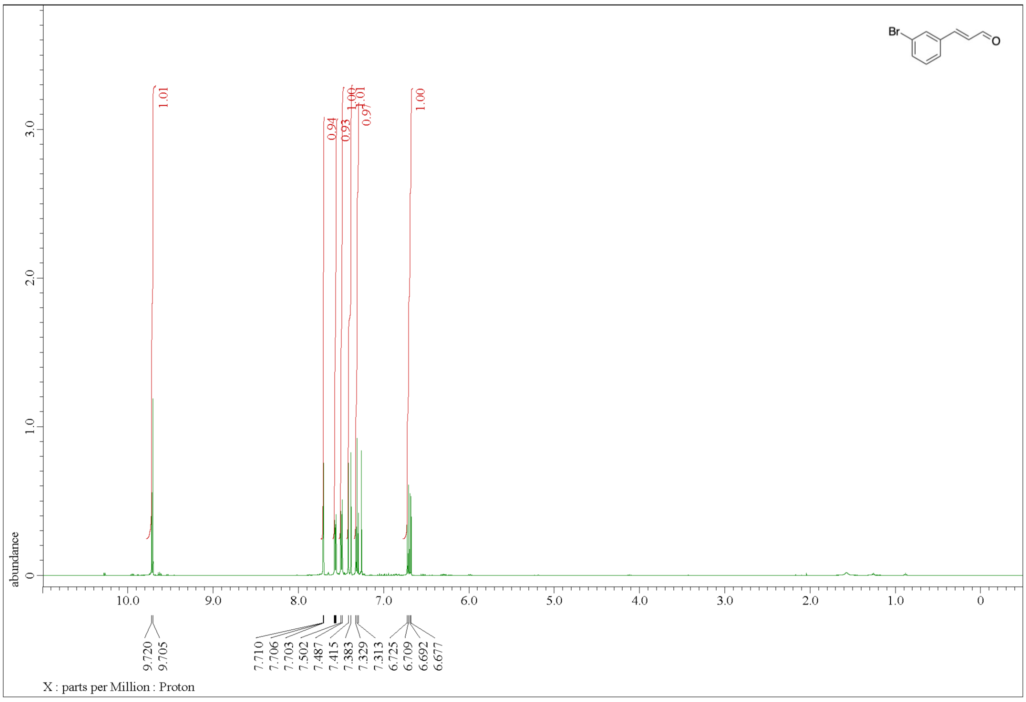

12e ^{13}C NMR ($CDCl_3$, 126 MHz)

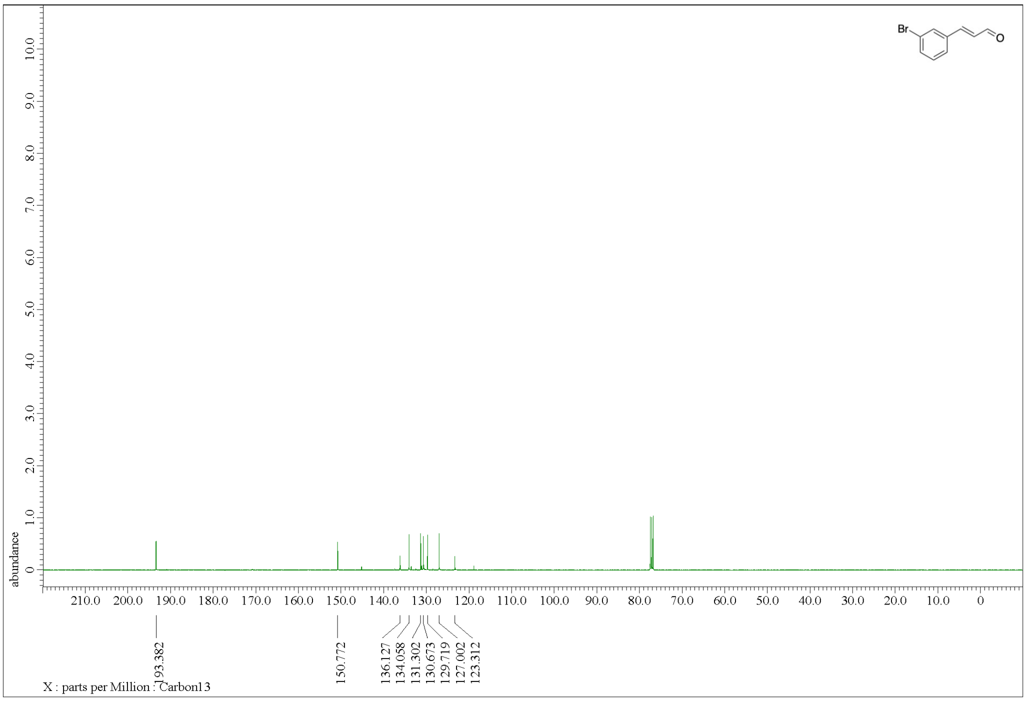

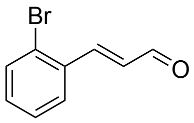

12f ^{1}H NMR ($CDCl_3$, 400 MHz)

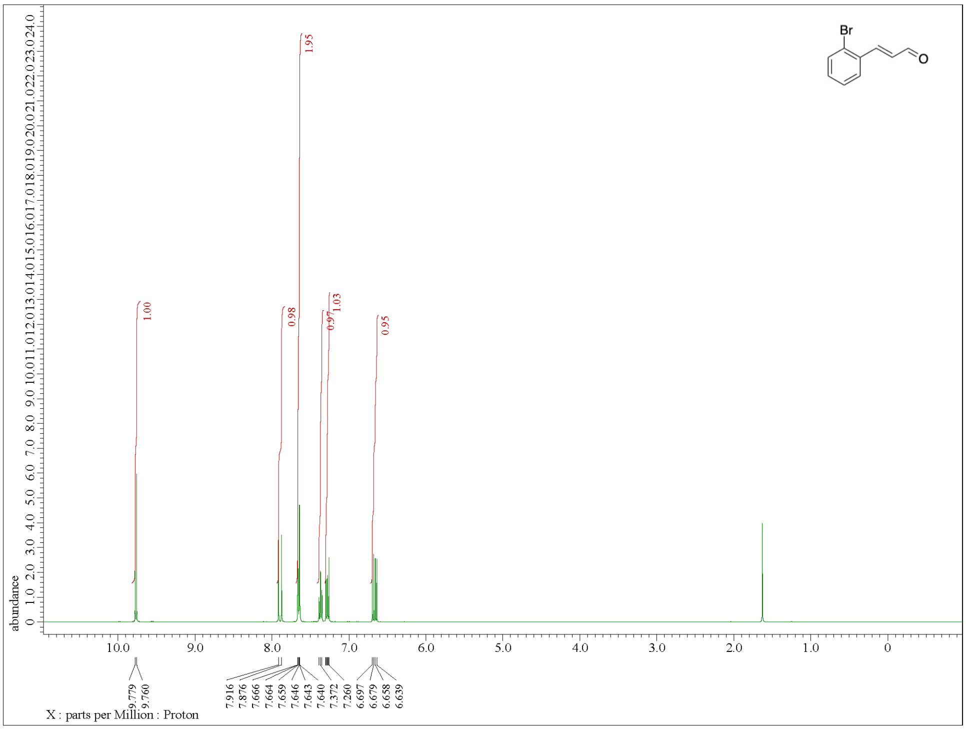

12f ^{13}C NMR ($CDCl_3$, 100 MHz)

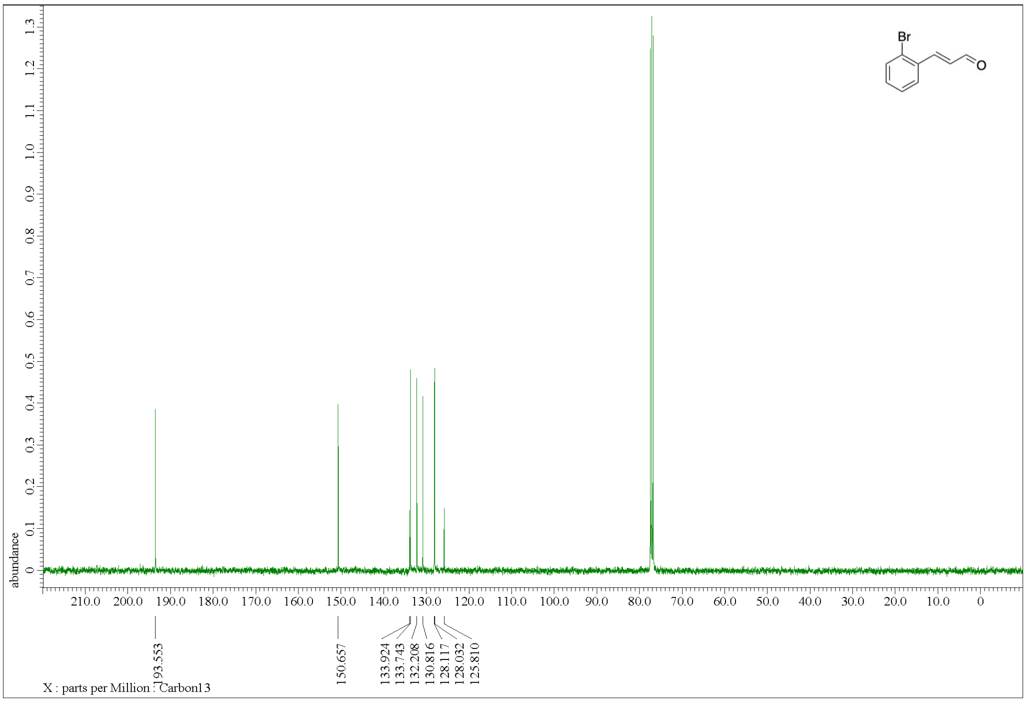

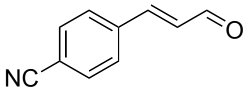

12g ^{1}H NMR ($CDCl_3$, 400 MHz)

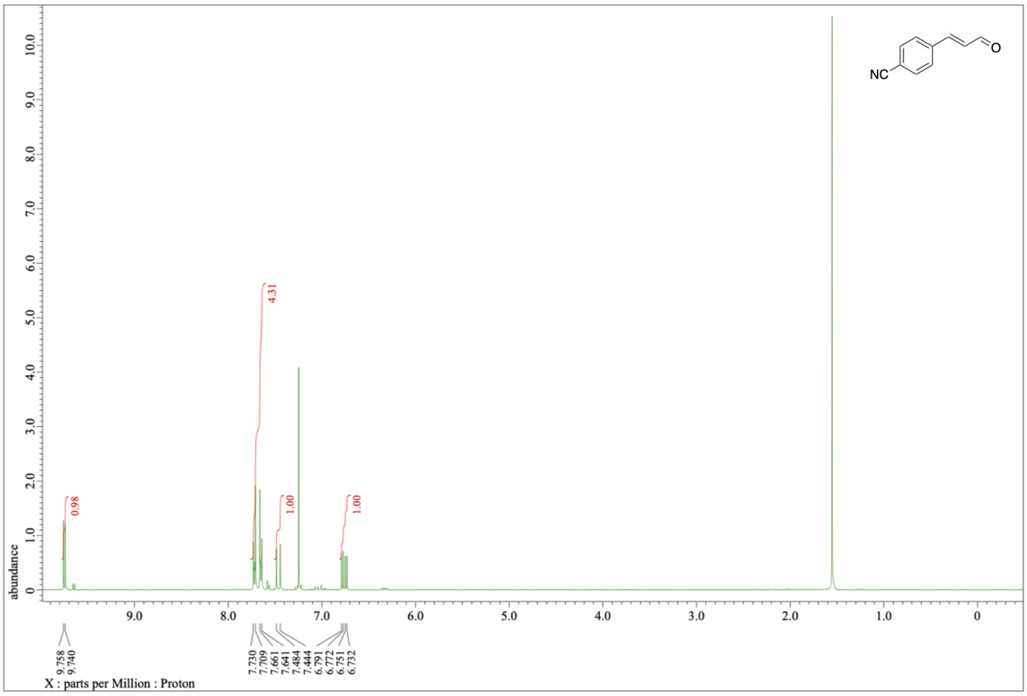

12g ^{13}C NMR ($CDCl_3$, 100 MHz)

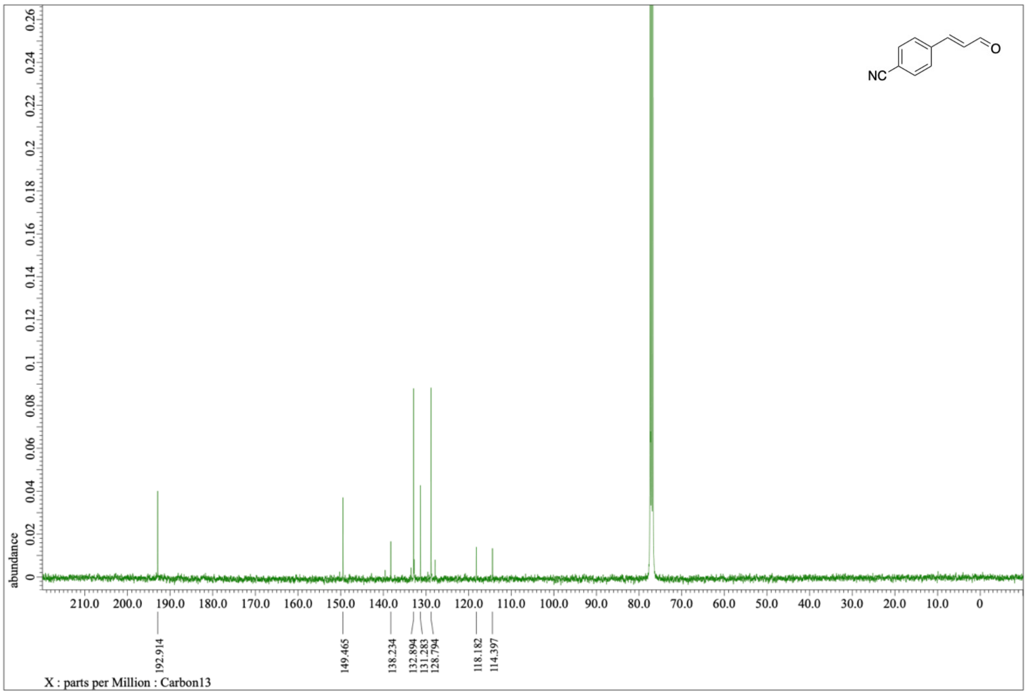

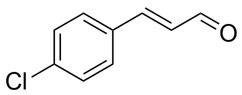

12h ^{1}H NMR ($CDCl_3$, 400 MHz)

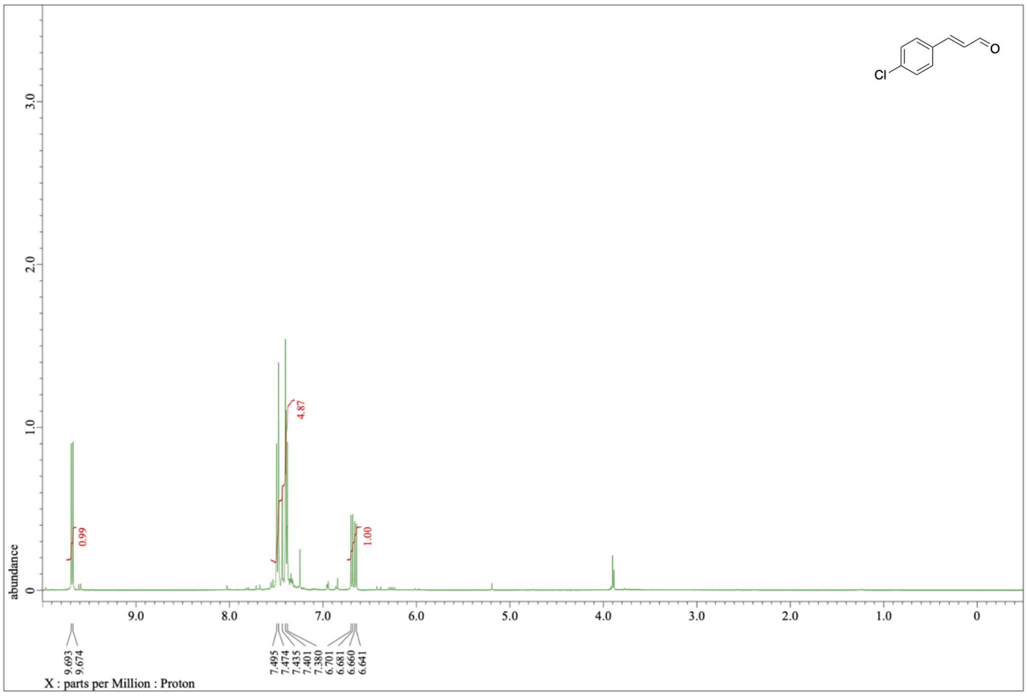

12h ^{13}C NMR ($CDCl_3$, 100 MHz)

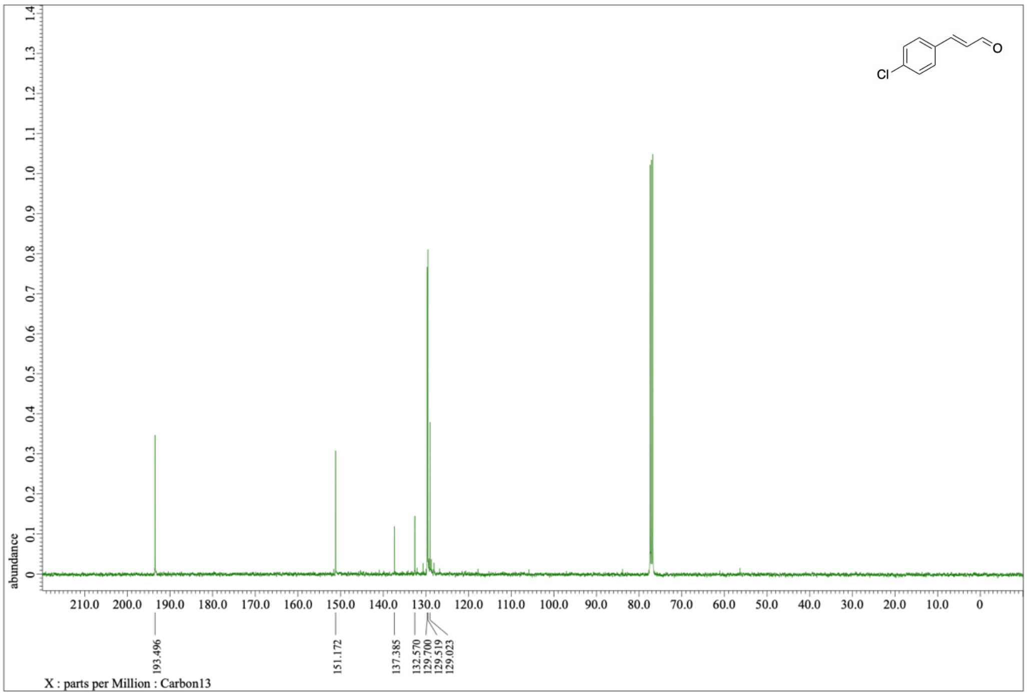

NMR spectra of prepared compounds – 1,3-dienes

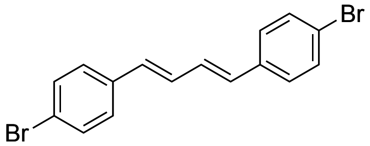

7a ^{1}H NMR ($CDCl_3$, 400 MHz)

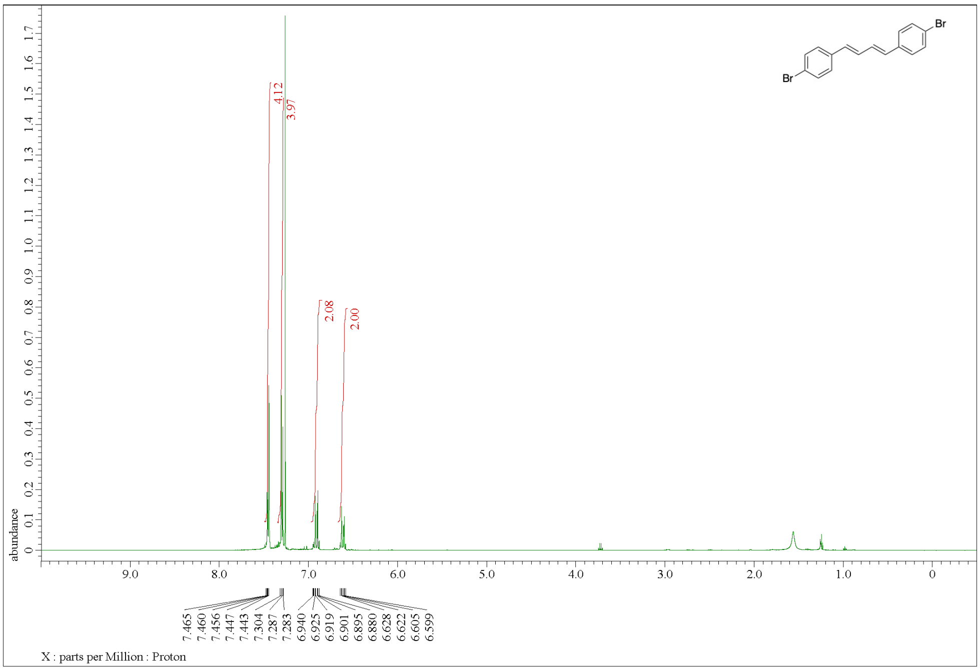

7a ^{13}C NMR ($CDCl_3$, 100 MHz)

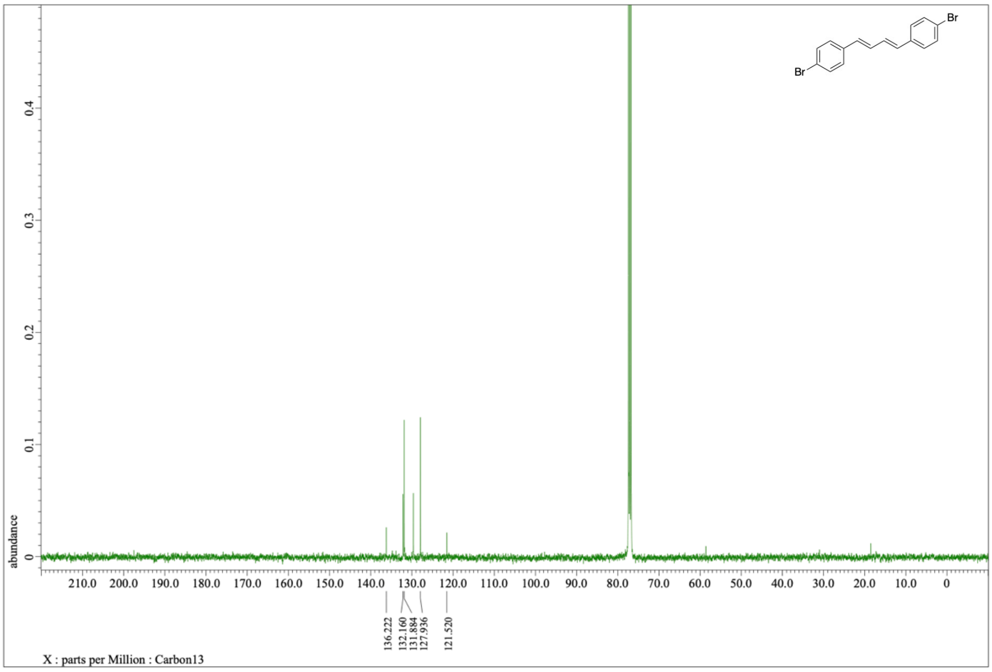

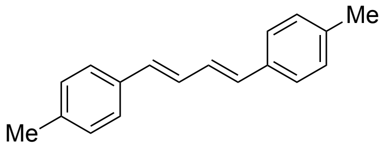

7b ^{1}H NMR ($CDCl_3$, 500 MHz)

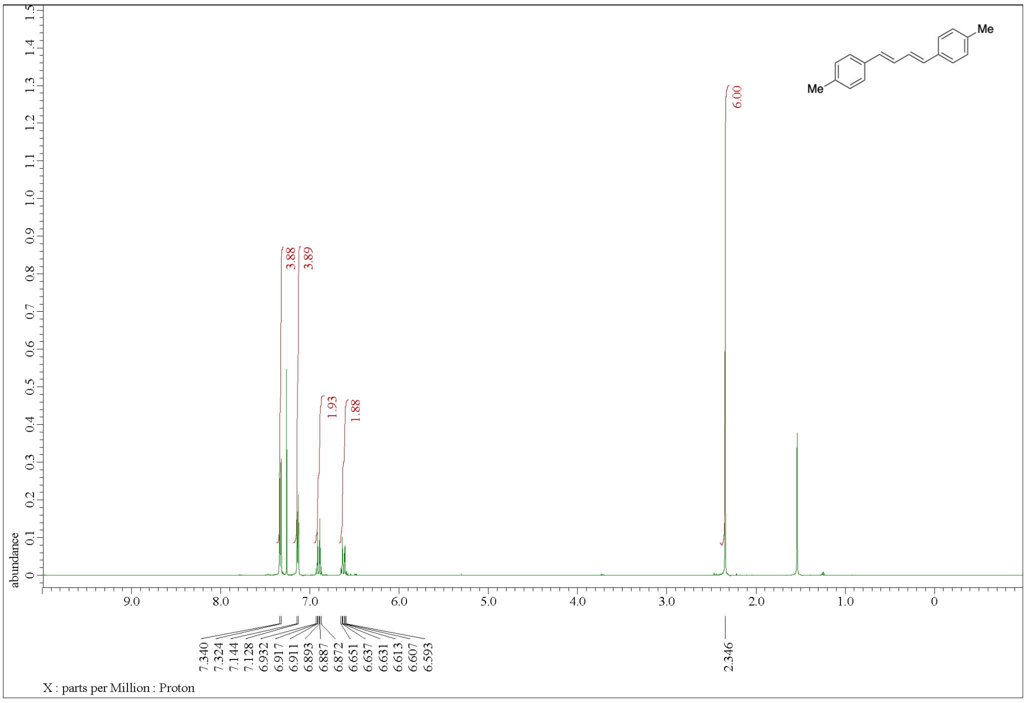

7b ^{13}C NMR ($CDCl_3$, 126 MHz)

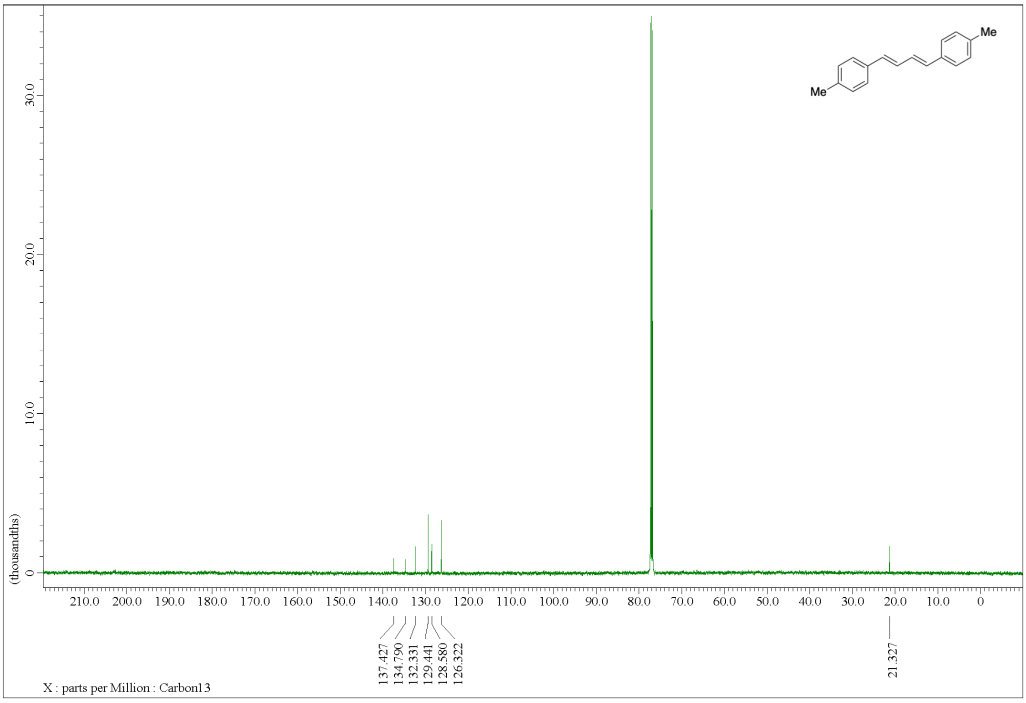

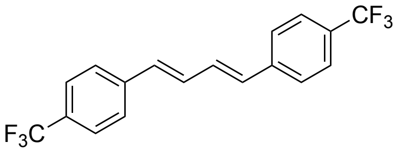

7c ^{1}H NMR ($CDCl_3$, 400 MHz)

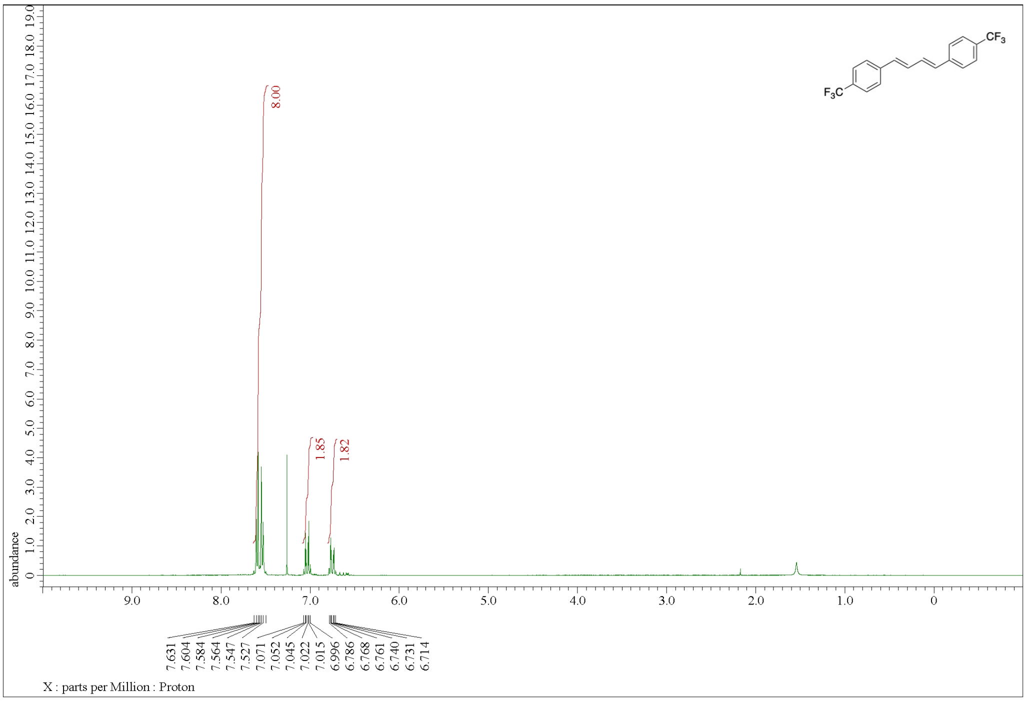

7c ^{13}C NMR ($CDCl_3$, 100 MHz)

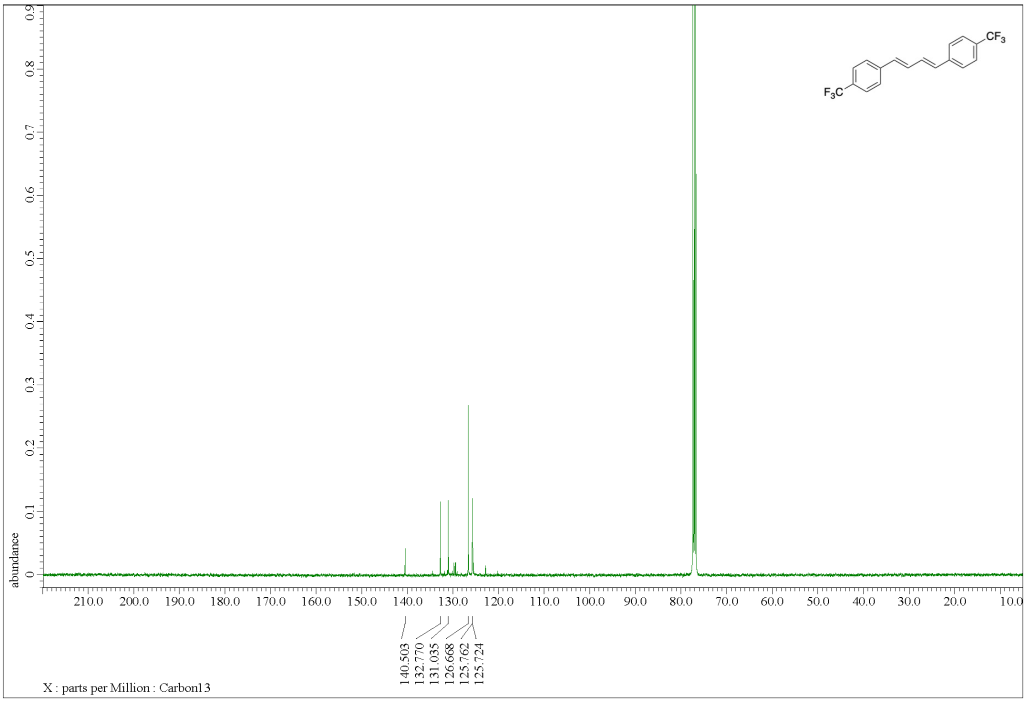

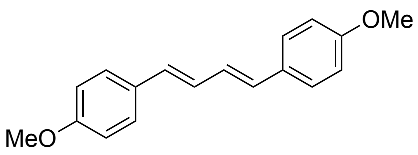

7d ^{1}H NMR ($CDCl_3$, 400 MHz)

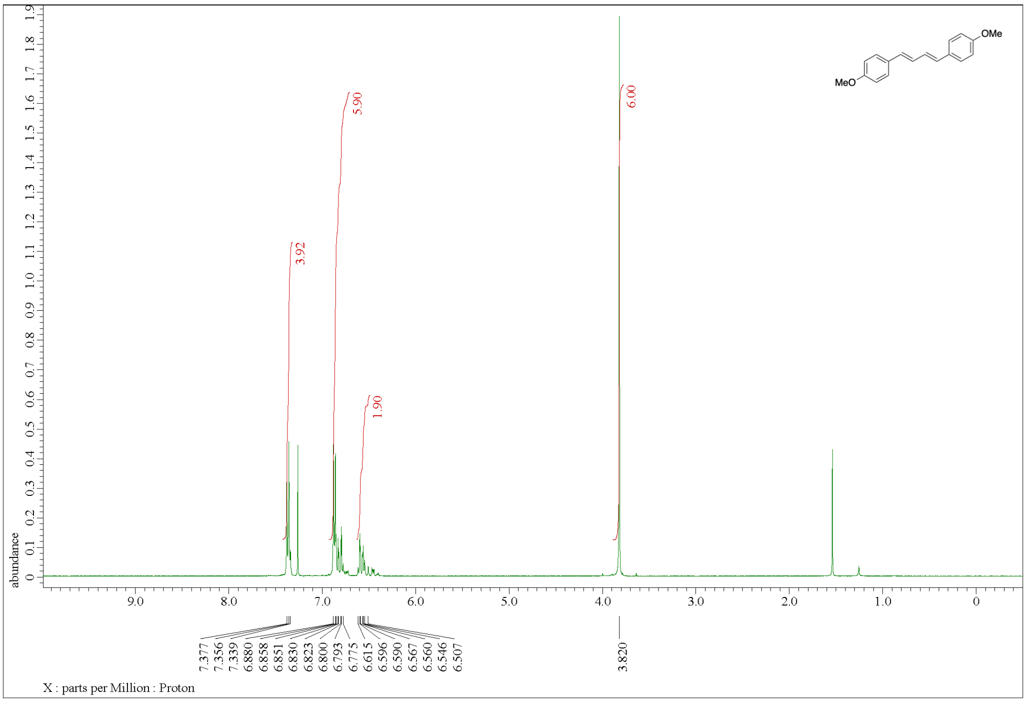

7d ^{13}C NMR ($CDCl_3$, 100 MHz)

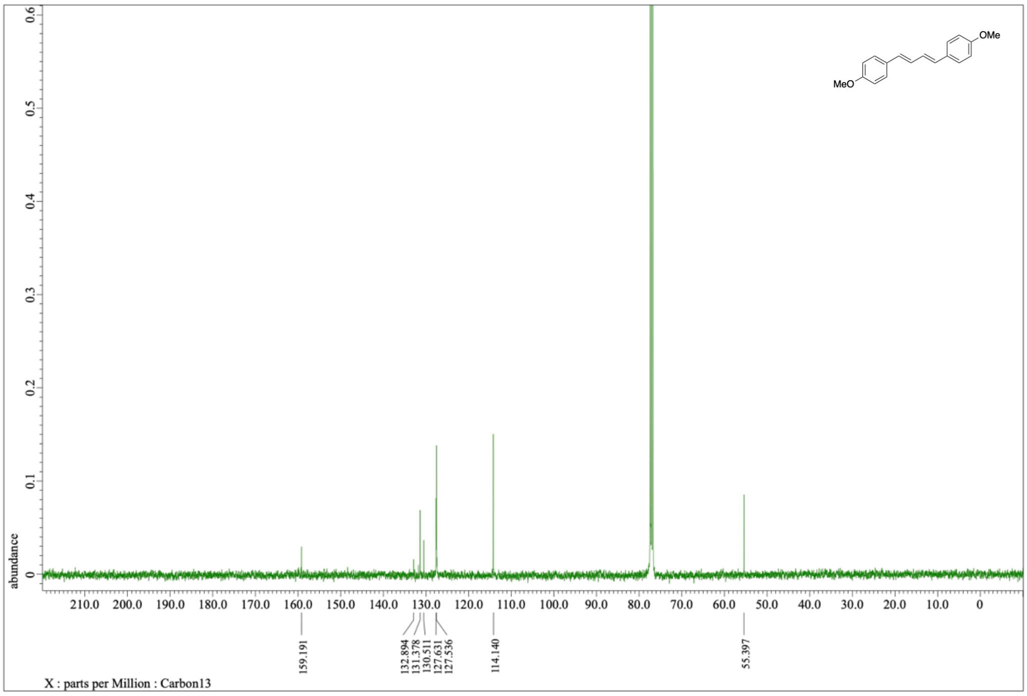

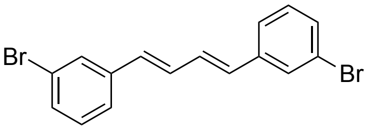

7e ^{1}H NMR ($CDCl_3$, 500 MHz)

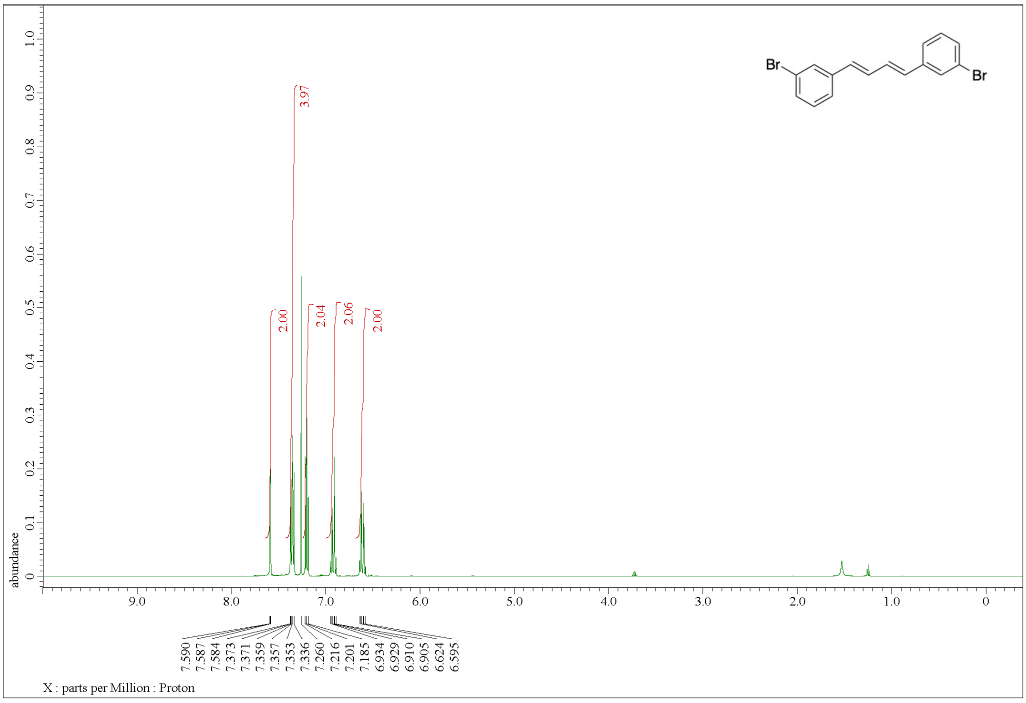

7e ^{13}C NMR ($CDCl_3$, 126 MHz)

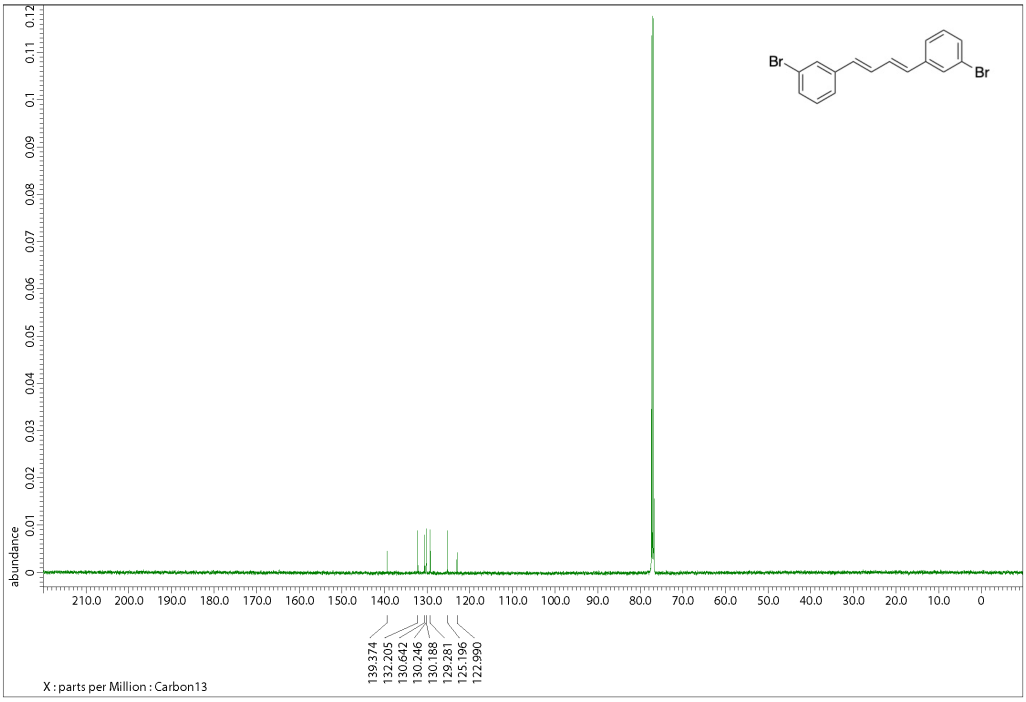

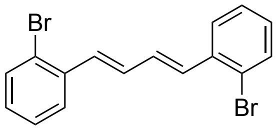

7f ^{1}H NMR ($CDCl_3$, 500 MHz)

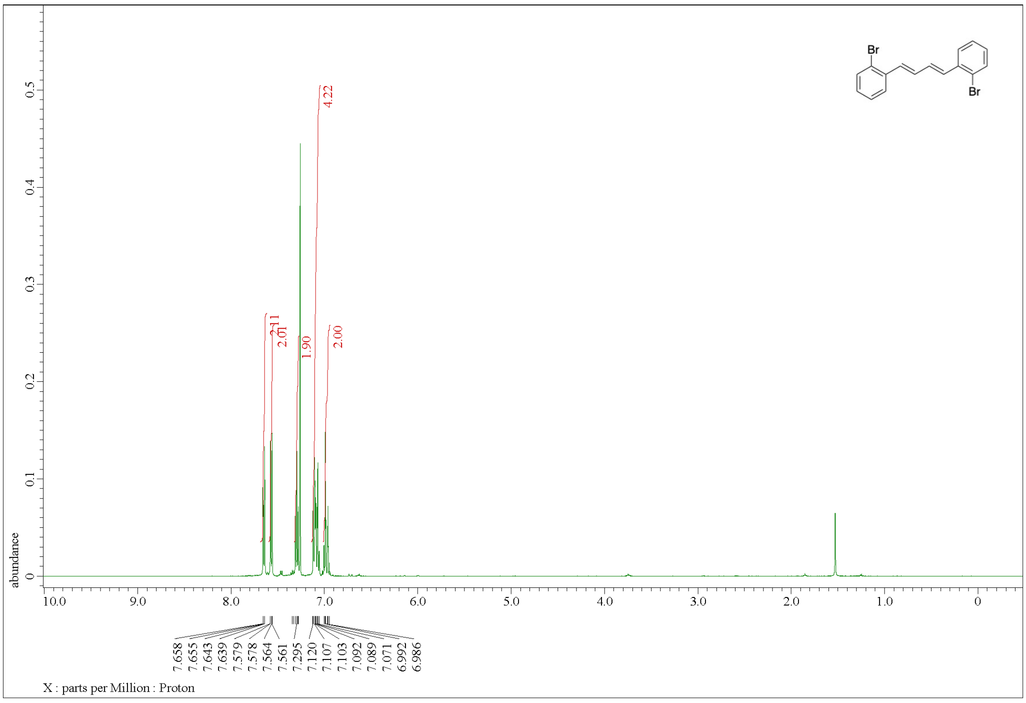

7f ^{13}C NMR ($CDCl_3$, 126 MHz)

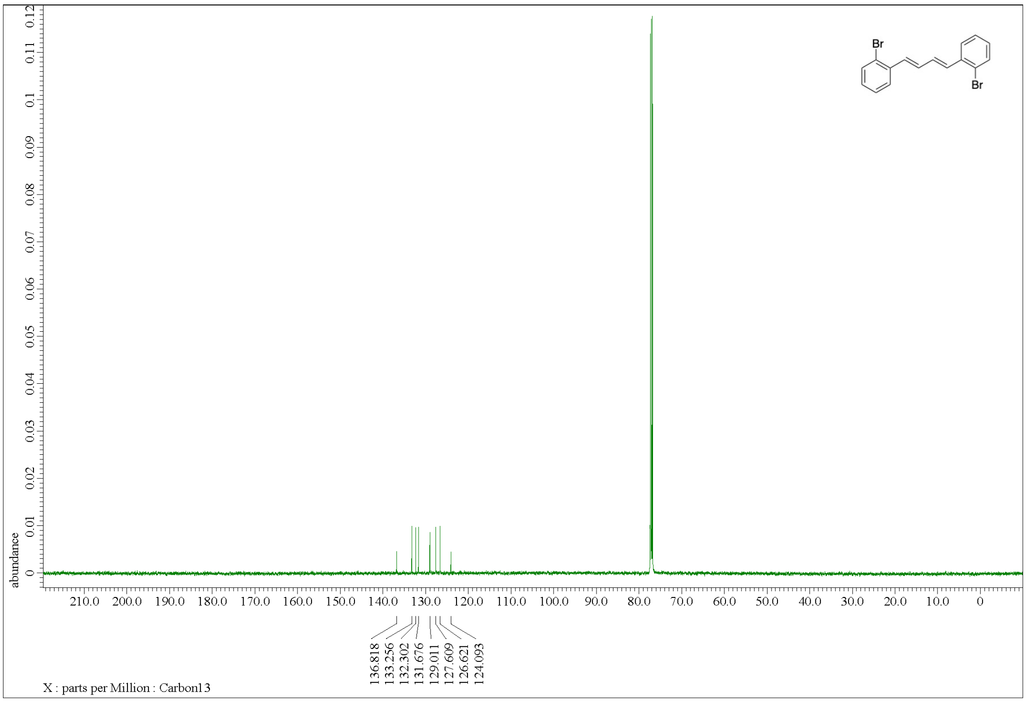

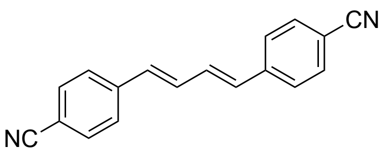

7g ^{1}H NMR ($CDCl_3$, 500 MHz)

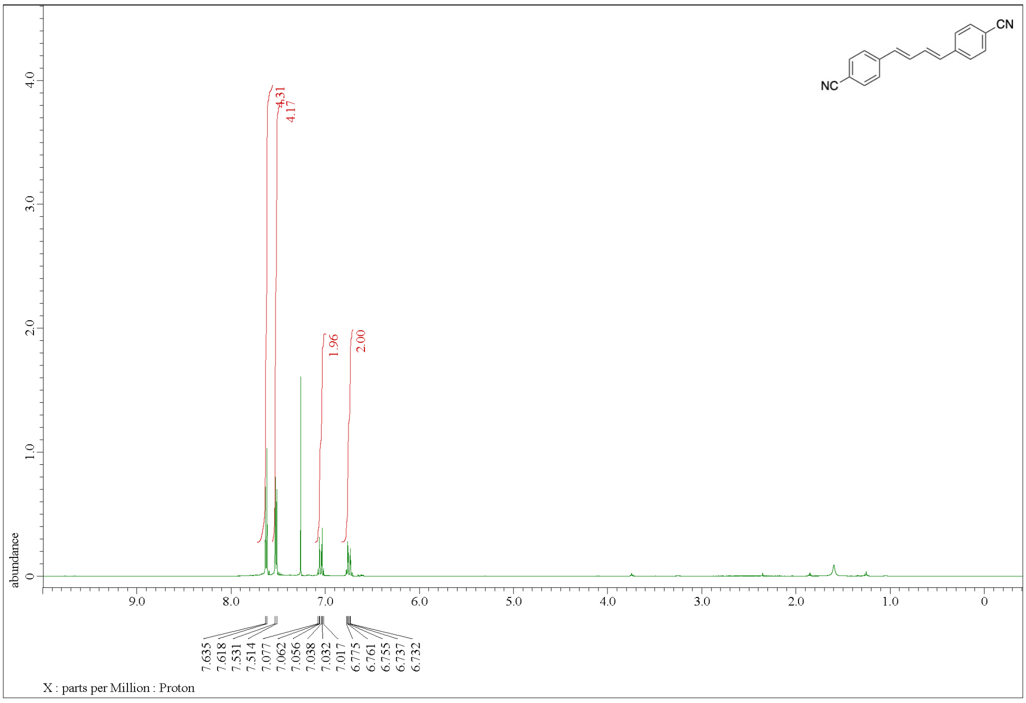

7g ^{13}C NMR ($CDCl_3$, 126 MHz)

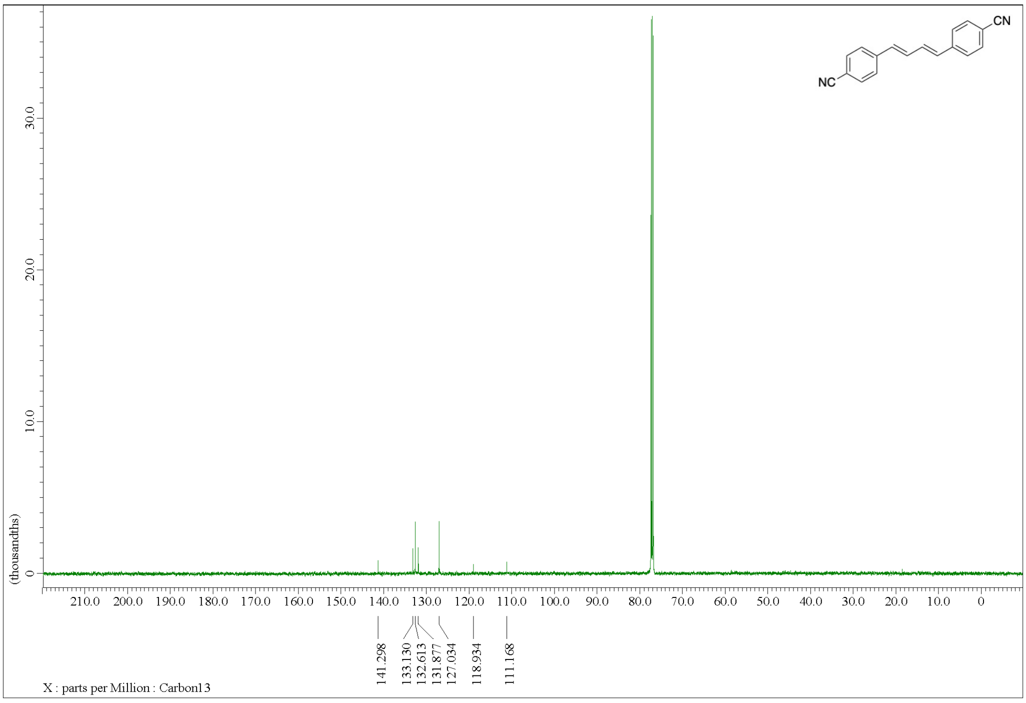

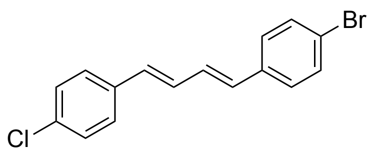

7h ^{1}H NMR ($CDCl_3$, 400 MHz)

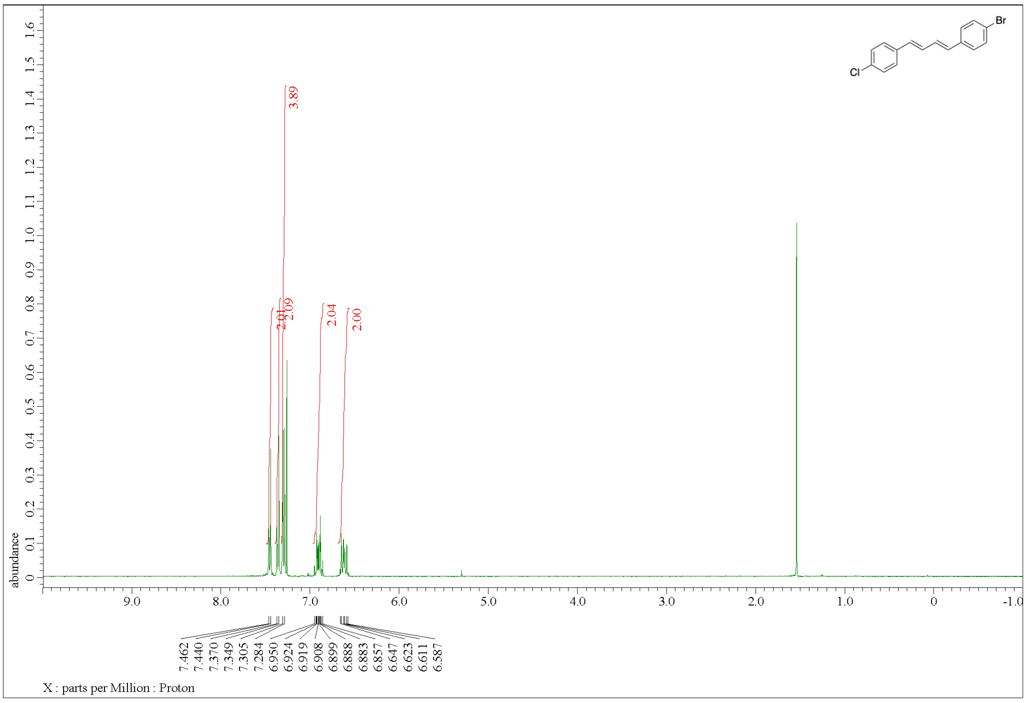

7h ^{13}C NMR ($CDCl_3$, 100 MHz)

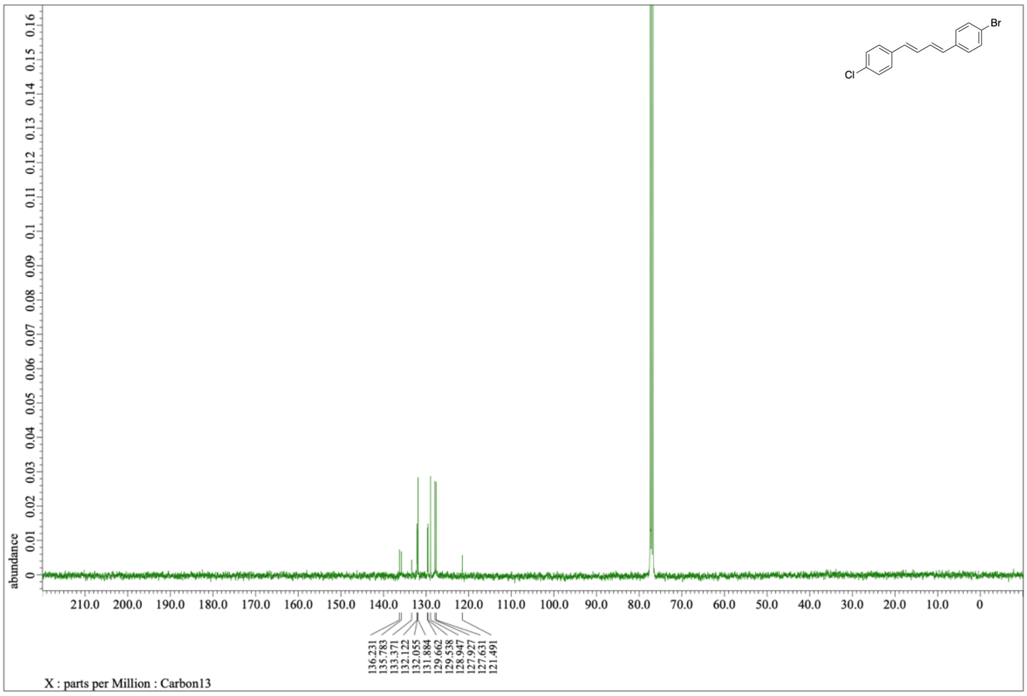

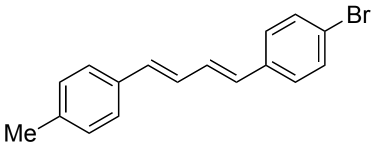

7i ^{1}H NMR ($CDCl_3$, 500 MHz)

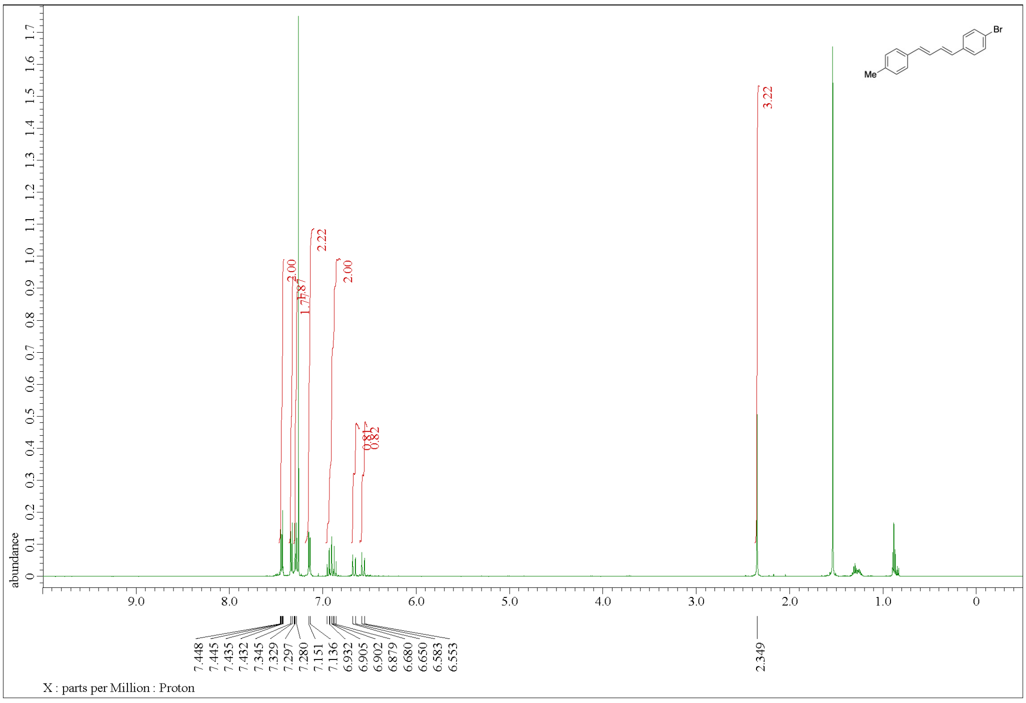

7i ^{13}C NMR ($CDCl_3$, 126 MHz)

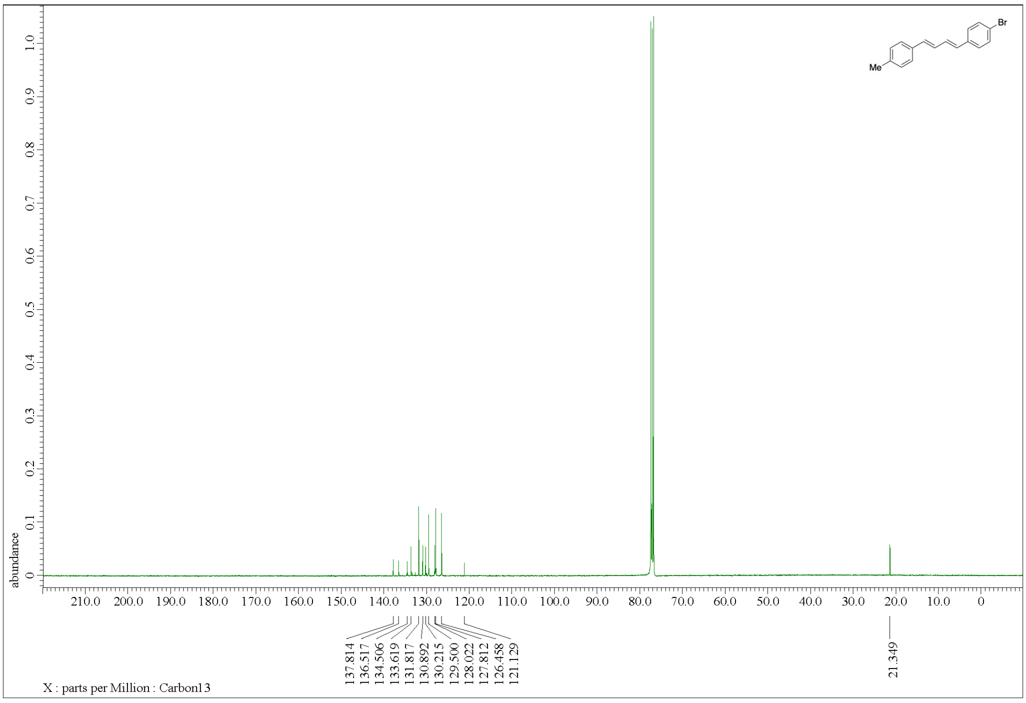

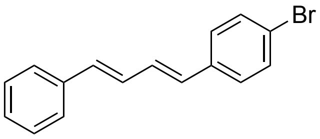

7j ^{1}H NMR ($CDCl_3$, 400 MHz)

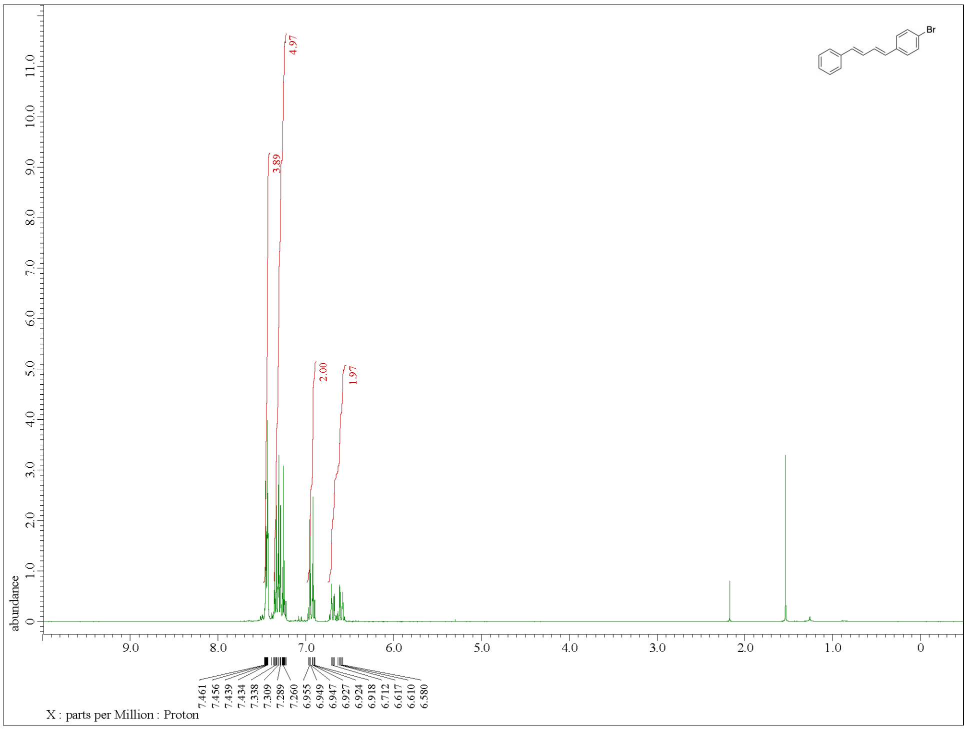

7j ^{13}C NMR ($CDCl_3$, 100 MHz)

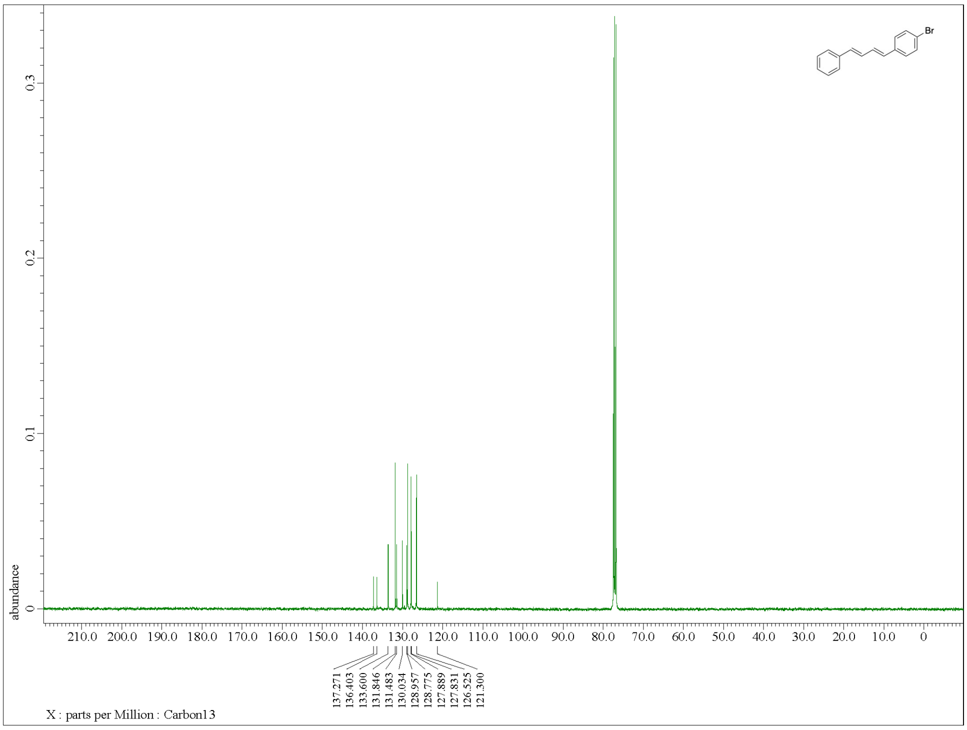

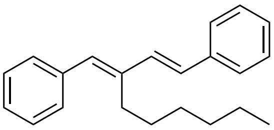

7k ^{1}H NMR ($CDCl_3$, 400 MHz)

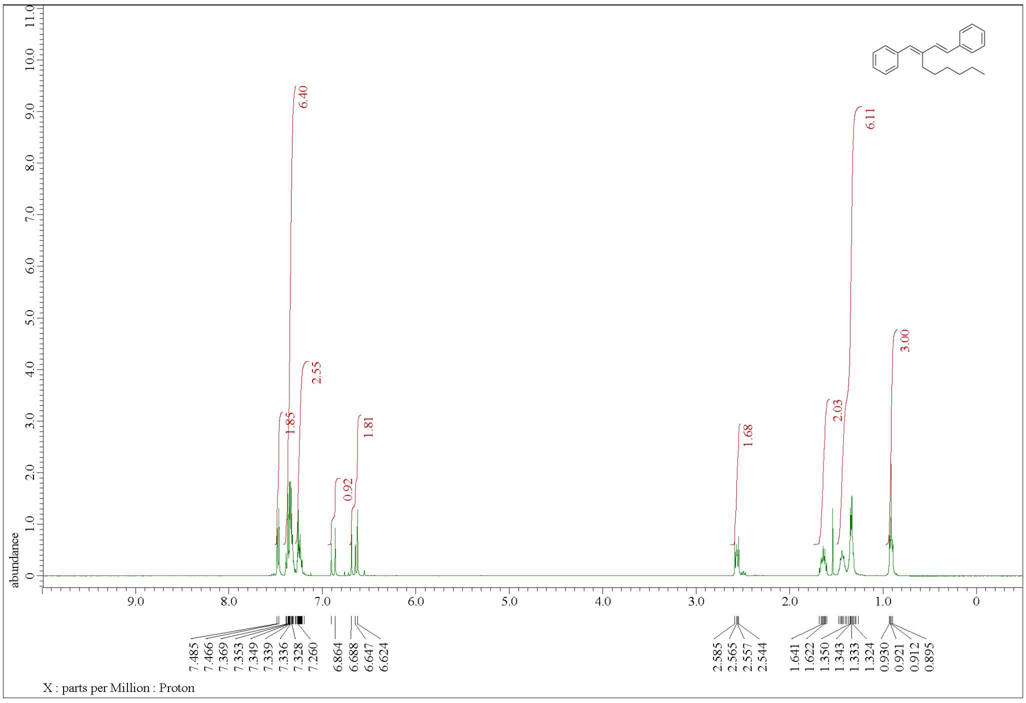

7k ^{13}C NMR ($CDCl_3$, 100 MHz)

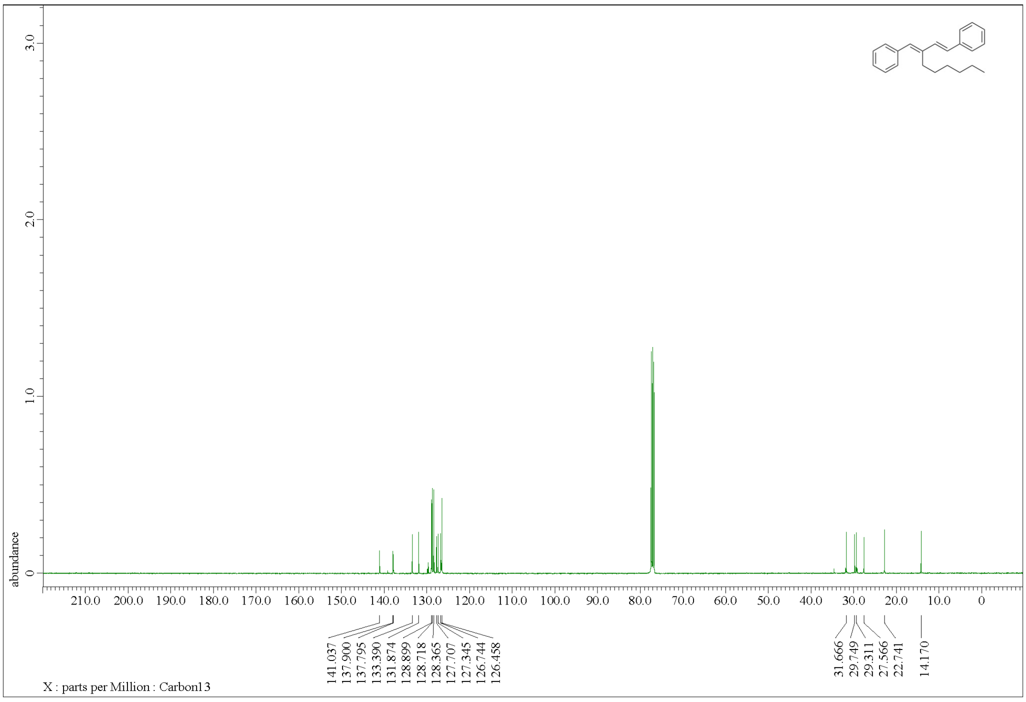

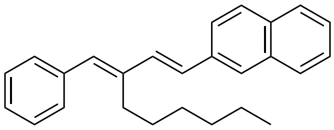

7l ^{1}H NMR ($CDCl_3$, 400 MHz)

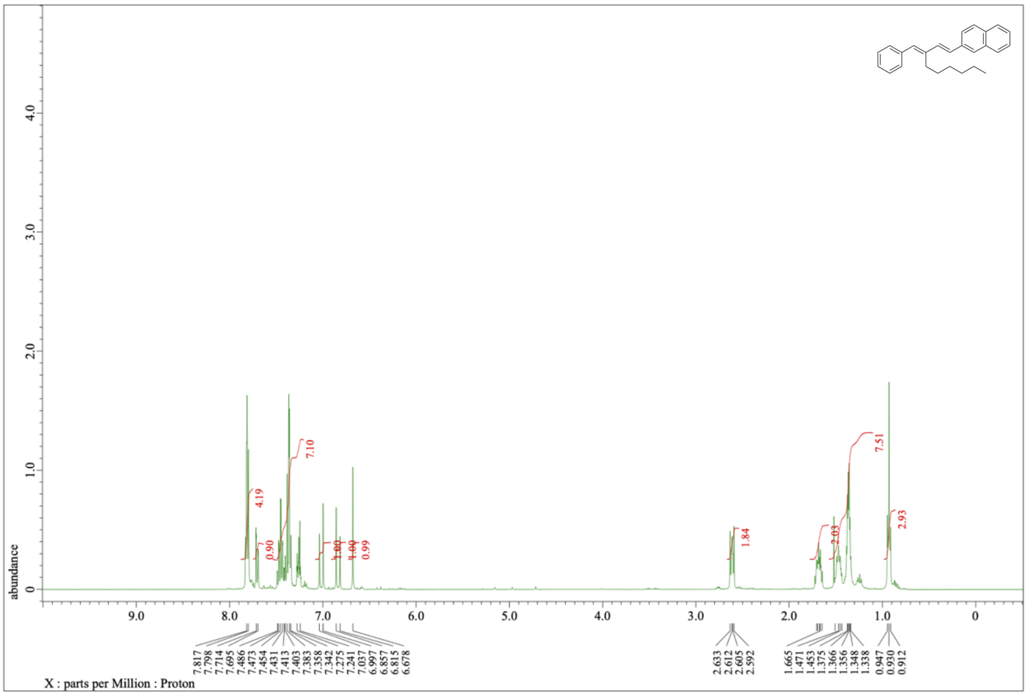

7l ^{13}C NMR ($CDCl_3$, 100 MHz)

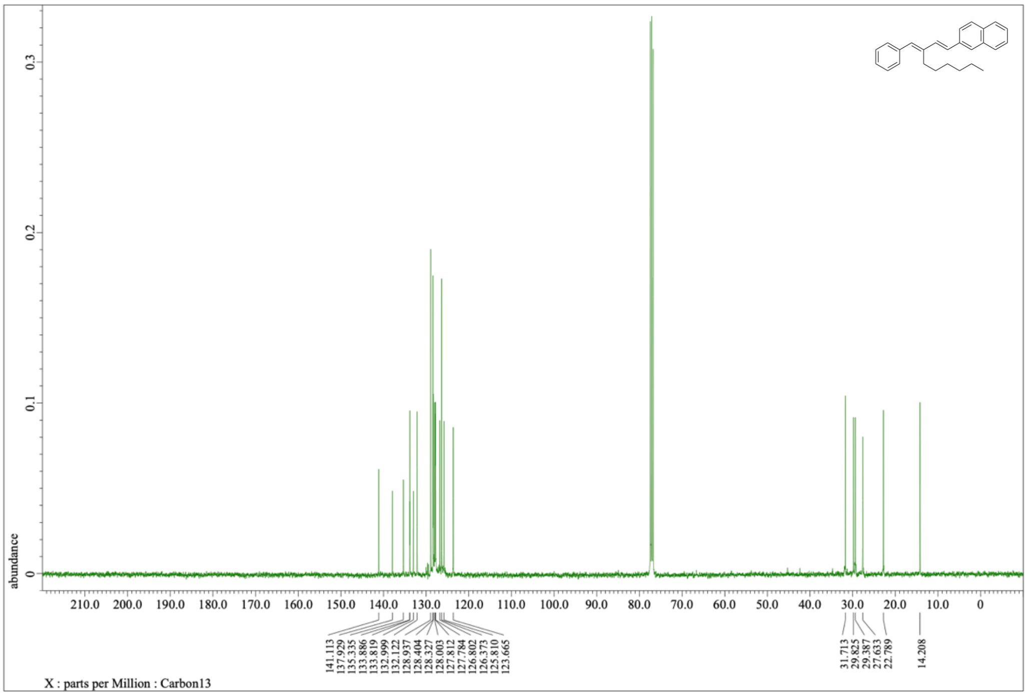

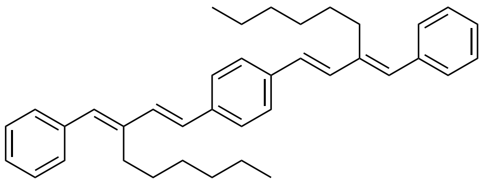

7m ^1H NMR ($CDCl_3$, 400 MHz)

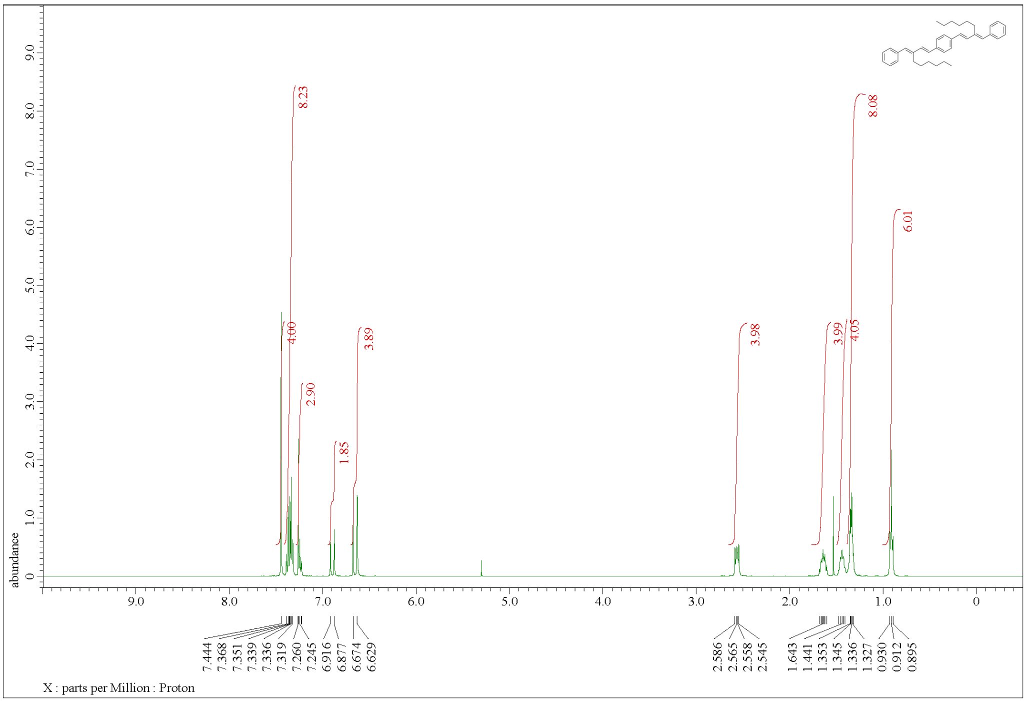

7m ^{13}C NMR ($CDCl_3$, 100 MHz)

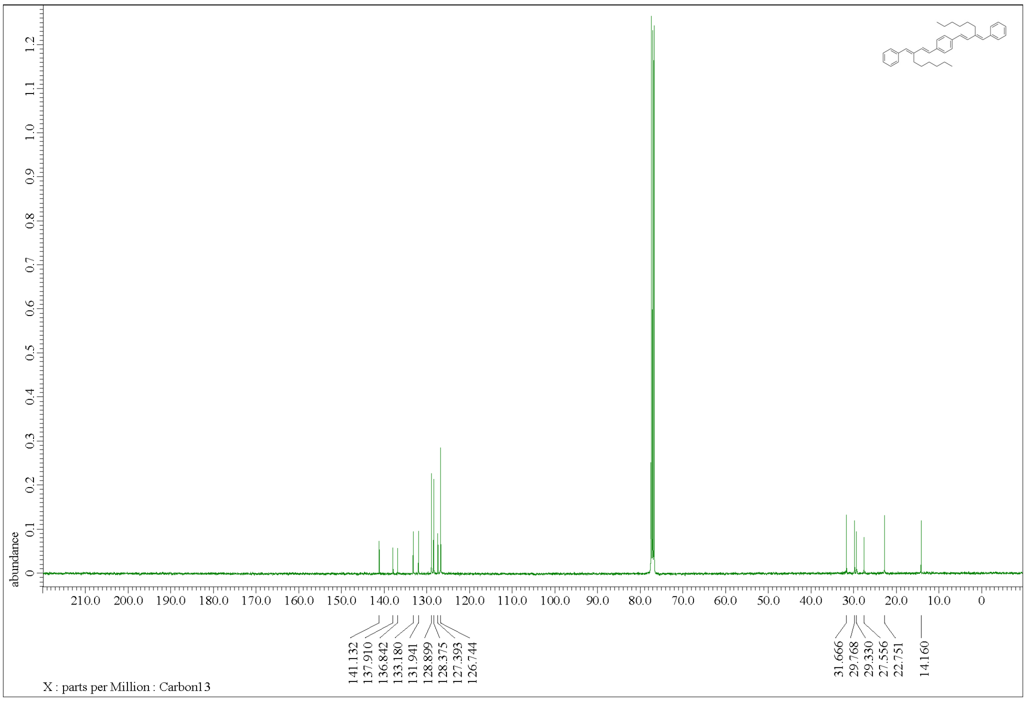

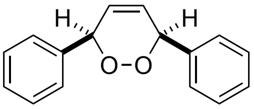

3 ^{1}H NMR ($CDCl_3$, 500 MHz)

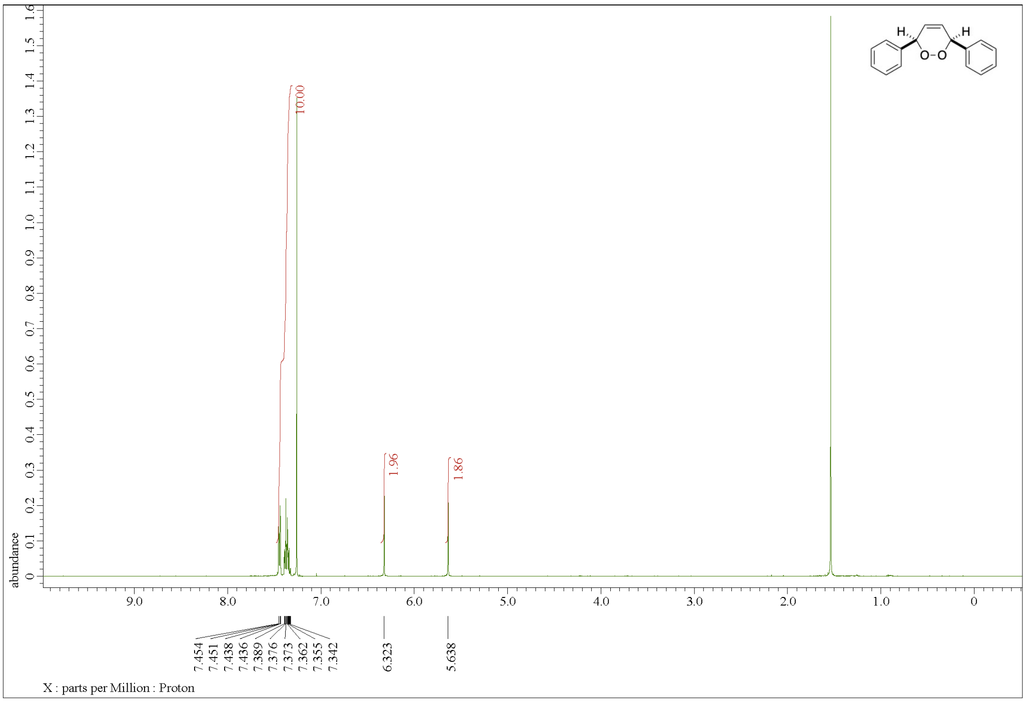

3 ^{13}C NMR ($CDCl_3$, 126 MHz)

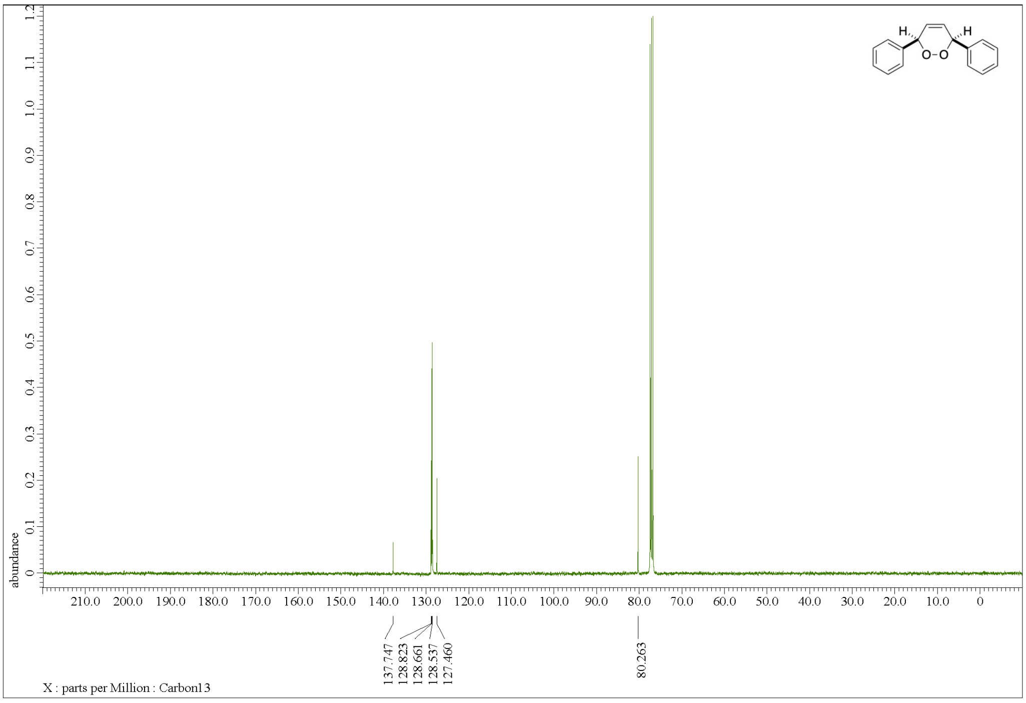

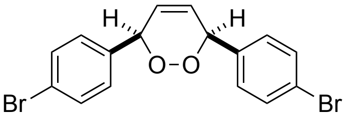

8a ^{1}H NMR ($CDCl_3$, 500 MHz)

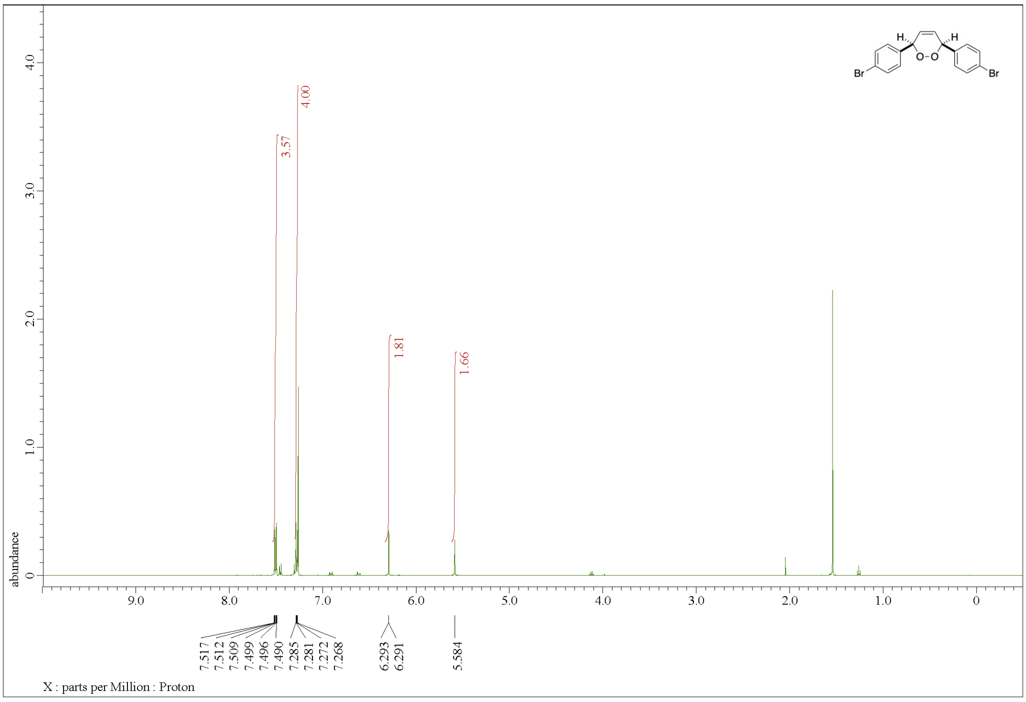

8a ^{13}C NMR ($CDCl_3$, 126 MHz)

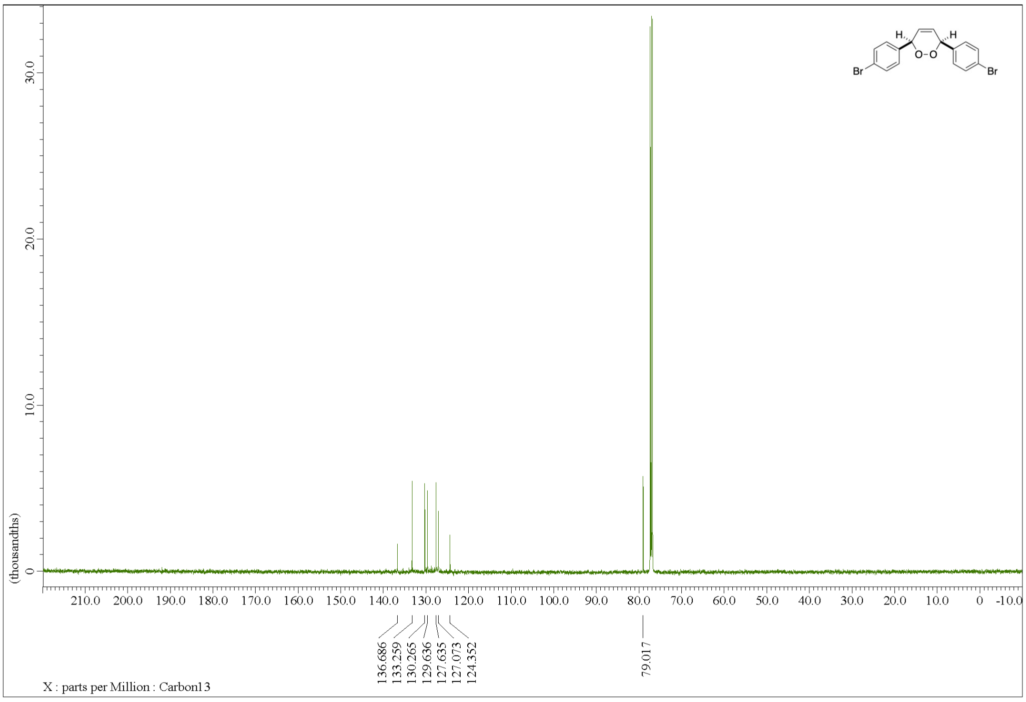

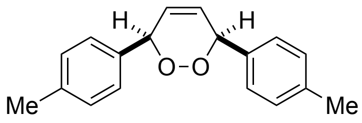

8b ^{1}H NMR ($CDCl_3$, 500 MHz)

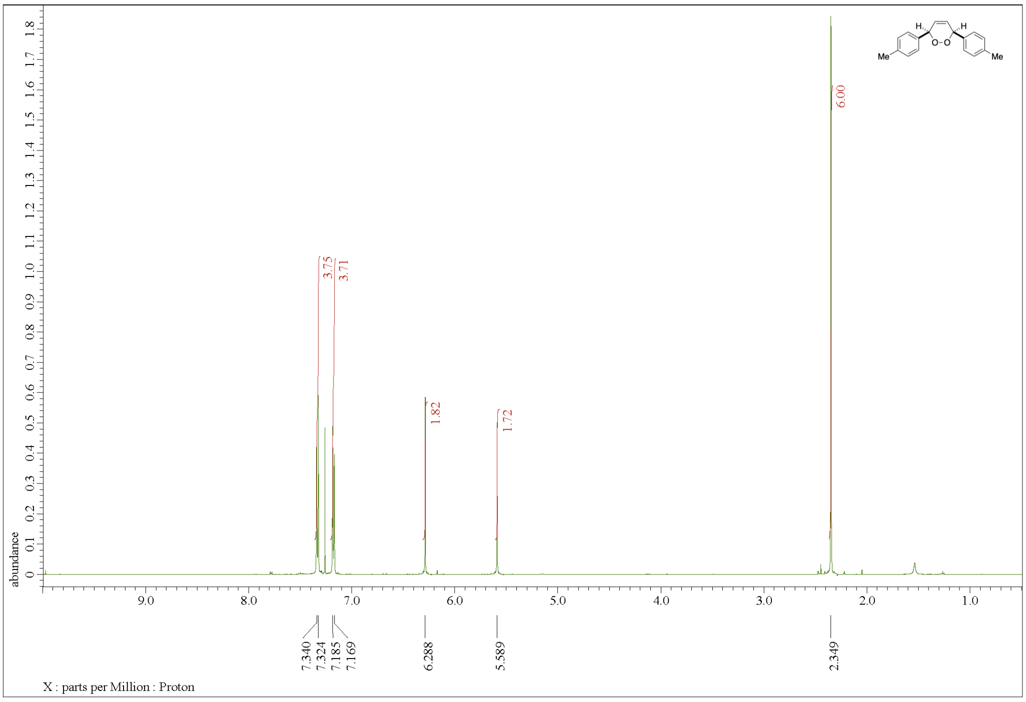

8b ^{13}C NMR ($CDCl_3$, 126 MHz)

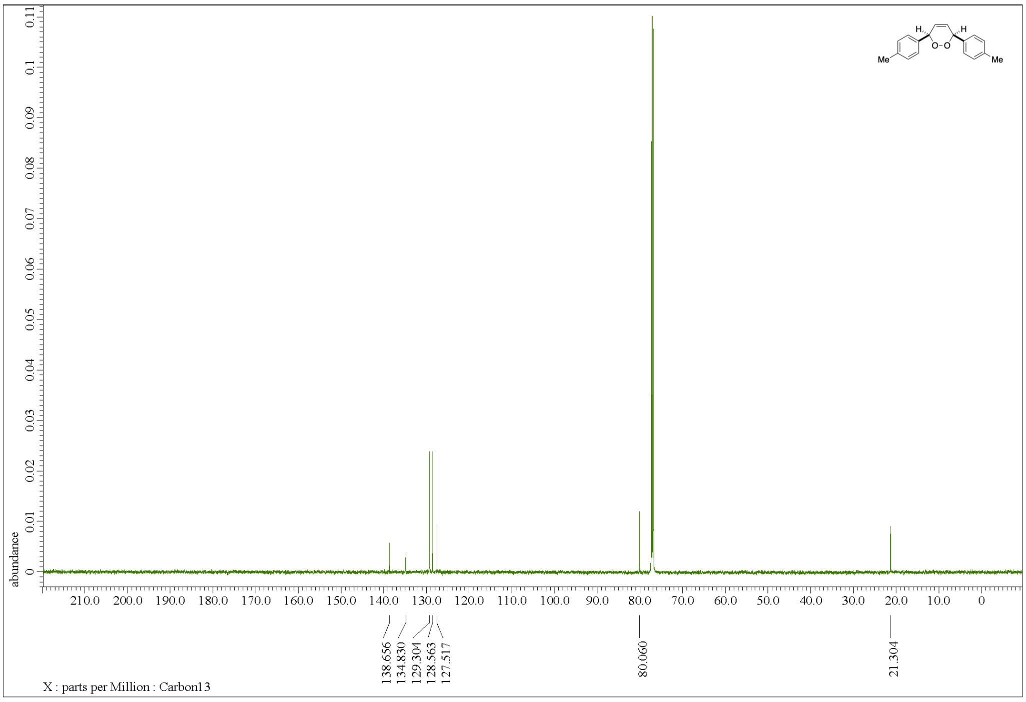

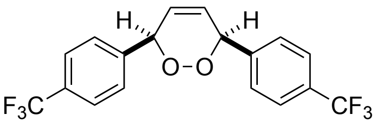

8c ^{1}H NMR ($CDCl_3$, 500 MHz)

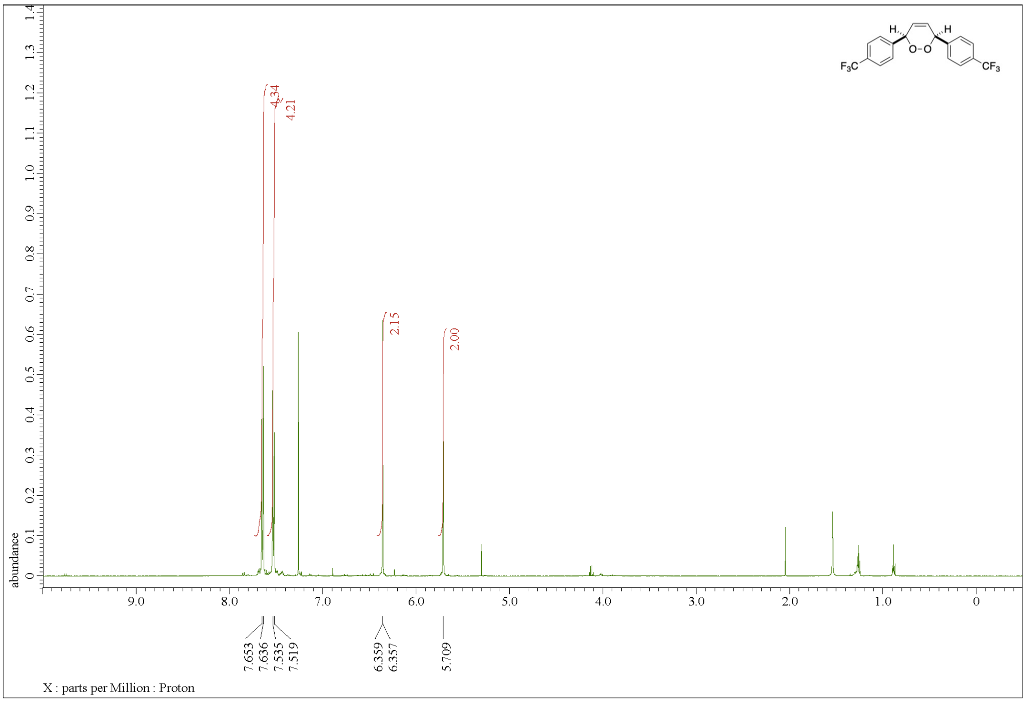

8c ^{13}C NMR ($CDCl_3$, 126 MHz)

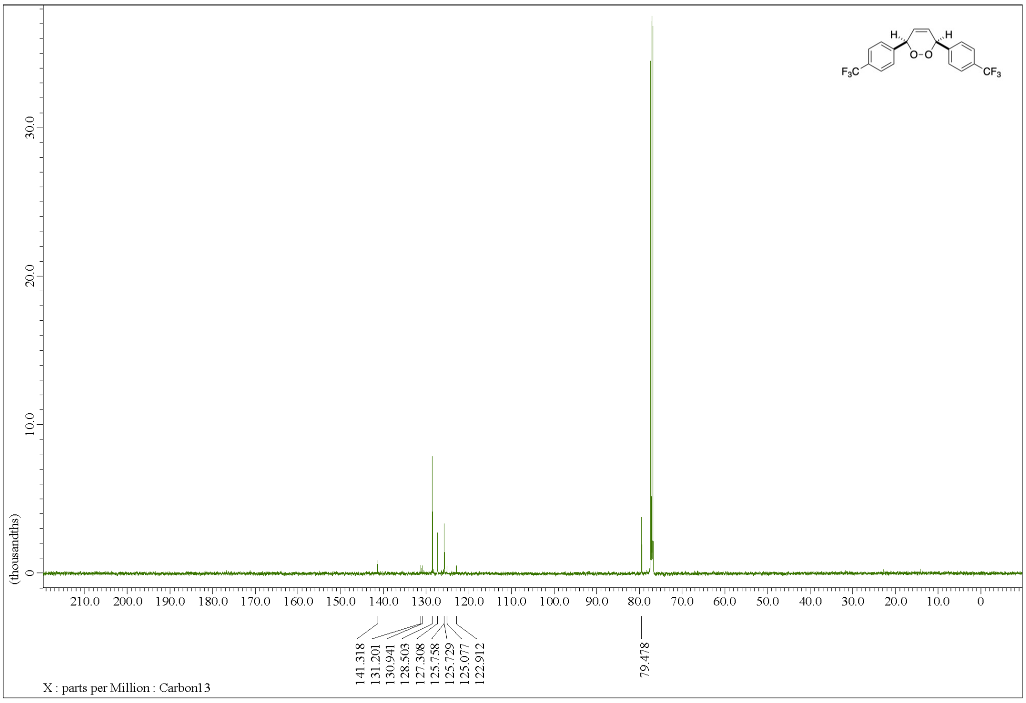

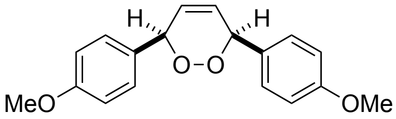

8d ^{1}H NMR ($CDCl_3$, 500 MHz)

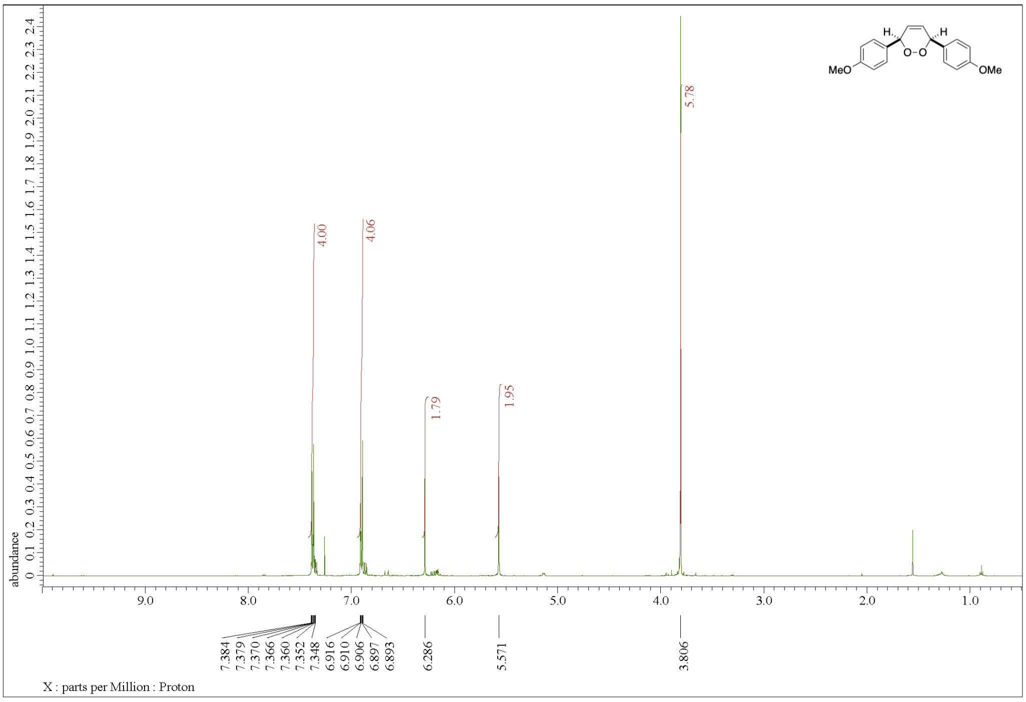

8d ^{13}C NMR ($CDCl_3$, 126 MHz)

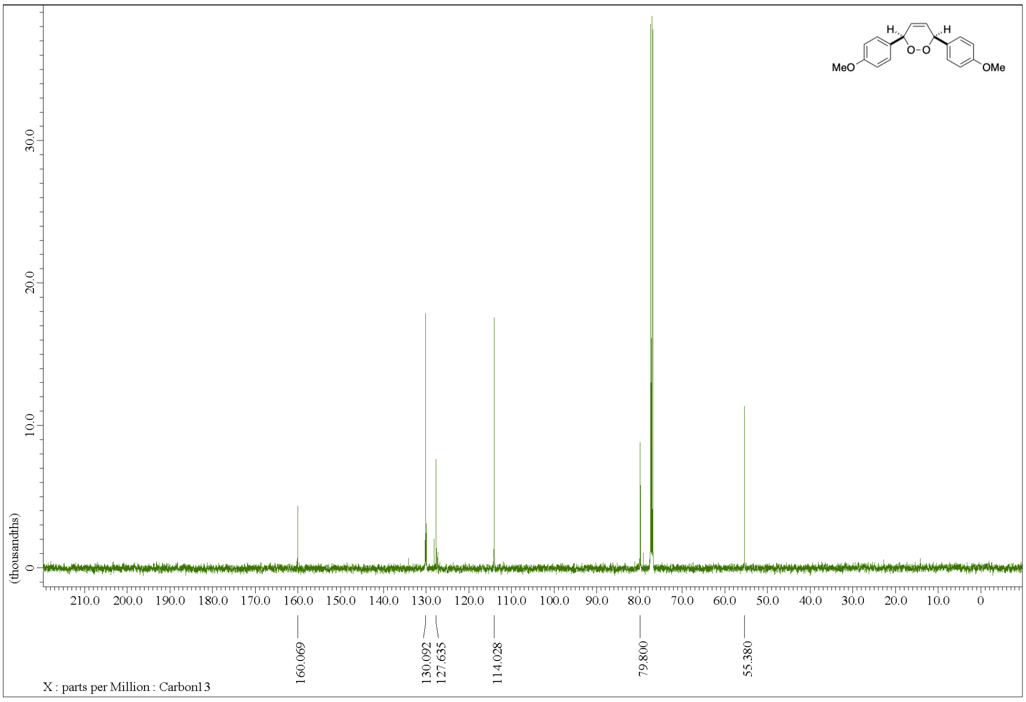

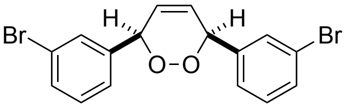

8e ^{1}H NMR ($CDCl_3$, 500 MHz)

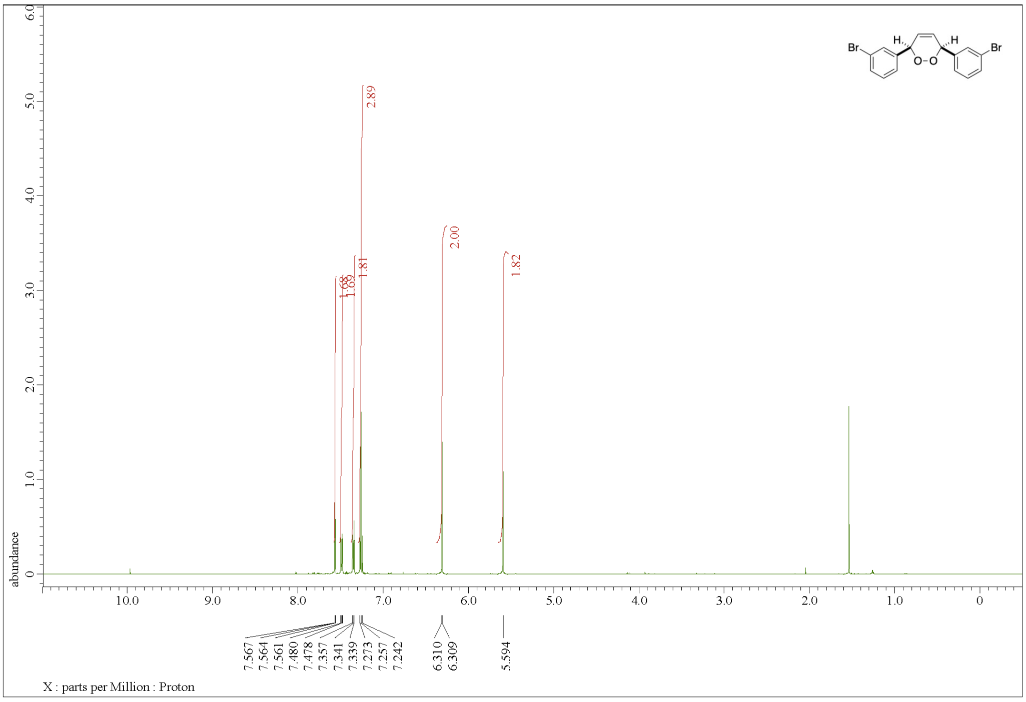

8e ^{13}C NMR ($CDCl_3$, 126 MHz)

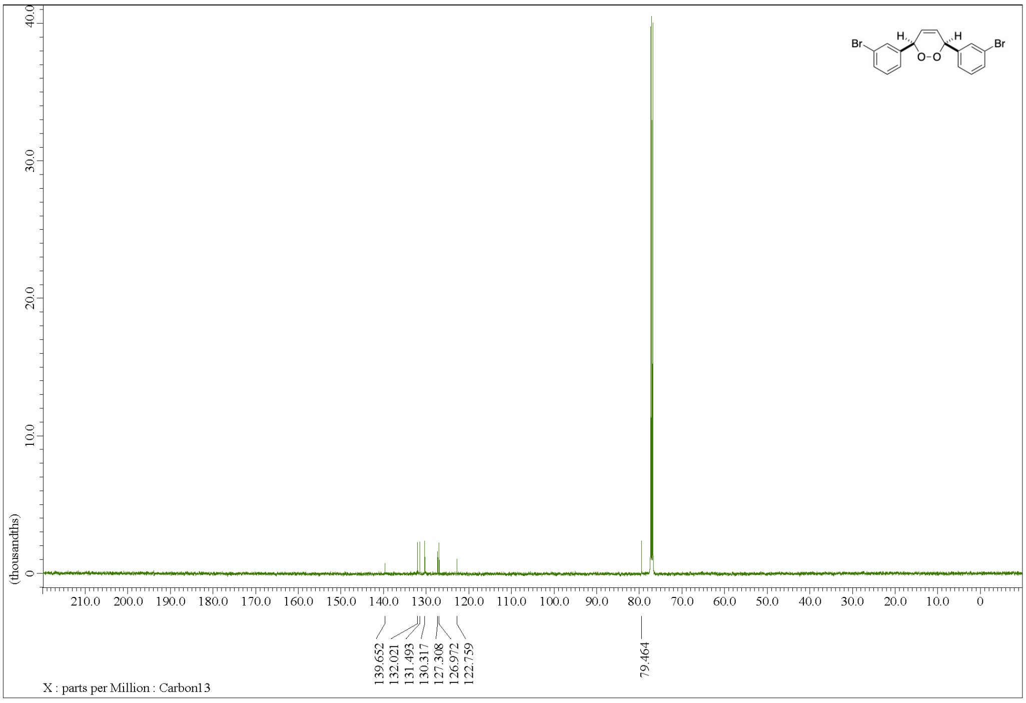

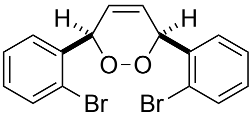

8f ^{1}H NMR ($CDCl_3$, 500 MHz)

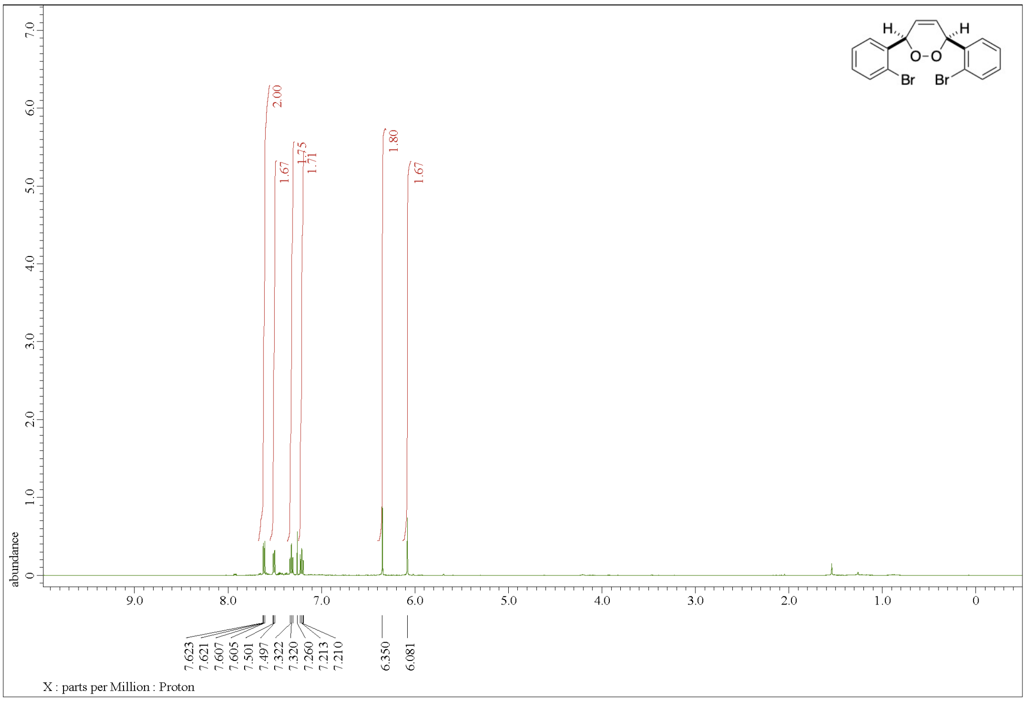

8f ^{13}C NMR ($CDCl_3$, 126 MHz)

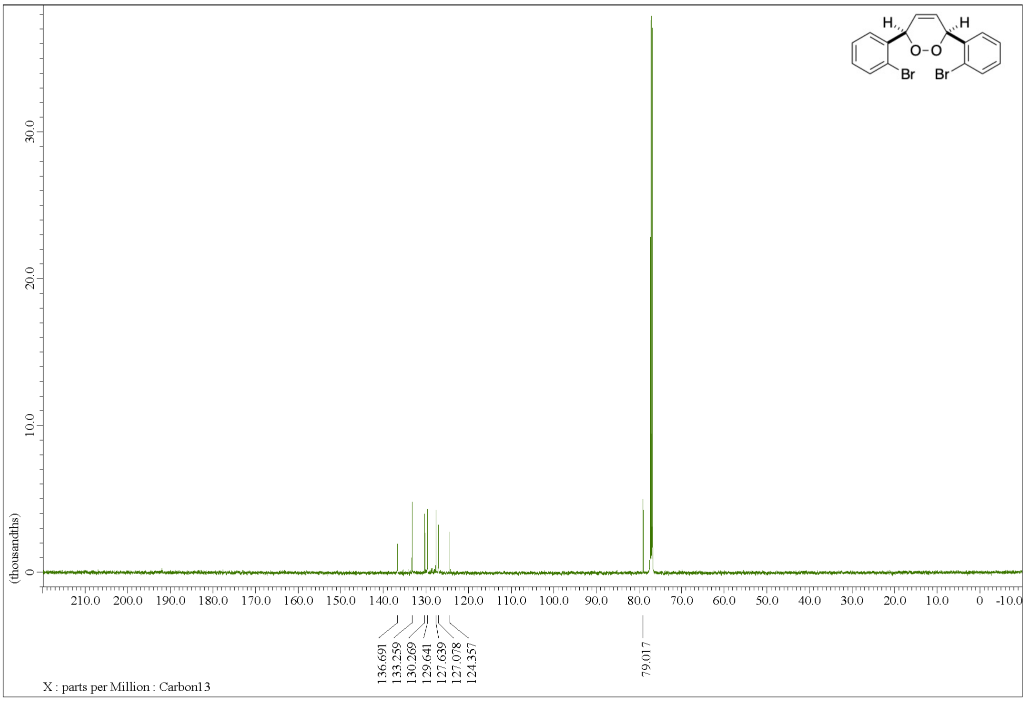

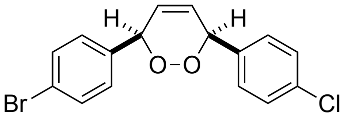

8h ^{1}H NMR ($CDCl_3$, 400 MHz)

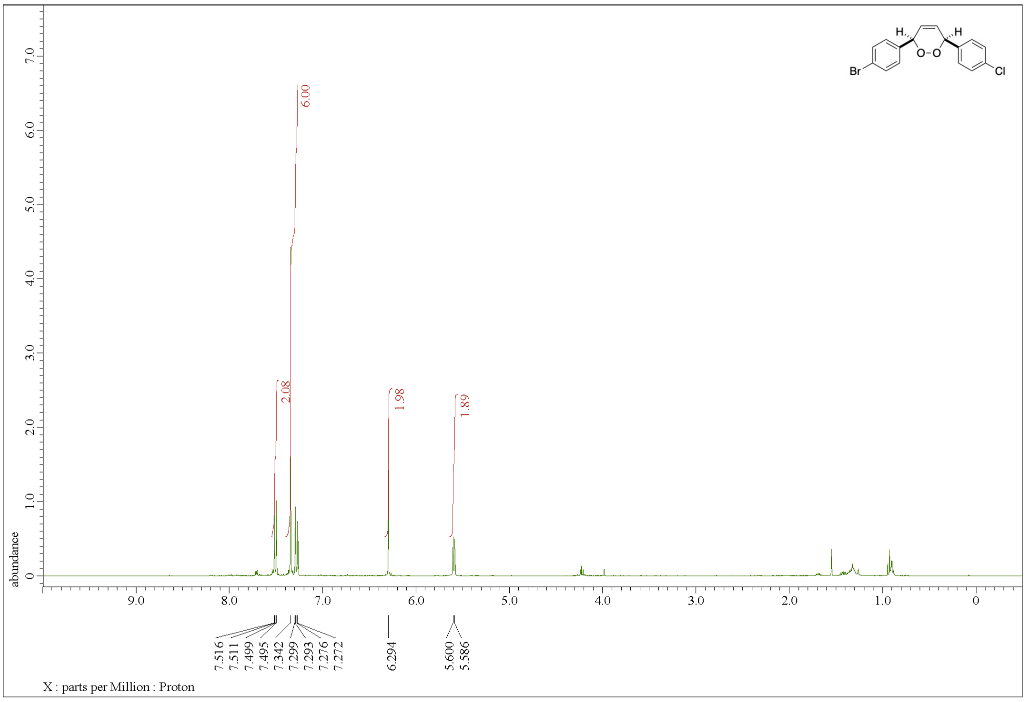

8h ^{13}C NMR ($CDCl_3$, 100 MHz)

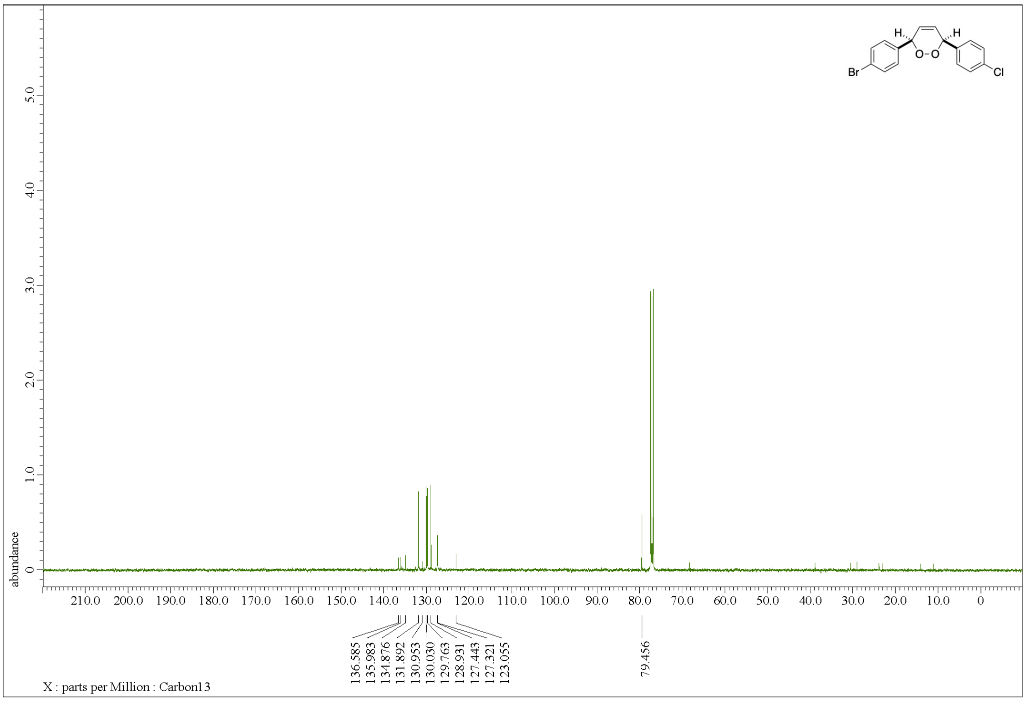

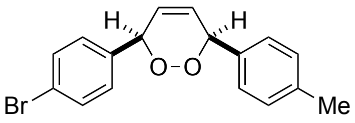

8i ^{1}H NMR ($CDCl_3$, 400 MHz)

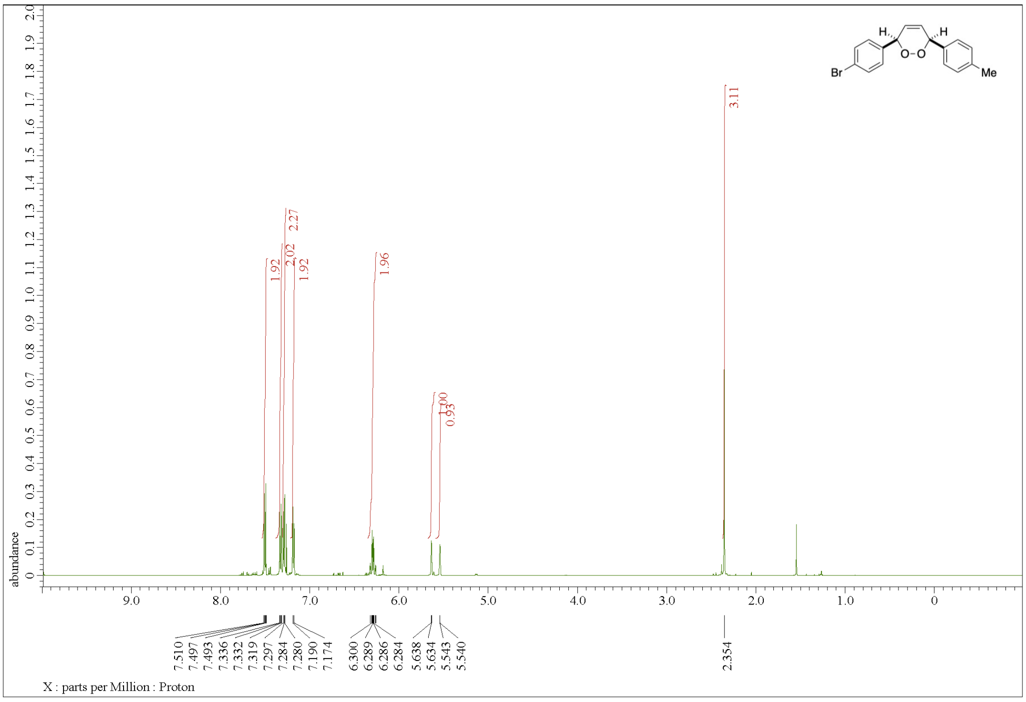

8i ^{13}C NMR ($CDCl_3$, 100 MHz)

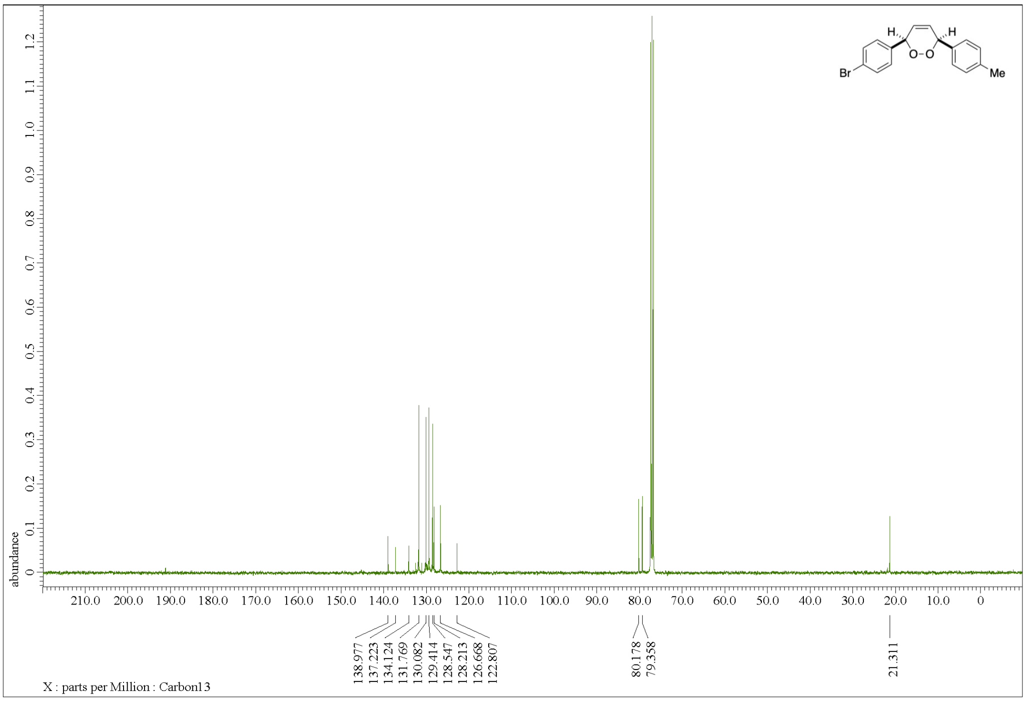

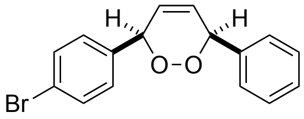

8j ^{1}H NMR ($CDCl_3$, 500 MHz)

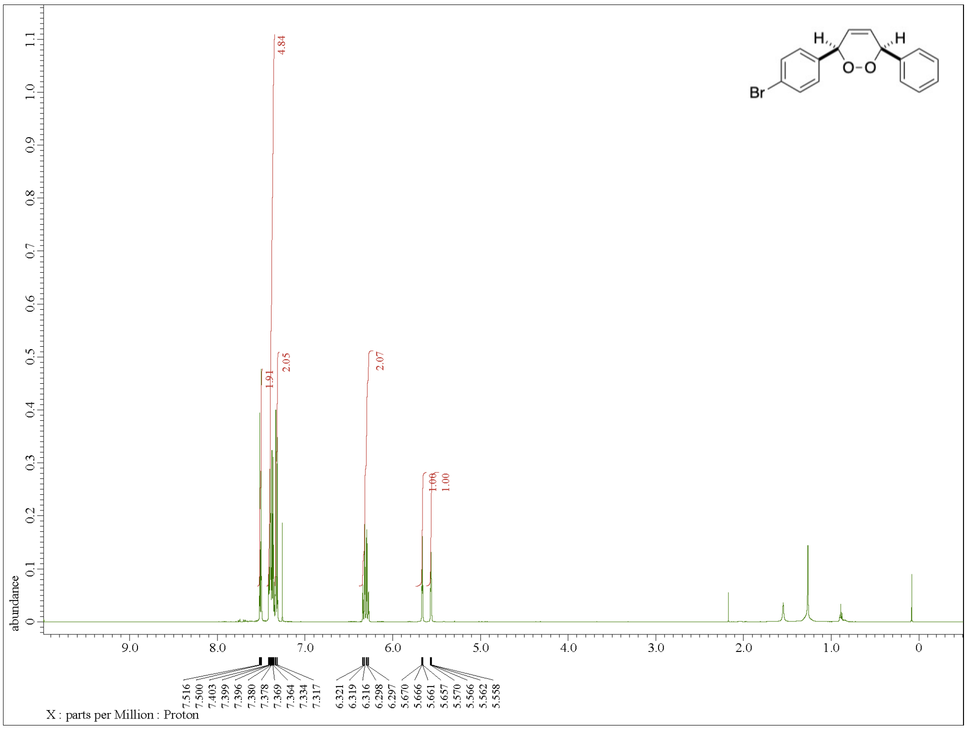

8j ^{13}C NMR ($CDCl_3$, 126 MHz)

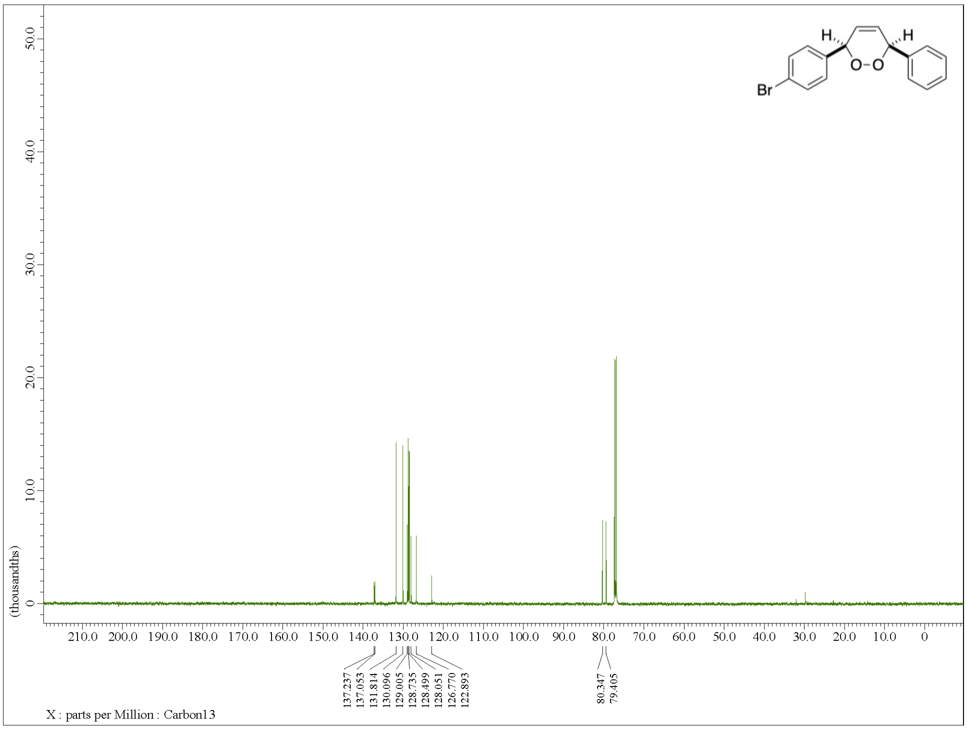

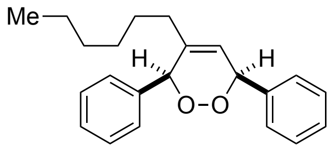

8k ^{1}H NMR ($CDCl_3$, 500 MHz)

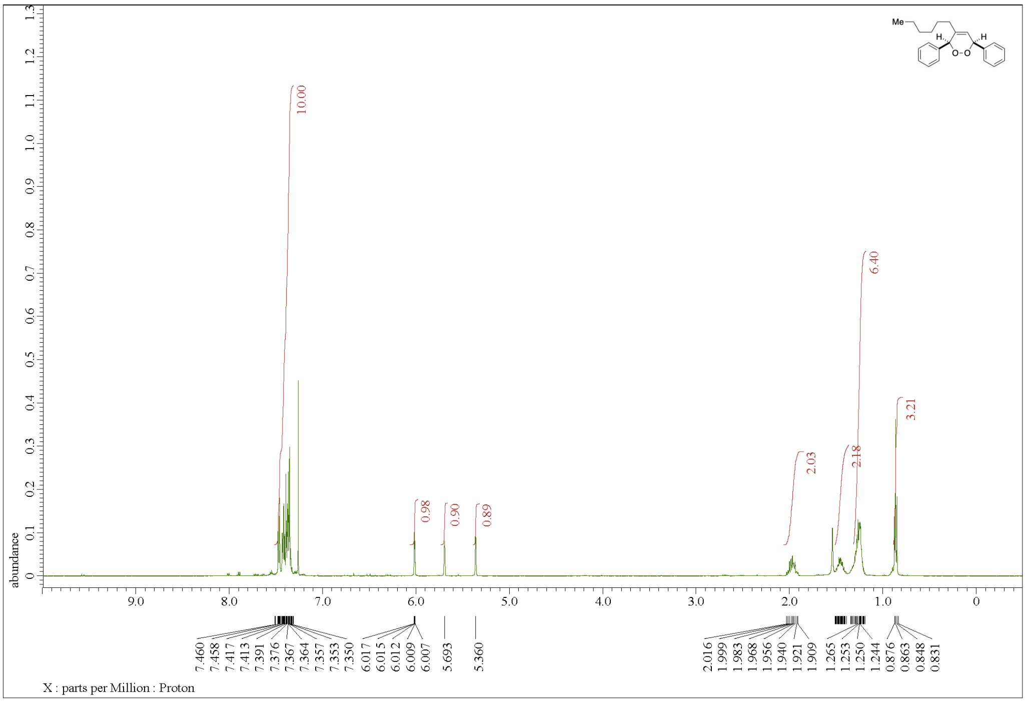

8k ^{13}C NMR ($CDCl_3$, 126 MHz)

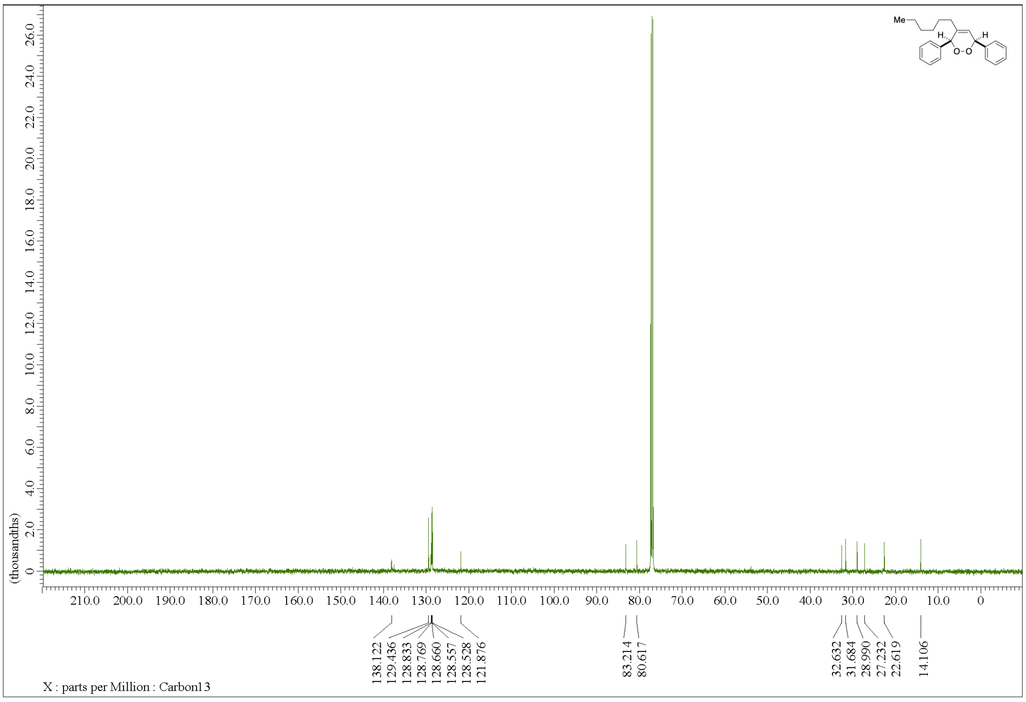

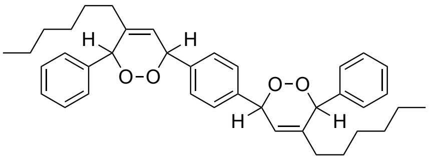

8m ^{1}H NMR ($CDCl_3$, 500 MHz)

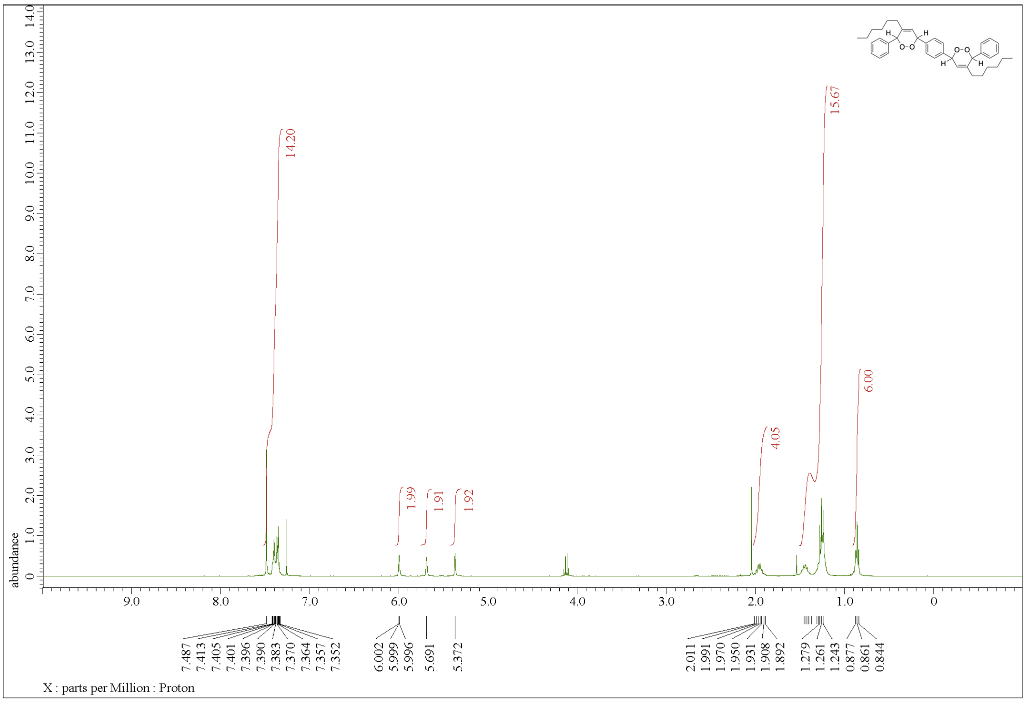

8m ^{13}C NMR ($CDCl_3$, 126 MHz)

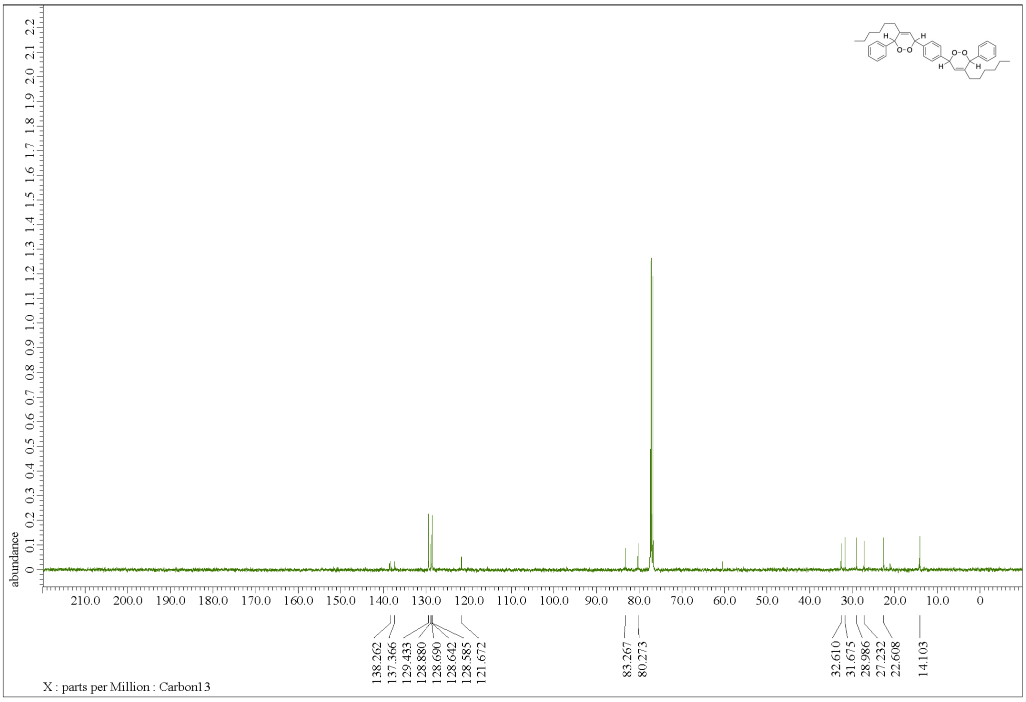

NMR spectra of prepared compounds – furans

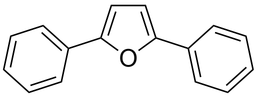

2 ^{1}H NMR ($CDCl_3$, 500 MHz)

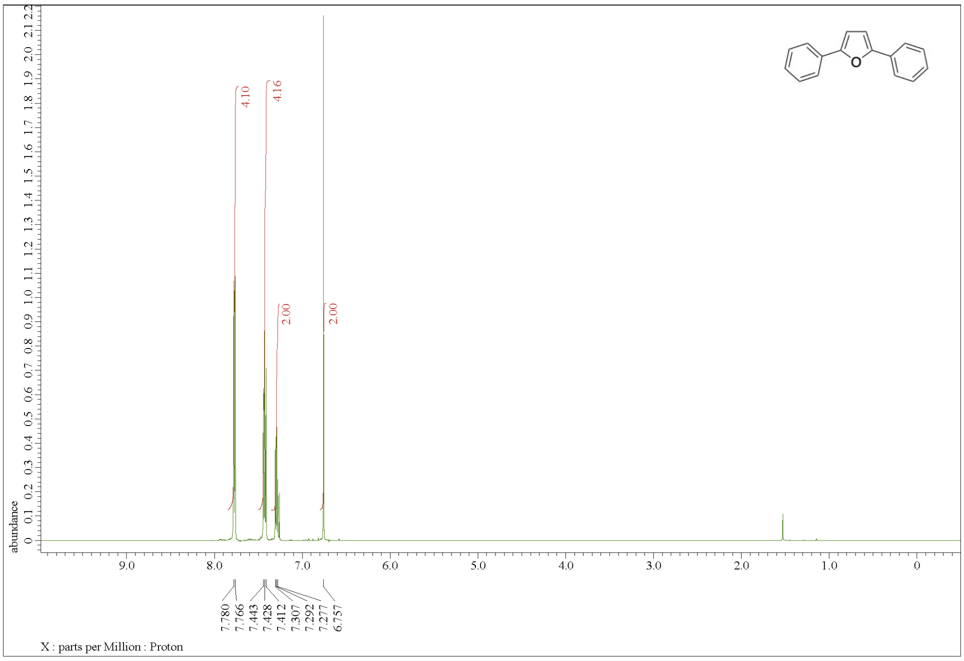

2 ^{13}C NMR ($CDCl_3$, 126 MHz)

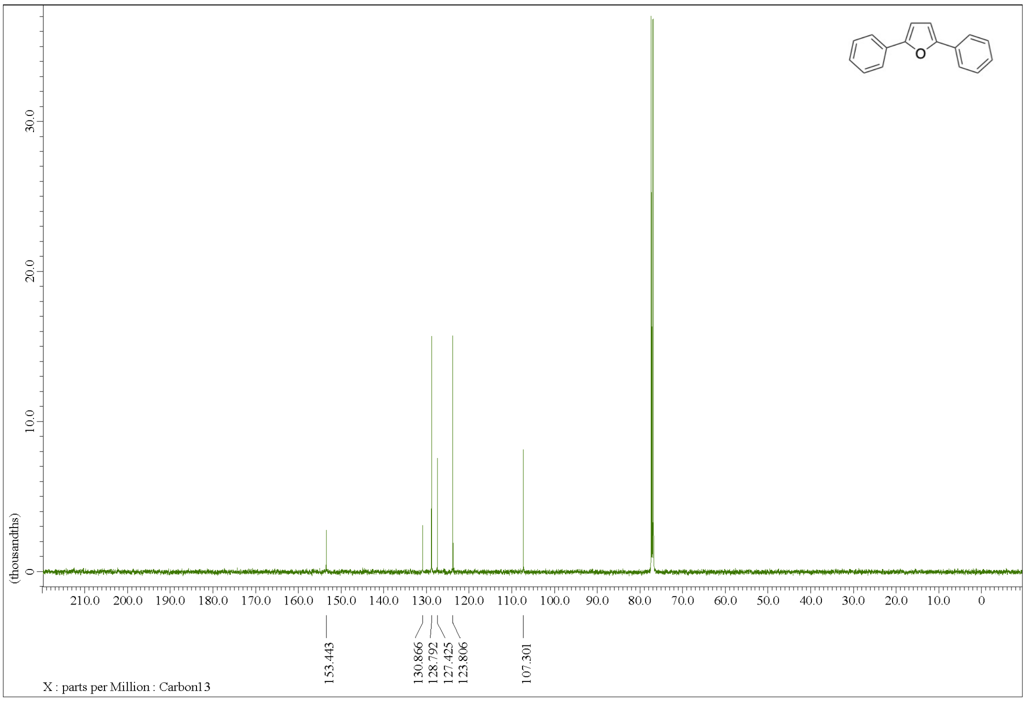

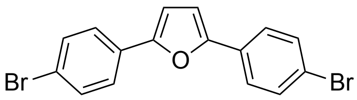

9a ^{1}H NMR ($CDCl_3$, 400 MHz)

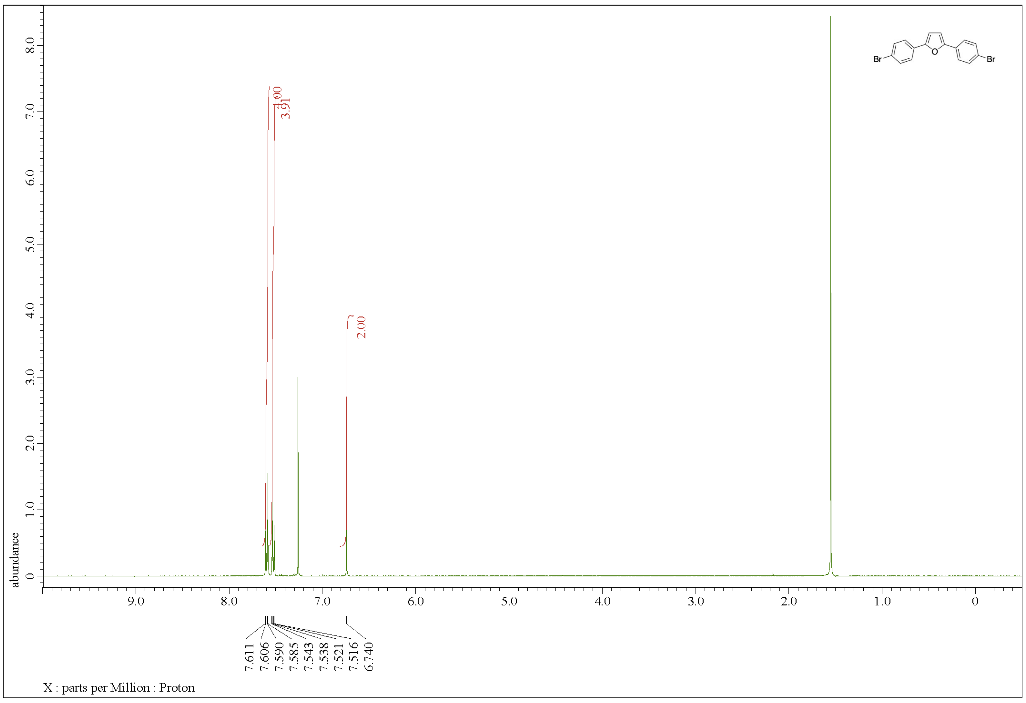

9a ^{13}C NMR ($CDCl_3$, 100 MHz)

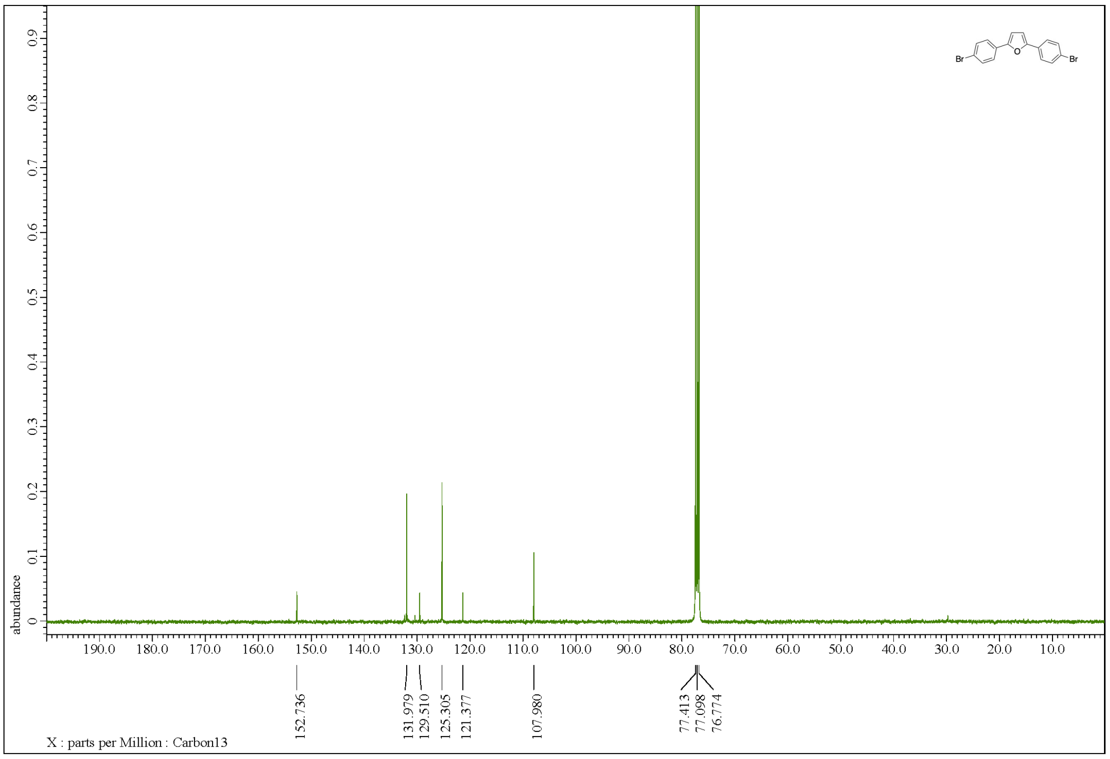

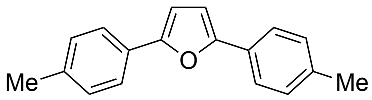

9b ^{1}H NMR ($CDCl_3$, 500 MHz)

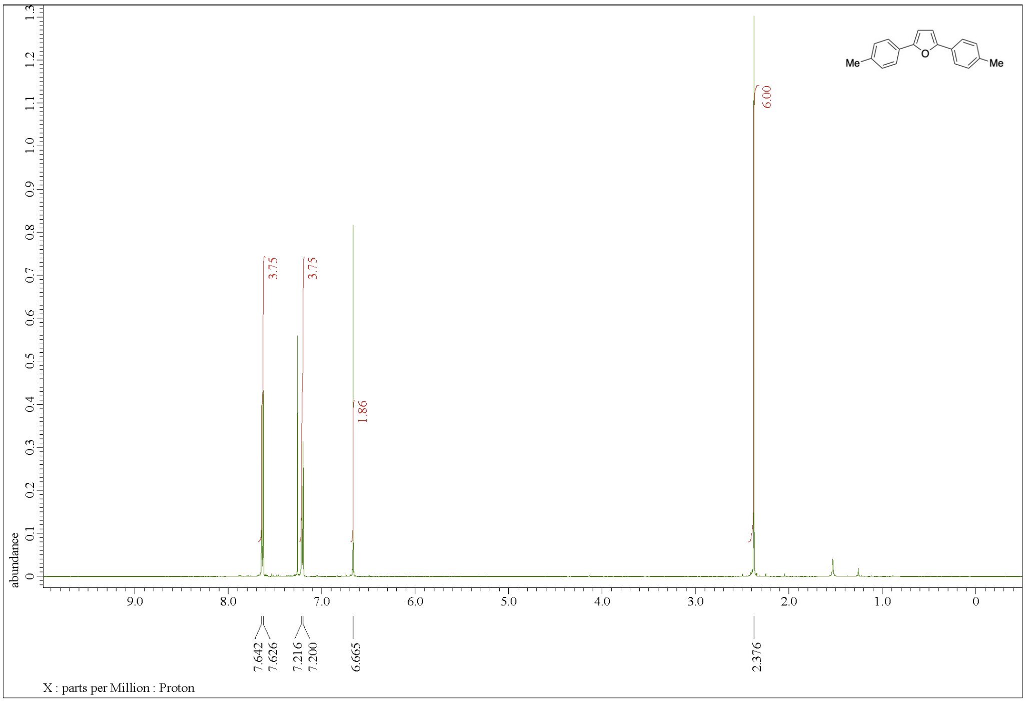

9b ^{13}C NMR ($CDCl_3$, 126 MHz)

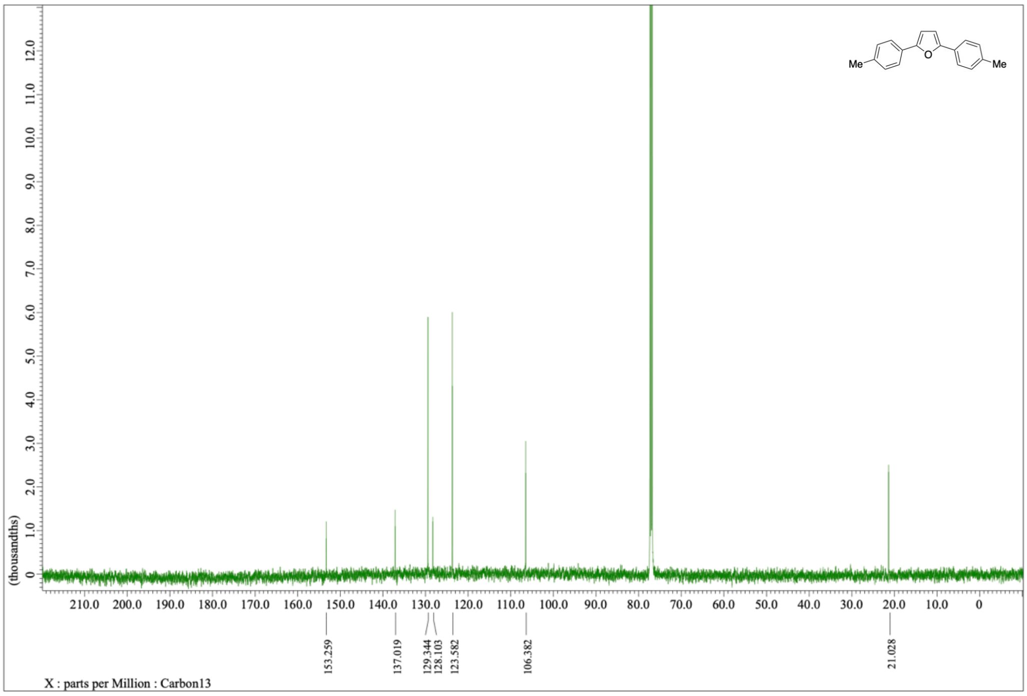

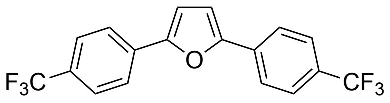

9c ^{1}H NMR ($CDCl_3$, 500 MHz)

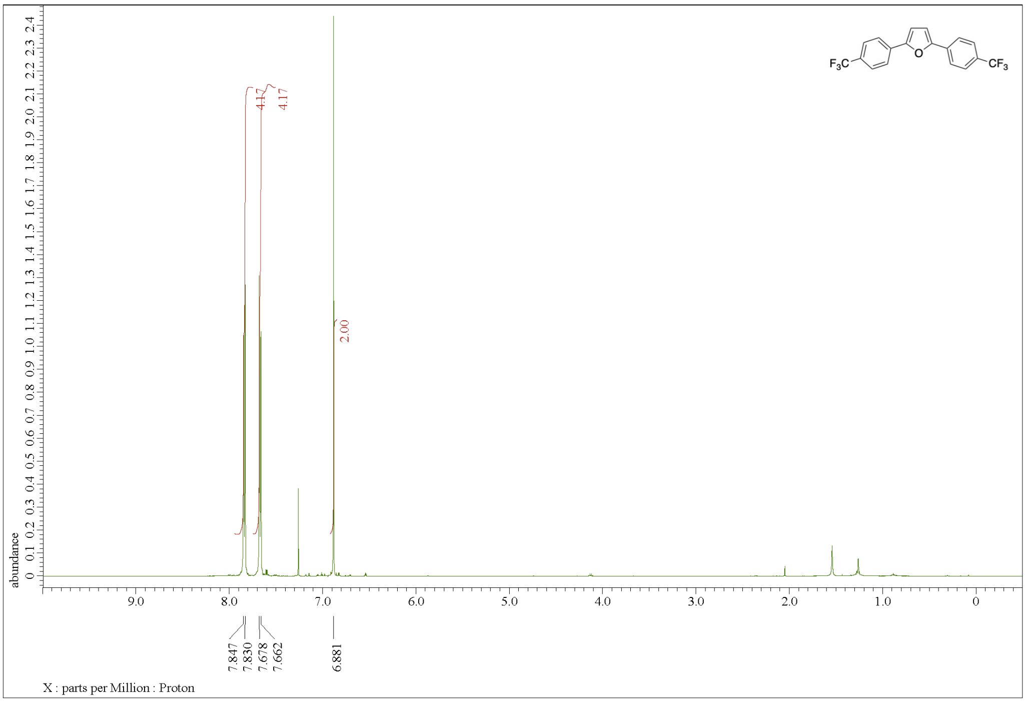

9c ^{13}C NMR ($CDCl_3$, 126 MHz)

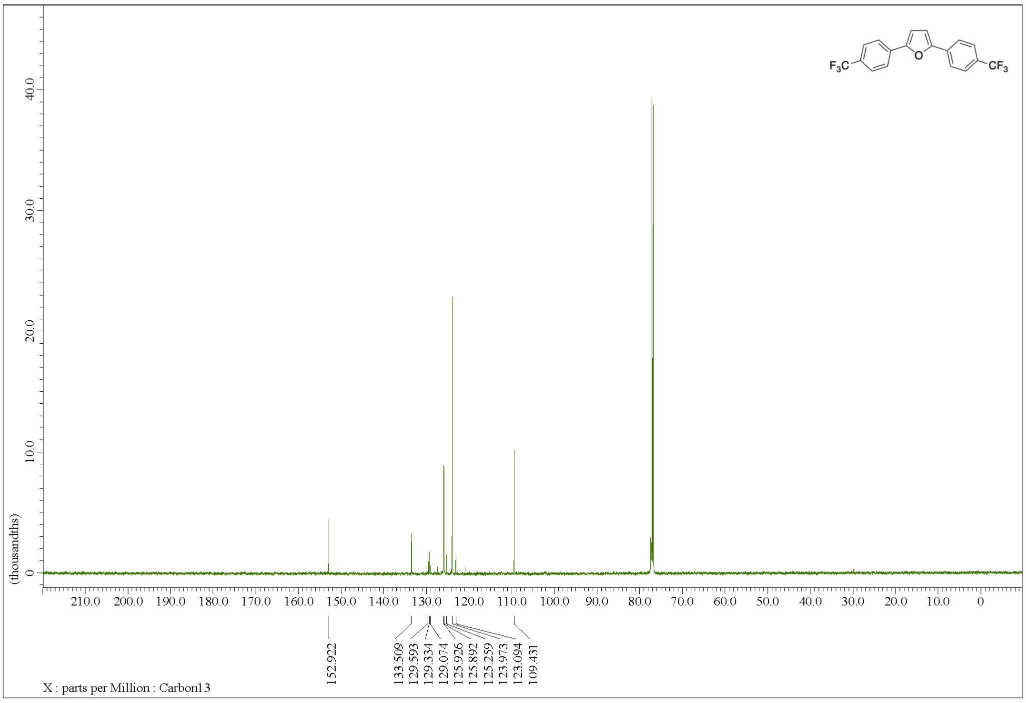

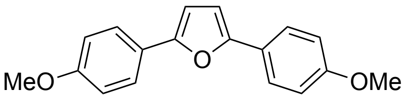

9d ^{1}H NMR ($CDCl_3$, 500 MHz)

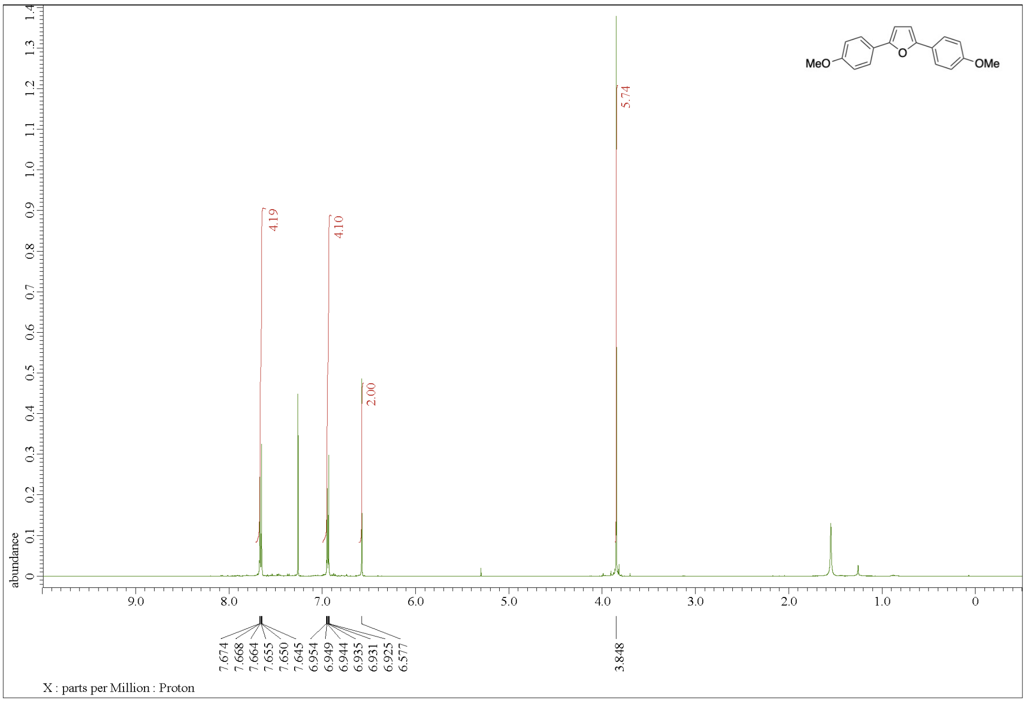

9d ^{13}C NMR ($CDCl_3$, 126 MHz)

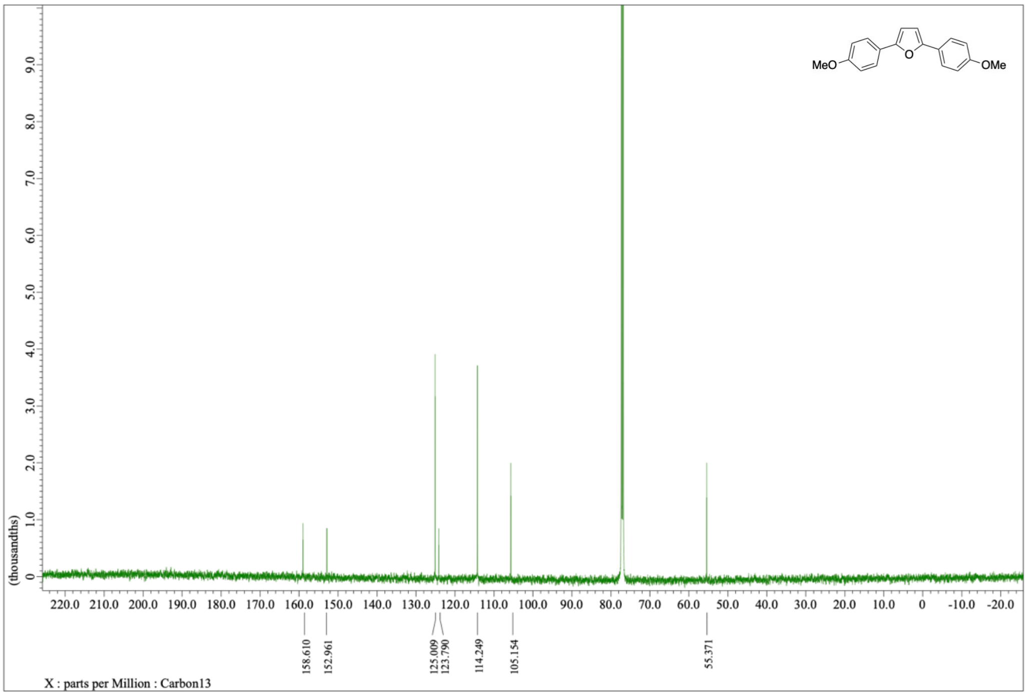

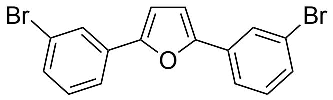

9e ^{1}H NMR ($CDCl_3$, 500 MHz)

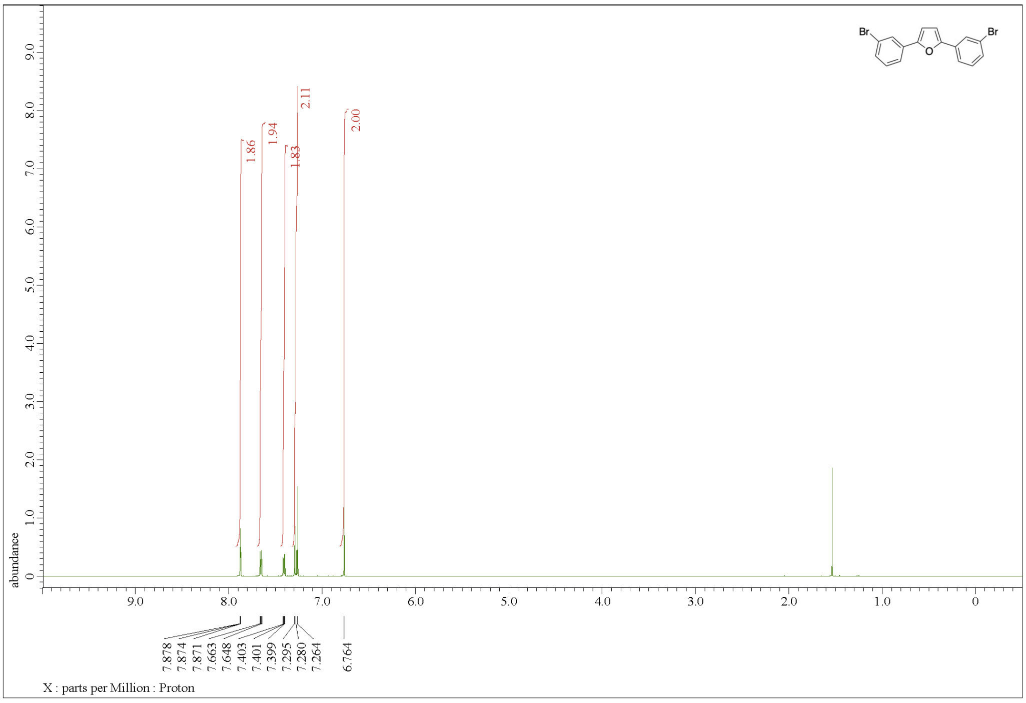

9e ^{13}C NMR ($CDCl_3$, 126 MHz)

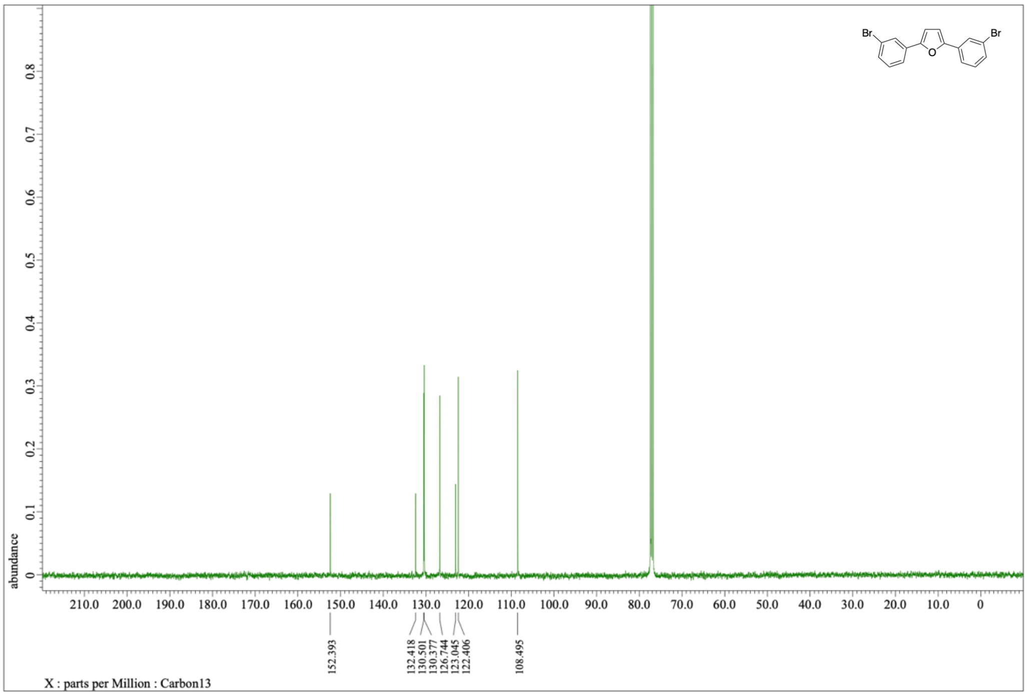

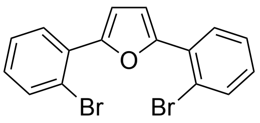

9f ^{1}H NMR ($CDCl_3$, 500 MHz)

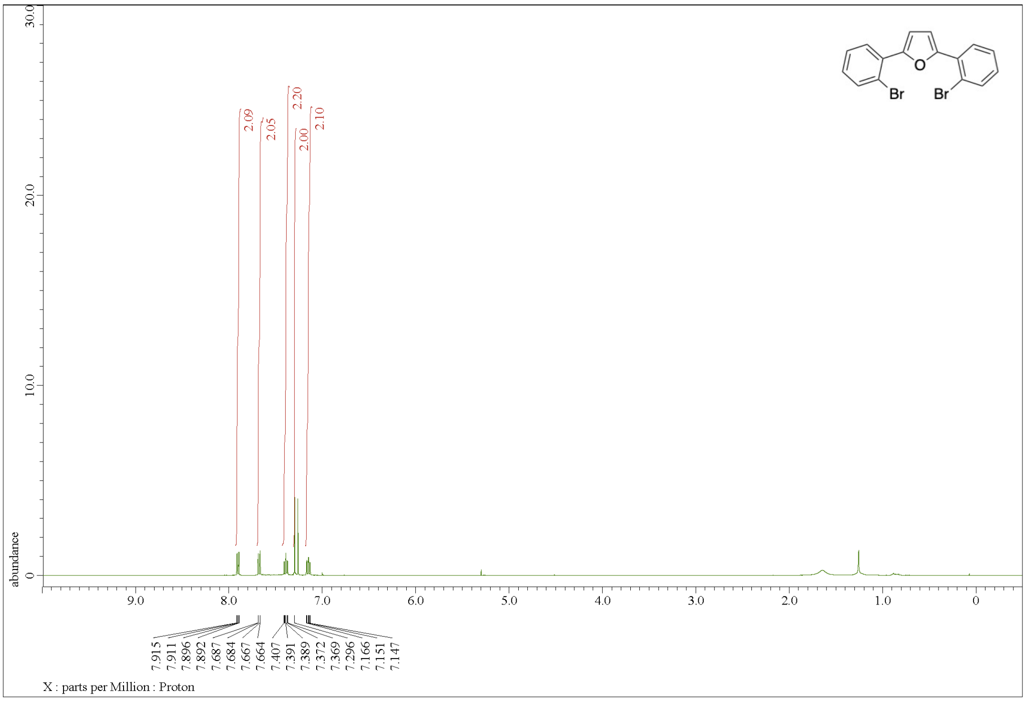

9f ^{13}C NMR ($CDCl_3$, 126 MHz)

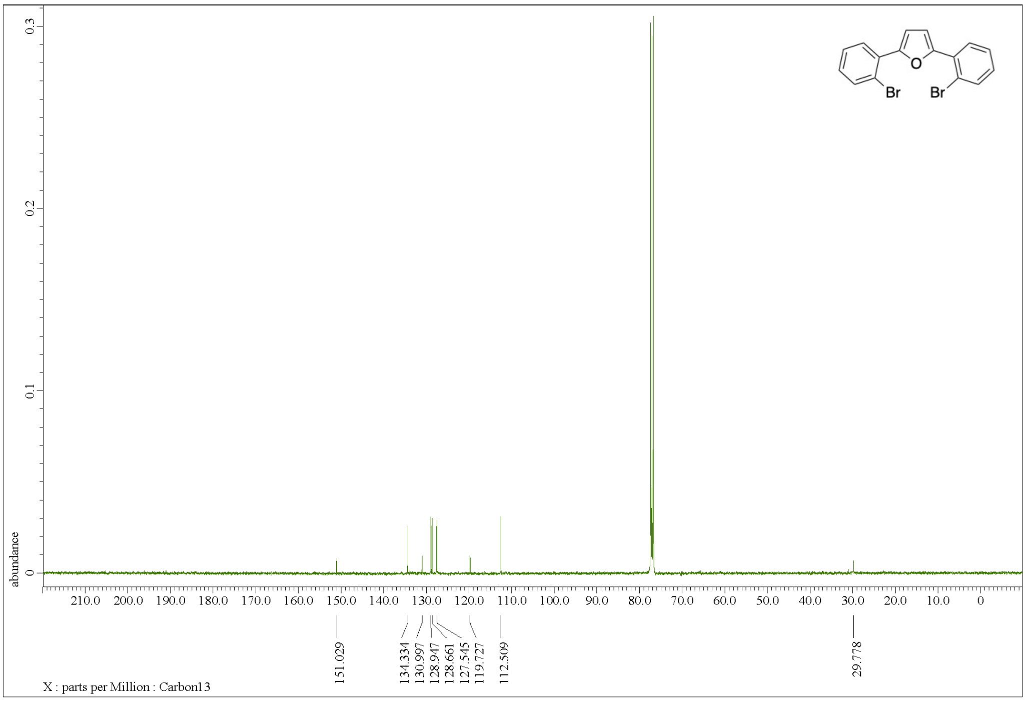

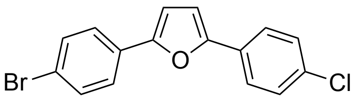

9h ^{1}H NMR ($CDCl_3$, 400 MHz)

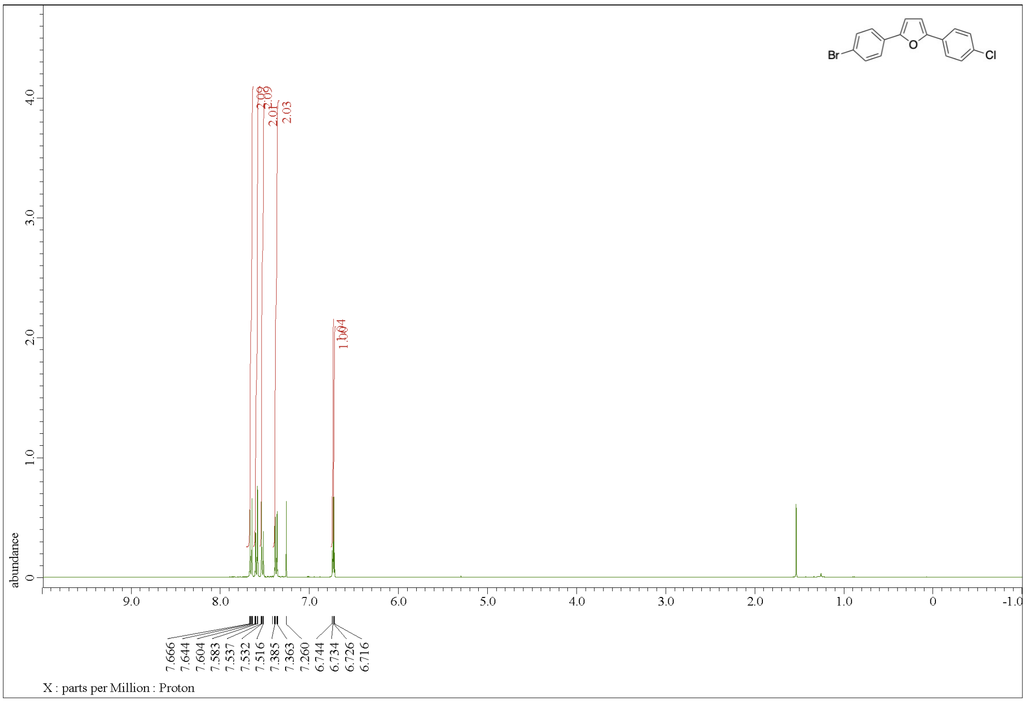

9h ^{13}C NMR ($CDCl_3$, 100 MHz)

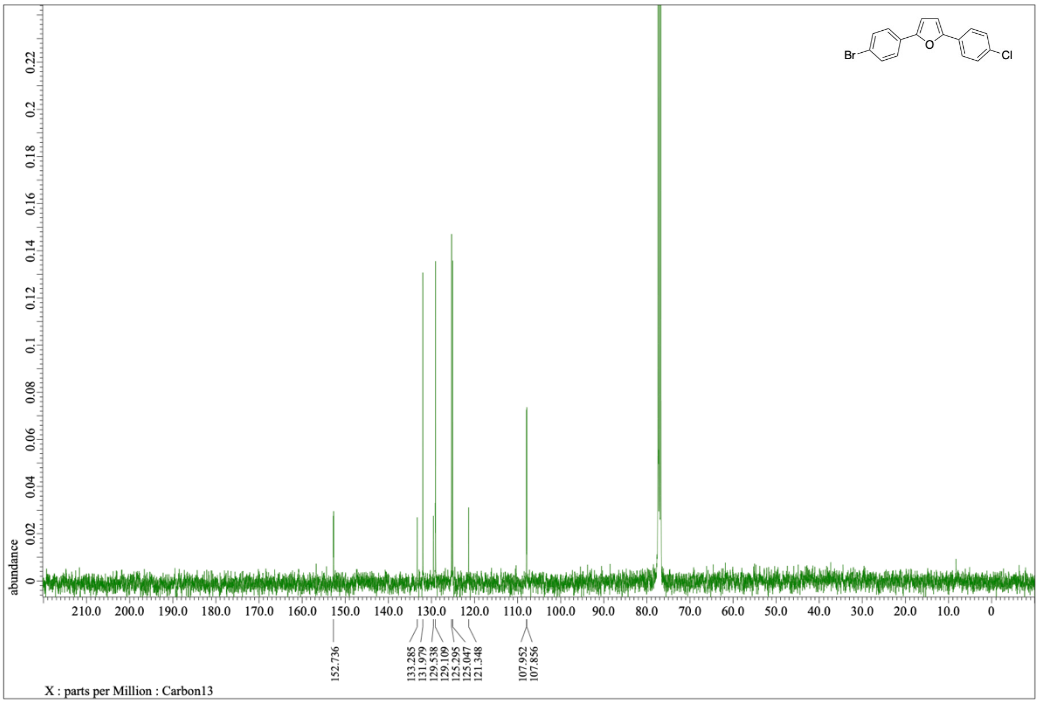

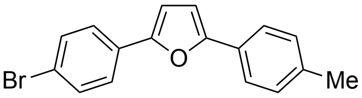

9i ^{1}H NMR ($CDCl_3$, 400 MHz)

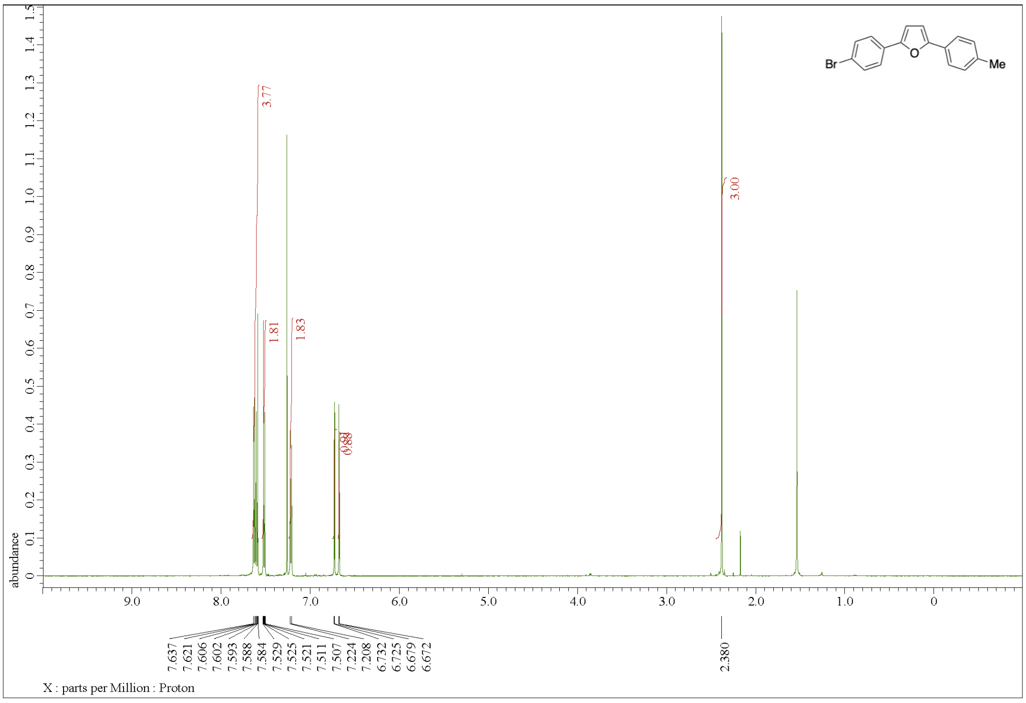

9i ^{13}C NMR ($CDCl_3$, 100 MHz)

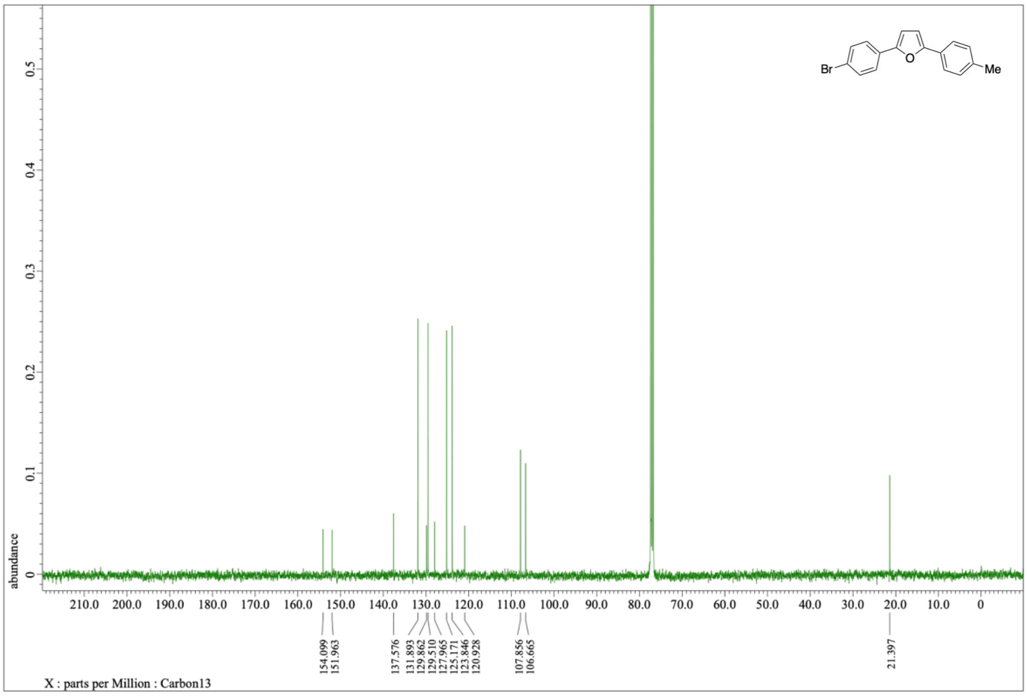

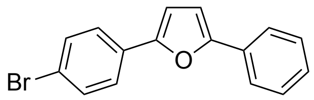

9j ^{1}H NMR ($CDCl_3$, 400 MHz)

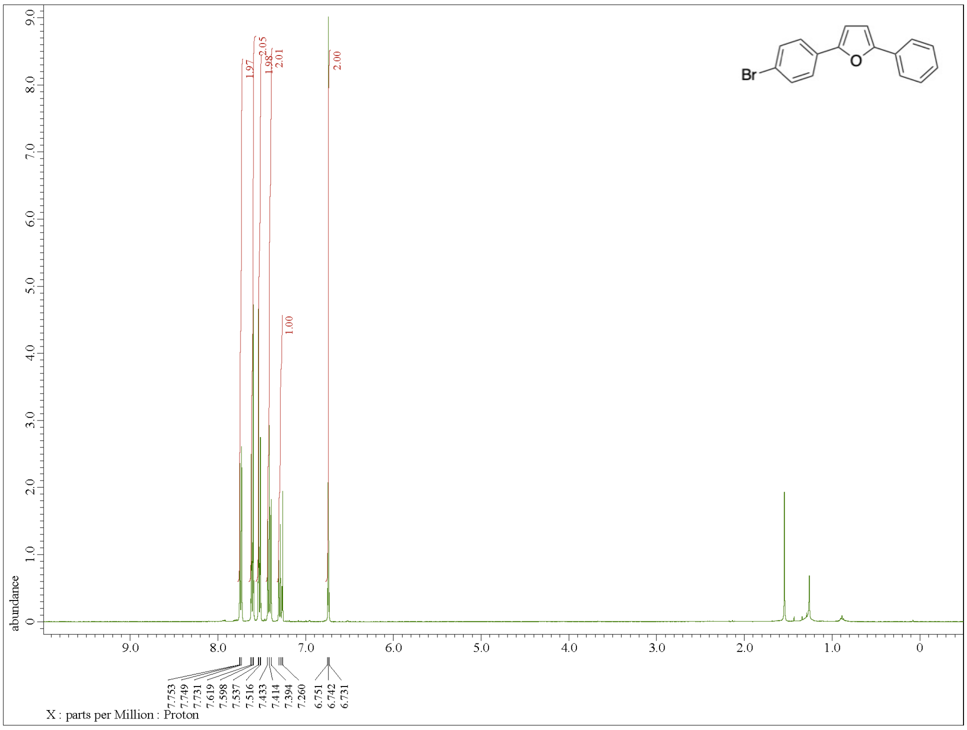

9j ^{13}C NMR ($CDCl_3$, 100 MHz)

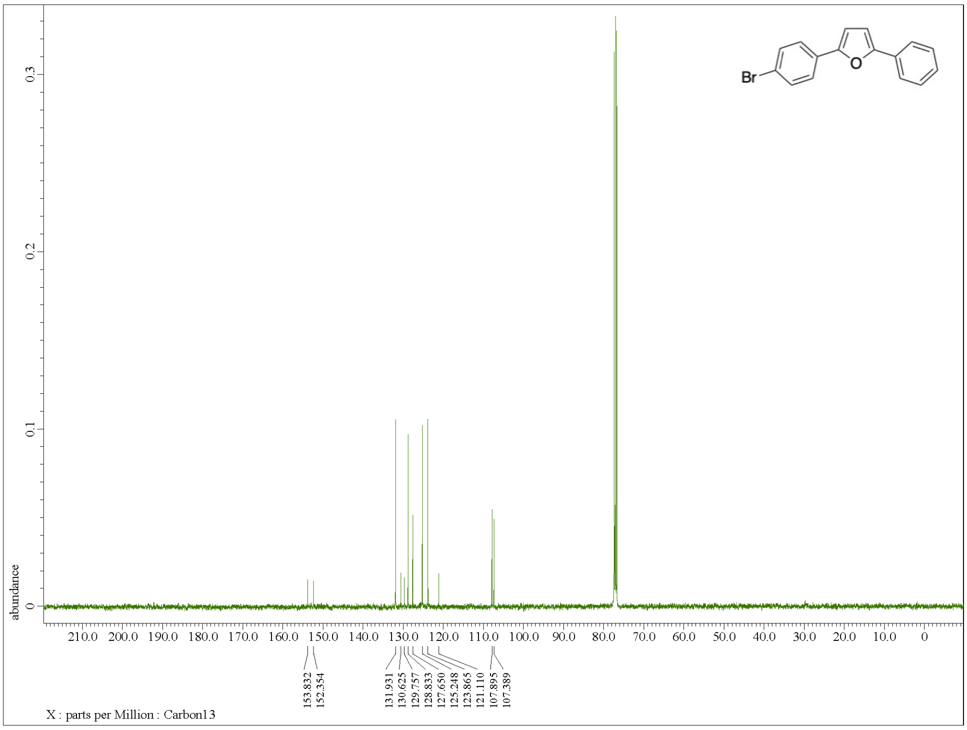

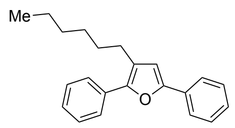

9k ^{1}H NMR ($CDCl_3$, 500 MHz)

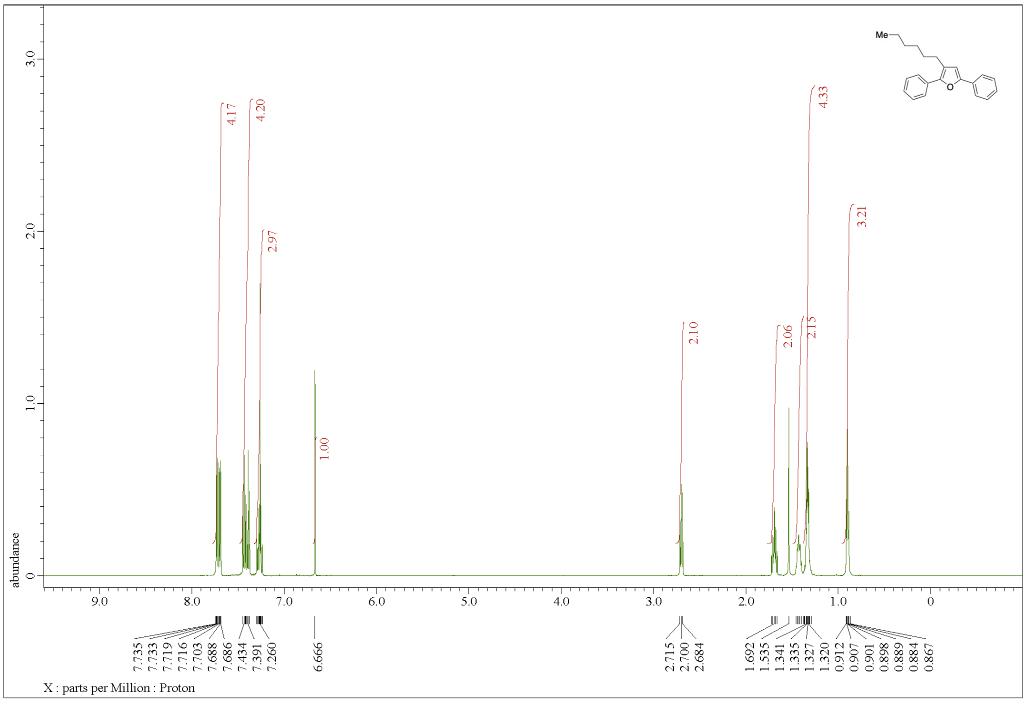

9k ^{13}C NMR ($CDCl_3$, 126 MHz)

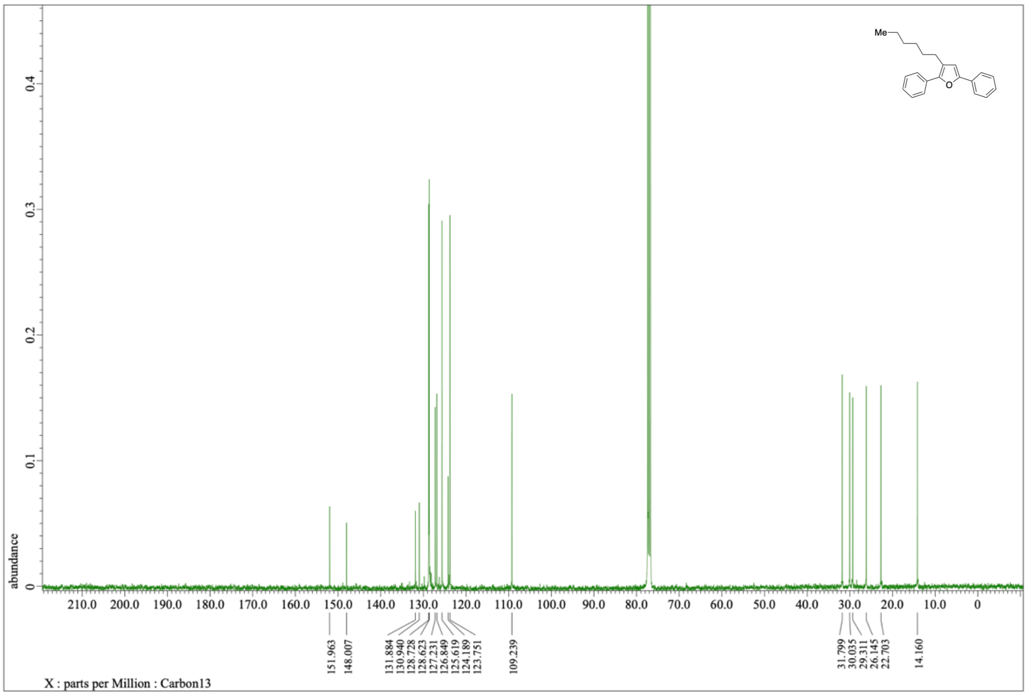

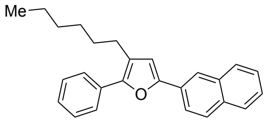

9l ^{1}H NMR ($CDCl_3$, 400 MHz)

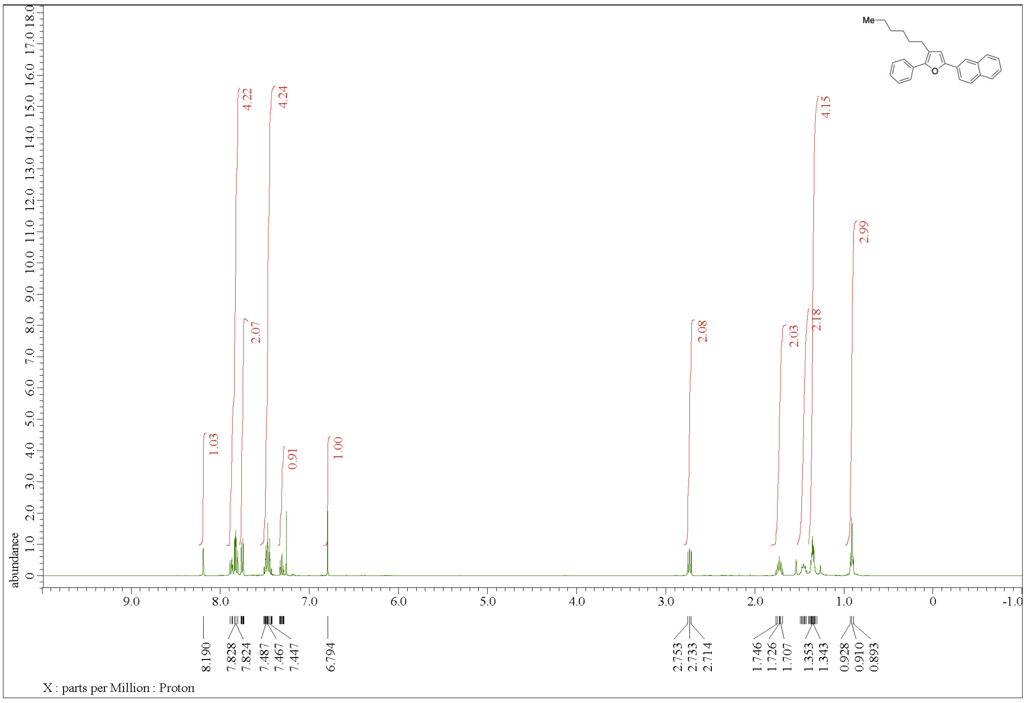

9l ^{13}C NMR ($CDCl_3$, 100 MHz)

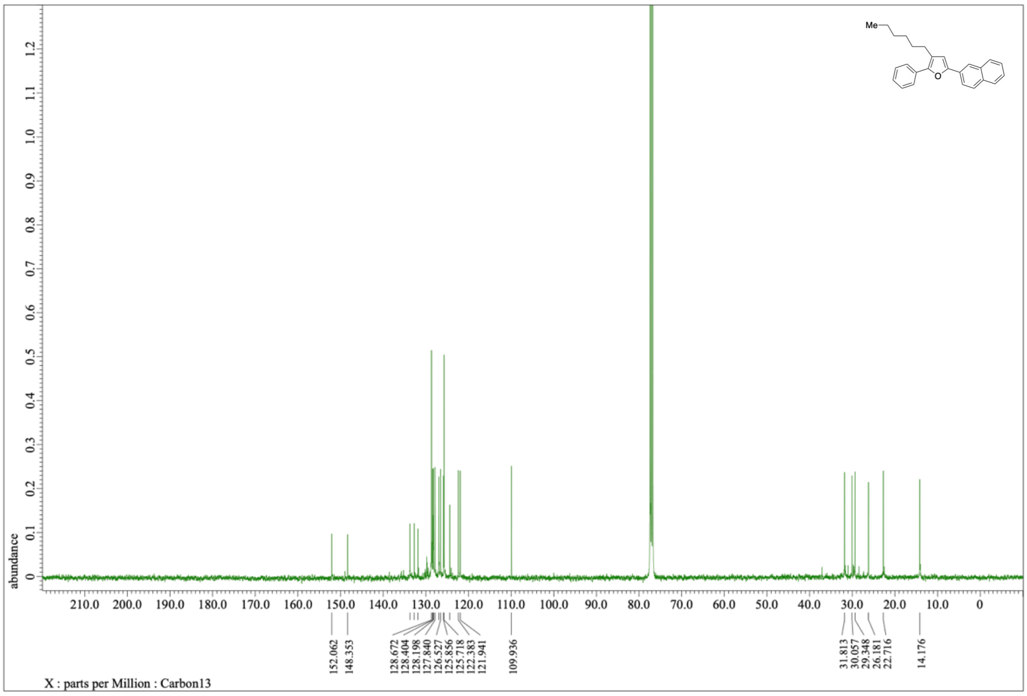

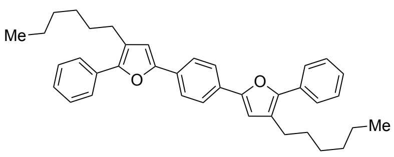

9m ^{1}H NMR (CDCl$_3$, 400 MHz)

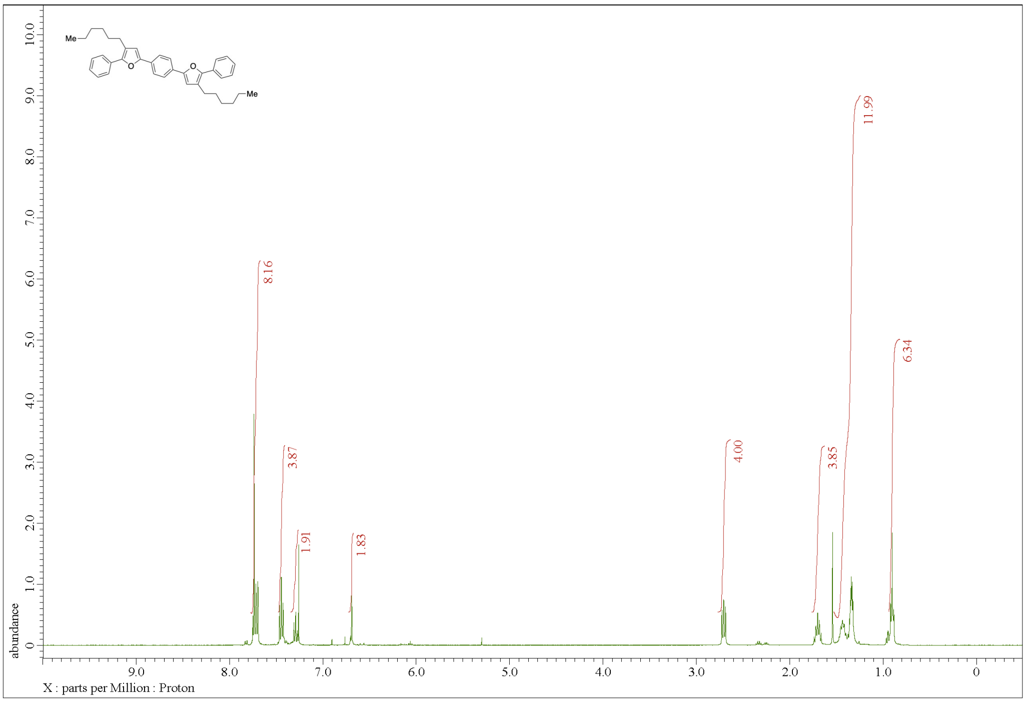

9m ^{13}C NMR (CDCl$_3$, 100 MHz)

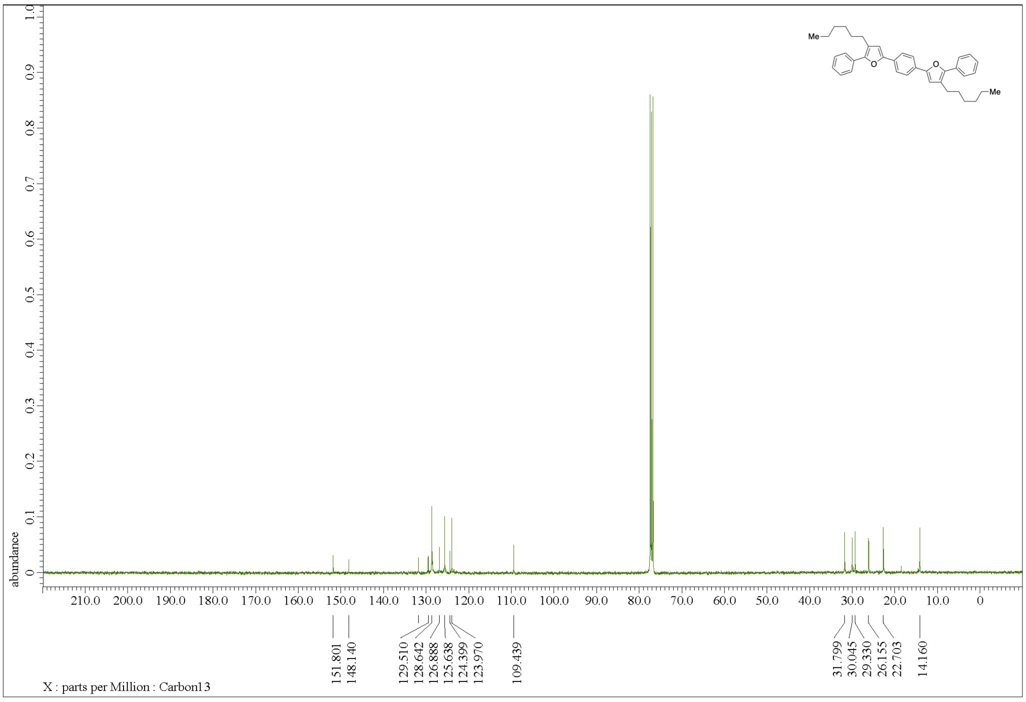

Supplement: Supplementary file 1 — jo3c02237_si_001.pdf [file jo3c02237_si_001.pdf]
